# Contrasting 50‐Year Trends of Moth Communities Depending on Elevation and Species Traits

**DOI:** 10.1111/ele.70195

**Published:** 2025-08-14

**Authors:** Felix Neff, Yannick Chittaro, Fränzi Korner‐Nievergelt, Glenn Litsios, Carlos Martínez‐Núñez, Emmanuel Rey, Eva Knop

**Affiliations:** ^1^ Agroecology and Environment Agroscope Zurich Switzerland; ^2^ info fauna Neuchâtel Switzerland; ^3^ Swiss Ornithological Institute Sempach Switzerland; ^4^ Department of Evolutionary Biology and Environmental Studies University of Zurich Zurich Switzerland

**Keywords:** abundance, biomass, elevation, functional traits, insects, lepidoptera, moths, richness, temporal trends

## Abstract

Following alarming studies on insect declines, evidence for contrasting patterns in temporal insect trends is growing. Differences in environmental conditions (e.g., climate), anthropogenic pressures (e.g., land‐use and climate change), and insect community composition may drive contrasting trends. With increasing elevation, these factors change quickly, which makes elevational gradients an ideal study case to disentangle their roles for differences in temporal trends. We thus analysed 2.8 million moth records collected in Switzerland. Fifty‐year trends (1972–2021) depended on local conditions and insect community composition: moth abundance, richness and biomass at low elevation decreased but increased at high elevation. These changes mainly concerned cold‐adapted, mono‐ and oligophagous, and pupal overwintering species, which shifted their ranges upwards. Our results point to climate change but also intensive land use and light pollution as drivers of moth community changes and suggest that high‐elevation habitats as refugia could be key to sustain moth diversity.

## Graphical abstract

This study, based on 2.8 million moth records in Switzerland from 1972 to 2021, shows that moth numbers decreased at low elevations but increased at high elevations. These changes mainly affected cold‐adapted, food‐specialised and pupal overwintering species. The findings point to climate change, but also land use and light pollution as drivers and demonstrate how temporal trends are affected by environmental conditions, anthropogenic pressures and insect community composition.

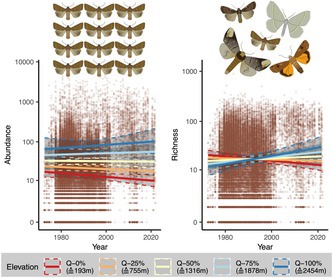

## 1 Introduction

Insect decline has become a major concern in recent years, with several studies showing strong decreases in insect richness, abundance, or biomass over just a few decades or even years (Habel et al. 2016; Hallmann et al. 2017; Seibold et al. 2019). These declines are particularly worrisome because insects are a seminal part of biodiversity and contribute to various ecosystem functions and services, such as pollination or pest control (Klein et al. 2007; Losey and Vaughan 2006). Thus, an increasing body of studies has addressed temporal trends in insect communities in recent years, some of which confirmed declines while others did not or even found increases (e.g., Dalton et al. 2023; Edwards et al. 2025; Evans et al. 2022; Klein et al. 2007; Macgregor et al. 2019). These results indicate that insect decline might strongly depend on insect traits, local environmental conditions, and the (anthropogenic) changes in these conditions acting on insect communities (Blüthgen et al. 2023; Vidal et al. 2025). Thus, studies on temporal trends in insect communities along gradients of changing local conditions offer an excellent opportunity to understand drivers of insect declines, but this has hardly been done because replicated datasets covering large temporal and environmental gradients simultaneously are very rare.

Many studies on insect decline focus on conspicuous, diurnal insect groups such as butterflies or bees (Goulson et al. 2008; Habel et al. 2022; Soroye et al. 2020; van Strien et al. 2019; Warren et al. 2021; Wepprich et al. 2019) and much less is known about other groups such as nocturnal insects. Moths are a major group of nocturnal insects, but their temporal trends remain little understood. Among the existing, mostly spatially strongly confined studies on temporal trends in moth communities, many show declines (Franzén and Johannesson 2007; Groenendijk and Ellis 2011; Hallmann et al. 2020; Roth et al. 2021), whereas other studies show more nuanced results (Macgregor et al. 2019; Valtonen et al. 2017; Wagner et al. 2021; Yazdanian et al. 2023) or even increases (Hunter et al. 2014). These partly contrasting temporal trajectories from different regions and time frames again point to differences in local conditions and the (anthropogenic) changes in these conditions to which the studied moth communities were exposed. Studies of temporal trends in moth communities along gradients of changing local conditions are needed to better understand the role of these different drivers.

Long‐term samples of moth communities along elevational gradients offer an excellent opportunity to study the influence of varying local conditions on temporal trajectories in moth communities due to drastically changing environmental conditions with elevation. In addition, the extent of anthropogenic pressures can vary with elevation, resulting in differences in the temporal changes in environmental conditions along elevational gradients. For instance, in mountain ranges of populated regions such as the Alps, many anthropogenic pressures tend to be stronger at lower elevations. Land‐use intensification and increasing light pollution—both important drivers of moth dynamics (Fox 2013; van Grunsven et al. 2020; Knop et al. 2017; Merckx and Van Dyck 2019)—are for example more prevalent at lower elevations. Furthermore, there is clear variation of insect community composition along elevation gradients (Beck et al. 2017; Hodkinson 2005). For example, climate in mountain ecosystems varies greatly with elevation, such as in terms of average temperature or precipitation, which results in insect communities of different elevations differing in their ability to cope with climatic variability and hence climate change (e.g., Neff et al. 2022). Given these changes in local conditions, anthropogenic pressures and in insect community composition with elevation, we expect clear dependences between temporal trajectories of moth communities and elevation; but this has so far not been studied.

Moths are a highly diverse group of insects with a wide range of ecological strategies. As such, they can exhibit various responses to environmental changes, which may be linked to specific traits that determine their responses to given local environmental conditions (Lavorel and Garnier 2002). Consequently, different moth species with different traits have shown contrasting temporal trajectories (cf. Coulthard et al. 2019). For example, declines have been more prominent among large species (Coulthard et al. 2019; Heidrich et al. 2021; but see Maes et al. 2024) and food‐specialised species (Franzén and Johannesson 2007; Roth et al. 2021; Valtonen et al. 2017; Wagner et al. 2021). Furthermore, in response to climate warming, certain areas witnessed decreases in cold‐adapted species and rises in warm‐adapted species (Fox et al. 2014; Maes et al. 2024). At the same time, the extent of range shifts in moth communities has been linked to the overwintering stage (Forsman et al. 2016; Keret et al. 2020; Mattila et al. 2006, 2008). This is because different life stages are more or less vulnerable to out‐of‐the‐norm climatic conditions (Zhang et al. 2015) and that these out‐of‐the‐norm conditions might be more or less prevalent depending on the season, for example, if summer temperatures rise disproportionately. To date, linking response traits to contrasting temporal trends in moths has remained elusive and has not been done along elevational gradients. Therefore, assessing species range shifts and the changes in moth community characteristics along an elevational gradient in relation to these different response traits will allow for a better understanding of the drivers of temporal changes.

**FIGURE 1:**
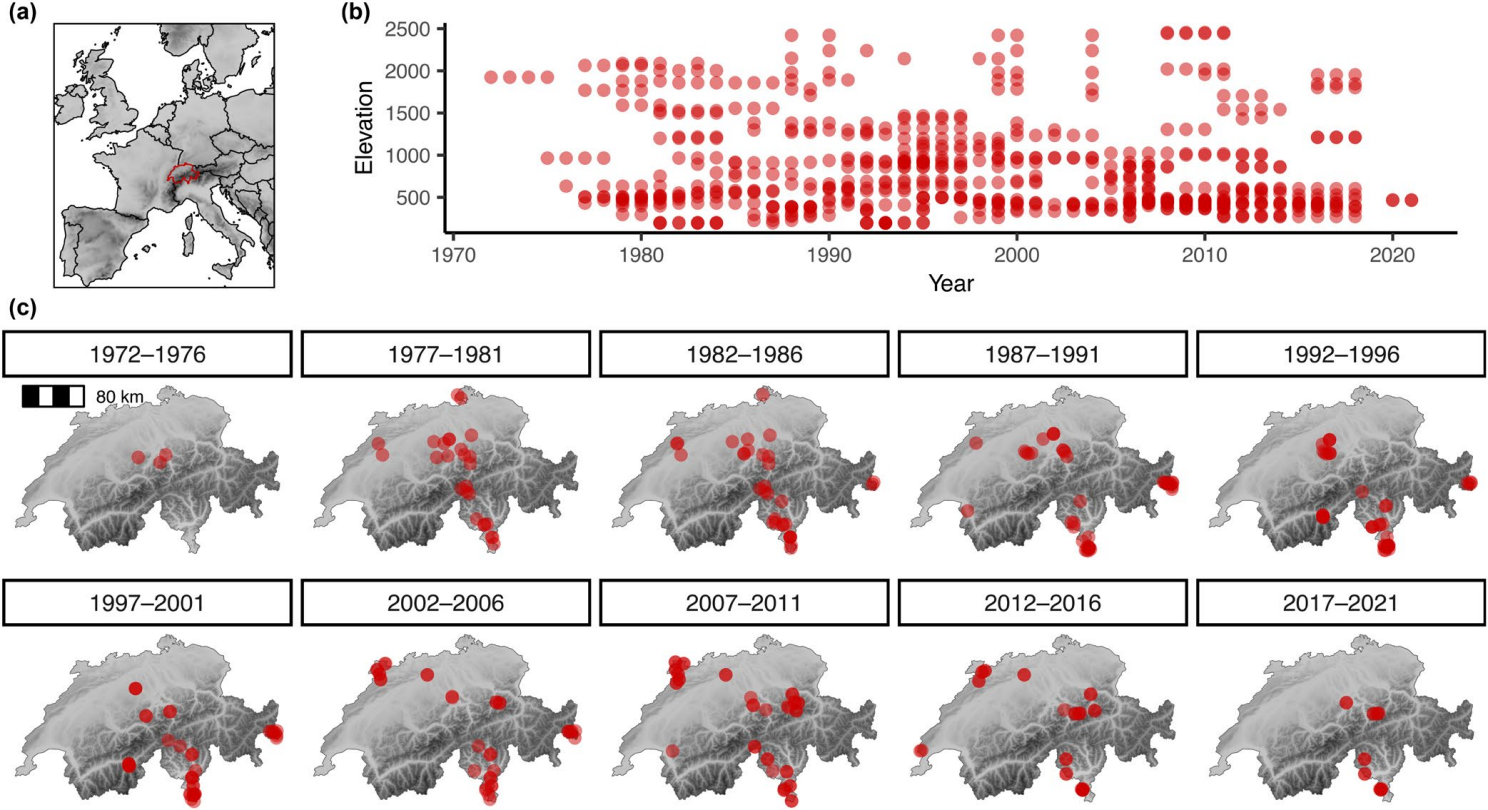
Sampling sites and their spatio‐temporal distribution. (a) Location of Switzerland within Europe, (b) spread of sampling sites across the elevational gradient in dependence on sampling year, (c) spatial distribution of sampling sites within Switzerland for consecutive 5‐year intervals. In the maps, the shading shows elevation. Elevation data from Hengl et al. (2020) and the Federal Office of Topography swisstopo.

Here, we analysed a unique dataset on moth communities (species‐level and total abundance, species richness, biomass) from Switzerland spanning a large temporal (1972–2021) and elevational (193–2454 m above sea level) gradient (Figure 1). We asked (i) how moth community characteristics (species‐level and total abundance, richness, biomass) changed across the last 50 years and how these changes depended on elevation and (ii) how temporal changes and their dependence on elevation differed among species groups with different traits (body size, temperature niche, food specialisation, overwintering stage). We show that trends depend on elevation, with decreases at low elevations and increases at high elevations. Patterns were different between groups defined by traits, particularly by temperature niche and overwintering stage, indicating the important role of climate change in driving the observed changes in moth communities across the past 50 years.

## 2 Material and Methods

### 2.1 Moths Dataset

The data originate from light‐trap samplings spanning 50 years (1972–2021), which were collected by Dr. Ladislaus Rezbanyai‐Reser (ZOBODAT 2014–2025) at 171 sites across Switzerland (663 unique site and year combinations; Figure 1, Figures S1–S2) (cf. Rezbanyai‐Reser 2018). The dataset is hosted by *info fauna* (The Swiss Topic Centre on Fauna) and entails abundance data for 556,969 species occurrence records, representing 2,814,187 nocturnal macro‐moth individuals (1045 species) (Table S1). The dataset is accessible through the GBIF database (GBIF.org 2024). Data cover an elevational gradient between 193 and 2454 m above sea level (asl) (Figure 1b). As the dataset originates from one expert who operated in a standardised scheme, it provides valuable information on long‐term changes in moth communities. Still, there were some specifics that varied among samplings, which needed to be accounted for in the analyses. There were manual traps that were installed for single nights and fixed traps that were running for stretches of normally several months (Figures S1–S2). Also, different amounts of traps (between 1 and 4, Figure S3) and different lamp types (Figure S4) were in place. Manual traps were not always active for the whole night, but the sampling duration varied between 1 and 13 h. A total of 35,847 sampling nights could be analysed, which were spread across the entire year covering all seasons (Figure S5). A more detailed description of the dataset and the procedures used by Ladislaus Rezbanyai‐Reser is given in Appendix S1.

### 2.2 Trait Data

We used species‐level data on forewing length to estimate dry mass for all study species based on a set of recently published allometric relationships (Kinsella et al. 2020). Data on wingspan, which was more readily available, were extracted from an online database (Jonko 2002–2024) (*n* = 981) and supplemented with data from other sources (Fibiger 1990; Potocký et al. 2018; Ronkay et al. 2001) (*n* = 12). Because we needed forewing length instead of wingspan to estimate dry mass, we used forewing length data available for a restricted subset of study species (Cook et al. 2022) to fit a linear model relating wingspan to forewing length. We used this model to estimate forewing length of all species, which we then used to estimate dry mass based on the model proposed by Kinsella et al. (2020). For a few species (*n* = 13), we could not retrieve wingspan data. For these cases, we estimated dry mass from the average estimated dry mass of congeneric species. With our approach, we assumed dry mass per species to be constant among the studied temporal and spatial gradients, which might not generally be the case (e.g., Hill et al. 2021; Merckx et al. 2018), but we expected interspecific differences to outweigh such intraspecific differences.

We determined each species' temperature niche following the Species Temperature Index approach (Devictor et al. 2008). We used distributional records from the GBIF database (GBIF.org, https://doi.org/10.15468/dl.2mev52, https://doi.org/10.15468/dl.km9rkn). To reduce sampling bias, we only included records from Europe and accumulated them at the grid defined by the Common European Chorological Grid Reference System (CGRS) (50 × 50 km cells). We used mean temperature (1970–2000) values from WorldClim 2 (Fick and Hijmans 2017) at a 2.5 min spatial resolution, which we aggregated at the CGRS grid. Finally, we quantified a species' temperature niche as the mean temperature of the CGRS grid cells with records of this species.

We categorised all species into three levels of feeding specialisation of larvae (monophagous, oligophagous, and polyphagous) based on various sources on species' feeding specialisation (Cook et al. 2022) and on species' food plants (Hacker and Müller 2006; Lepiforum e.V 2002–2021; Pearse and Altermatt 2013; Steiner et al. 2014). We defined monophagous species as species feeding on plant species of a single genus, oligophagous species as species feeding on several genera within a single family, and polyphagous species as species feeding on plants of several families. We obtained overwintering stages of the studied species (egg, larva, pupa, and adult) from several trait collections (Cook et al. 2022; Mangels et al. 2017; Potocký et al. 2018) and corrected and completed them based on other moth trait databases (Jonko 2002–2024; Ziegler 2005–2024).

### 2.3 Statistical Analyses

For all statistical analyses, we used R version 4.2.0 (R Core Team 2022). All codes relevant for the analyses as well as additional data necessary to reproduce the analyses are deposited in a repository available from Zenodo (Neff et al. 2025a).

For each of the 35,847 sampling nights, we determined total abundance (sum of individuals across all species), species richness, and total biomass (estimated from species‐level dry mass, see section on species traits). We used species richness corrected for sample coverage to account for sampling differences, as estimated with the ‘iNEXT’ package (Hsieh et al. 2024). These three community characteristics were the response variables in a series of regression models (generalised linear mixed models). For total abundance, we used a zero‐inflated negative binomial response distribution (log link) while for richness and biomass, we used a hurdle gamma distribution (log link) (Figure S6). The explanatory variables of primary interest in our analyses were the study year, the elevation (mean elevation of the study site measured at a hectare) and the interaction of year and elevation. While a non‐linear relation of community characteristics to elevation is often encountered (Rahbek 2005), previous analyses of the present dataset showed that across the studied elevational gradient, the relation of community characteristics and elevation is close to linear (Neff et al. 2025b).

Besides the main terms, the models included a set of other fixed effects to account for sampling timing and design. In terms of the timing of sampling, we accounted for the season by including the day of the year as a smoothing term (Figure S5) as well as for weather conditions. We determined the weather of each sampling night based on a gridded daily temperature and precipitation dataset (1.25 min grid; approx. 2.3 × 1.6 km) provided by MeteoSwiss (https://www.meteoswiss.admin.ch). For each location, we used data from the closest grid cell and extracted daily mean temperature and precipitation values for the two days enclosing the sampling night. Then, we used two‐day averages (mean temperature) and two‐day sums (precipitation) as weather variables for each sampling night. To account for sampling design, we included trap type (fixed type 1, fixed type 2, or manual; Figure S2), lamp type (four nominal factor levels; Figure S4), number of traps (ordinal factor with four levels; Figure S3), a two‐level nominal factor for whether there was sampling in the previous night, and sampling duration (smoothing term) as predictor variables. We only included sampling duration for manual traps, as fixed traps were active all night. For 1224 sampling nights of manual traps (out of 4024), no information on sampling duration was available and we assumed a constant effect. We decided against a linear effect of sampling duration due to changing moth activity patterns across the course of a night (e.g., Ma and Ma 2013).

We used standard spline procedures of additive models for all smoothing terms. We standardised all continuous predictor variables to mean 0 and standard deviation 1 and used sum‐to‐zero contrasts for nominal factor variables. Additionally, we included random terms accounting for the site (*n* = 171), the site–year combination (*n* = 663), the sampling night with simultaneously operated sites grouped together (*n* = 34,390), and a term for the spatio‐temporal clustering of sites (*n* = 249). We defined the latter grouping variable based on distances between sampling locations within a year, with all locations within 20 km of each other being grouped. This allowed us to account for similarity in moth communities within years between close sites, while not grouping locations across biogeographic barriers such as across high mountain ranges. The results for the model covariates that were not the main focus of the analyses (i.e., all variables except for year and elevation) are qualitatively equal to those in a related study with another focus and different models, which is based on the same dataset (Neff et al. 2025b). Thus, we only report them in the Supporting Information (cf. Figures S7–S9), but do not discuss them in detail.

In a first step, we fitted the abundance, richness, and biomass models to the whole dataset. Second, to analyse how trait composition of moth communities changed across the last 50 years, we determined total abundance, sample‐coverage corrected richness, and estimated biomass for separate species groups, which we defined by different traits, i.e., body size (estimated dry mass), temperature niche, food specialisation, and overwintering stage (Table S1). For continuous traits, we defined the three groups by the 33% and 66% quantiles, which we determined across all study species (i.e., each group containing one third of the recorded species). Then, we used the regression models to analyse temporal trends in relation to elevation for abundance, richness, and biomass of these trait groups. Third, to check whether community‐level changes were also reflected in changes in the distribution of single species, we fitted models with the same structure as the community‐level abundance model for single species. We determined the abundance of the focal species for each sampling occasion, which we used as the response variable thereafter. Because single‐species models are less meaningful for rare species, we only included species that were recorded in at least 100 unique combinations of site and year (out of 663 possible combinations). Accordingly, we fitted 442 single‐species models (out of 1045 species recorded in total; representing 93.6% of individuals recorded). From these models, we extracted the model coefficient estimates for the interaction between year and elevation, which we then related to the species trait groups (body size, temperature niche, food specialisation, and overwintering stage).

We used ‘brms’ (Bürkner et al. 2022) to build the structure of the models and then manually adapted the underlying Stan code for more flexibility. We then fitted these models in Stan version 2.26.1 (Stan Development Team 2021) through ‘rstan’ (Guo et al. 2023) (4 Markov chain Monte Carlo [MCMC] chains with 2000 iterations each, including 1000 warm‐up iterations) and monitored the mixing of the four MCMC chains through the Rhat statistic, calculated with ‘rstan‘ (Guo et al. 2023). Estimates of intercepts, fixed effect slopes and spline coefficients (smoothing terms) were below the standard threshold of 1.1 for all community‐level models, showing that chains mixed well. We specified priors following the defaults of the ‘brms’ package (details available in the online repository for the code). We used means and symmetric credible intervals (CIs) to summarise posterior distributions and evaluated model results based on model predictions. To illustrate interactions of temporal trends in community‐level metrics with elevation, we calculated predictions across the temporal range for different elevations (e.g., minimum and maximum elevation). In a set of sensitivity analyses, we confirmed the robustness of our main study outcomes (Appendix S2).

## 3 Results

### 3.1 50‐Year Moth Trends Along Elevational Gradients

**FIGURE 2:**
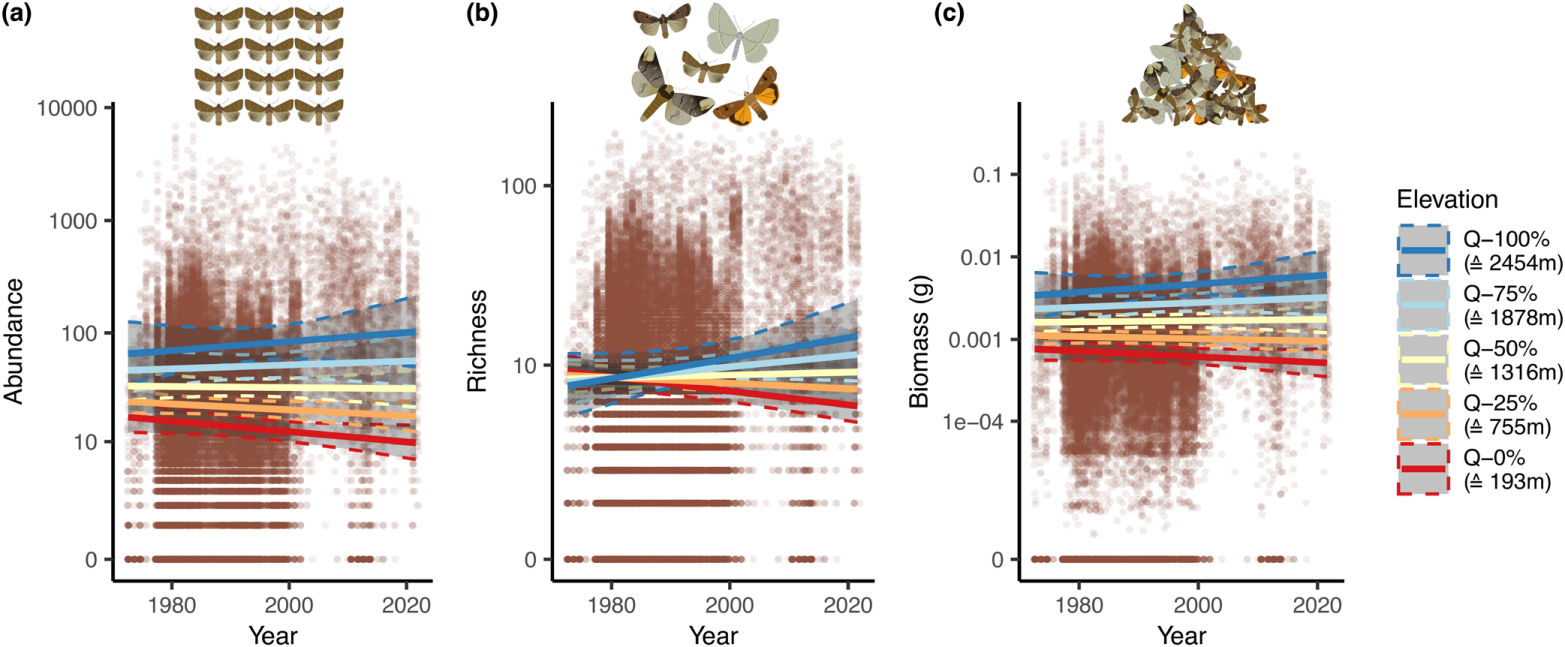
Change in moth (a) abundance, (b) richness, and (c) biomass across the 50 study years (1972–2021) in relation to the elevation of the study site. Lines are point estimates from model predictions (conditional effects of elevation, year and their interaction), shaded areas show 95% credible intervals. To illustrate the interactive effect, separate trend predictions are shown for different quantiles (0%, 25%, 50%, 75%, and 100%) of the elevational range. The corresponding elevations are indicated as well, for example, 193 m asl for the 0%‐quantile (minimum elevation covered in the data). Underlying points show data per sampling night (*n* = 35,847), with which models were fitted. Note that the *y* axes are on a log scale (after adding the minimal non‐zero value to all values). More detailed model results are in Table S2 and Figures S7–S9.

There was no overall change in community characteristics (total abundance, sample‐coverage corrected richness, estimated biomass) across the 50 study years, but changes differed along the elevational gradients (Figure 2, Table 1, Tables S2 and S3). Abundance, richness, and biomass were generally higher at higher elevations and increased over the study period (Figure 2, Table S2). At lower elevations, they were lower and decreased further (Figure 2, Table S2). Effect sizes of these community characteristic changes in relation to elevation were relatively high (Table 1, Table S3): At the lowest elevations, the decrease in abundance was estimated to a factor of 0.596 (95% CI: 0.323 to 1.02), corresponding to a decrease of 40.4% (95% CI: −67.7% to +2.45%) (percentage changes throughout relate to the estimated value at the beginning of the study period) over the 50 years of the study. Similar results were observed for richness (factor of 0.619 [95% CI: 0.411 to 0.887], corresponding to a decrease of 38.1% [95% CI: −58.9% to −11.3%]) and biomass (factor of 0.711 [95% CI: 0.396 to 1.21], corresponding to a decrease of 28.9% [95% CI: −60.4% to +20.6%]). At the highest elevation, in turn, the increase was estimated to a factor of 1.89 (95% CI: 0.478 to 5.11), corresponding to an increase of 89.4% (95% CI: −52.2% to +411%), for abundance, a factor of 2.83 (95% CI: 1.16 to 5.86), corresponding to an increase of 183% (95% CI: +15.6% to +486%), for richness, and a factor of 2.03 (95% CI: 0.568 to 5.44), corresponding to an increase of 103% (95% I: −43.2% to +444%), for biomass. We also found the positive interaction between year and elevation in the majority of the single‐species models. In 74.1% (95% CI: 71.0% to 77.1%) of these models, the coefficient for the interaction was positive, indicating more positive temporal trends at higher elevations.

**TABLE 1:** Average change in total abundance and species richness across the 50 study years based on model predictions in relation to elevation and for different species groups defined by traits.

Resp.	Trait	Trait value	Elevation			Threshold elevation (m asl)
			Lowest	Median	Highest	
Abundance	Full data		0.596 (0.323 to 1.02) −40.4% (−67.7% to +2.45%)	0.991 (0.528 to 1.69) −0.937% (−47.2% to +68.6%)	1.89 (0.478 to 5.11) +89.4% (−52.2% to +411%)	1889 (<min to >max)
	Body size	Small	0.371 (0.166 to 0.771) −62.9% (−83.4% to −22.9%)	0.832 (0.339 to 1.64) −16.8% (−66.1% to +64.3%)	2.39 (0.336 to 8.42) +139% (−66.4% to +742%)	556 (<min to >max)
		medium	0.594 (0.286 to 1.07) −40.6% (−71.4% to +7.00%)	1.55 (0.768 to 2.87) +54.6% (−23.2% to +187%)	4.90 (0.947 to 16.1) +390% (−5.26% to +1506%)	984 (<min to 2124)
		Large	0.559 (0.279 to 1.01) −44.1% (−72.1% to +1.48%)	1.32 (0.691 to 2.30) +32.4% (−30.9% to +130%)	3.67 (0.855 to 10.4) +267% (−14.5% to +936%)	910 (<min to >max)
	Temperature niche	Cold	0.144 (0.0689 to 0.268) −85.6% (−93.1% to −73.2%)	0.732 (0.335 to 1.40) −26.8% (−66.5% to +40.4%)	4.61 (0.795 to 15.6) +361% (−20.5% to +1465%)	1669 (1146 to >max)
		Interm.	0.541 (0.285 to 0.958) −45.9% (−71.5% to −4.18%)	1.33 (0.671 to 2.33) +33.0% (−32.9% to +133%)	3.81 (0.873 to 10.7) +281% (−12.7% to +967%)	1180 (<min to >max)
		Warm	1.72 (0.665 to 3.80) +72.0% (−33.5% to +280%)	1.20 (0.419 to 2.67) +20.1% (−58.1% to +167%)	1.19 (0.108 to 4.70) +18.5% (−89.2% to +370%)	>max (<min to >max)
	Food specialisation	monoph.	0.234 (0.0938 to 0.475) −76.6% (−90.6% to −52.5%)	0.699 (0.286 to 1.45) −30.1% (−71.4% to +44.9%)	2.81 (0.362 to 10.6) +181% (−63.8% to +957%)	2211 (937 to >max)
		Oligoph.	0.343 (0.156 to 0.653) −65.7% (−84.4% to −34.7%)	1.18 (0.543 to 2.29) +18.3% (−45.7% to +129%)	4.98 (0.900 to 15.8) +398% (−9.96% to +1478%)	1390 (695 to >max)
		Polyph.	0.710 (0.384 to 1.22) −29.0% (−61.6% to +22.3%)	1.06 (0.562 to 1.83) +6.28% (−43.8% to +82.6%)	1.83 (0.455 to 5.10) +83.4% (−54.5% to +410%)	918 (<min to >max)
	Overwintering stage	Egg	0.476 (0.199 to 0.946) −52.4% (−80.1% to −5.45%)	1.21 (0.508 to 2.48) +21.3% (−49.2% to +148%)	4.0 (0.567 to 14.8) +300% (−43.3% to +1375%)	1325 (<min to >max)
		Larva	0.938 (0.444 to 1.74) −6.23% (−55.6% to +74.2%)	0.883 (0.418 to 1.65) −11.7% (−58.2% to +65.4%)	1.00 (0.197 to 3.09) +0.348% (−80.3% to +209%)	>max (<min to >max)
		Pupa	0.362 (0.174 to 0.672) −63.8% (−82.6% to −32.8%)	1.57 (0.754 to 3.0) +57.1% (−24.6% to +200%)	8.33 (1.62 to 26.5) +733% (+61.7% to +2554%)	1059 (596 to 1707)
		Adult	0.726 (0.275 to 1.57) −27.4% (−72.5% to +56.6%)	0.496 (0.181 to 1.08) −50.4% (−81.9% to +7.96%)	0.458 (0.0486 to 1.75) −54.2% (−95.1% to +75.1%)	695 (<min to >max)
Richness	Full data		0.619 (0.411 to 0.887) −38.1% (−58.9% to −11.3%)	1.28 (0.858 to 1.86) +28.1% (−14.2% to +86.3%)	2.83 (1.16 to 5.86) +183% (+15.6% to +486%)	1041 (448 to 1797)
	Body size	Small	0.622 (0.400 to 0.922) −37.8% (−60.0% to −7.77%)	1.18 (0.745 to 1.75) +17.8% (−25.5% to +75.4%)	2.40 (0.933 to 5.15) +140% (−6.67% to +415%)	1240 (365 to >max)
		medium	0.631 (0.405 to 0.927) −36.9% (−59.5% to −7.32%)	1.16 (0.727 to 1.73) +16.4% (−27.3% to +73.1%)	2.30 (0.876 to 4.98) +130% (−12.4% to +398%)	1398 (351 to >max)
		Large	0.591 (0.390 to 0.862) −40.9% (−61.0% to −13.8%)	1.25 (0.829 to 1.82) +24.7% (−17.1% to +82.3%)	2.81 (1.16 to 5.89) +181% (+15.7% to +489%)	1083 (516 to 1857)
	Temperature niche	Cold	0.246 (0.154 to 0.382) −75.4% (−84.6% to −61.8%)	0.792 (0.489 to 1.21) −20.8% (−51.1% to +21.1%)	2.78 (0.967 to 6.33) +178% (−3.26% to +533%)	1645 (1185 to >max)
		Interm.	0.556 (0.373 to 0.794) −44.4% (−62.7% to −20.6%)	1.42 (0.953 to 2.03) +42.1% (−4.72% to +103%)	3.85 (1.65 to 7.64) +285% (+65.1% to +664%)	938 (552 to 1413)
		Warm	1.05 (0.645 to 1.62) +5.12% (−35.5% to +61.8%)	0.980 (0.578 to 1.54) −2.03% (−42.2% to +54.3%)	1.00 (0.333 to 2.40) +0.0685% (−66.7% to +140%)	1385 (<min to >max)
	Food specialisation	Monoph.	0.582 (0.365 to 0.874) −41.8% (−63.5% to −12.6%)	0.842 (0.529 to 1.28) −15.8% (−47.1% to +27.6%)	1.33 (0.496 to 3.0) +32.6% (−50.4% to +200%)	<min (<min to >max)
		Oligoph.	0.435 (0.282 to 0.638) −56.5% (−71.8% to −36.2%)	1.02 (0.645 to 1.53) +2.12% (−35.5% to +52.6%)	2.59 (0.988 to 5.76) +159% (−1.23% to +476%)	1428 (894 to >max)
		Polyph.	0.702 (0.481 to 1.00) −29.8% (−51.9% to +0.423%)	1.37 (0.935 to 1.94) +36.8% (−6.51% to +93.8%)	2.82 (1.25 to 5.57) +182% (+24.6% to +457%)	812 (<min to 1543)
	Overwintering stage	Egg	0.589 (0.393 to 0.844) −41.1% (−60.7% to −15.6%)	0.843 (0.559 to 1.22) −15.7% (−44.1% to +21.9%)	1.29 (0.523 to 2.66) +28.6% (−47.7% to +166%)	2399 (<min to >max)
		Larva	0.758 (0.506 to 1.10) −24.2% (−49.4% to +9.97%)	1.22 (0.804 to 1.79) +22.4% (−19.6% to +79.2%)	2.10 (0.849 to 4.35) +110% (−15.1% to +335%)	832 (<min to >max)
		Pupa	0.584 (0.371 to 0.897) −41.6% (−62.9% to −10.3%)	1.61 (1.02 to 2.44) +61.4% (+1.96% to +144%)	4.82 (1.82 to 10.6) +382% (+82.3% to +959%)	825 (377 to 1289)
		Adult	0.805 (0.609 to 1.05) −19.5% (−39.1% to +5.29%)	0.804 (0.610 to 1.03) −19.6% (−39.0% to +3.18%)	0.827 (0.462 to 1.36) −17.3% (−53.8% to +35.9%)	<min (<min to >max)

*Note:* For three different elevations (lowest, median, highest), the change in the prediction from the first to the last study year is given, once as a factor and once as percentage change. Numbers are means and 95% credible intervals (CIs). The threshold elevation indicates the elevation at which the model terms for year and for the interactions between year and elevation cancel each other out, resulting in no predicted change across years for that respective elevation. Above and below the threshold, the model predicts yearly changes in opposite directions. Mean and 95% CIs are given for the thresholds. Threshold elevations that are outside of the elevational range studied here are simplified to “<min” (below lowest site, i.e., 193 m asl) and “>max” (above highest site, i.e., 2454 m asl). Red shading indicates decreases with 95% CIs not including no change, blue indicates increases with 95% CIs not including no change.

### 3.2 Differences Between Trait Groups

**FIGURE 3:**
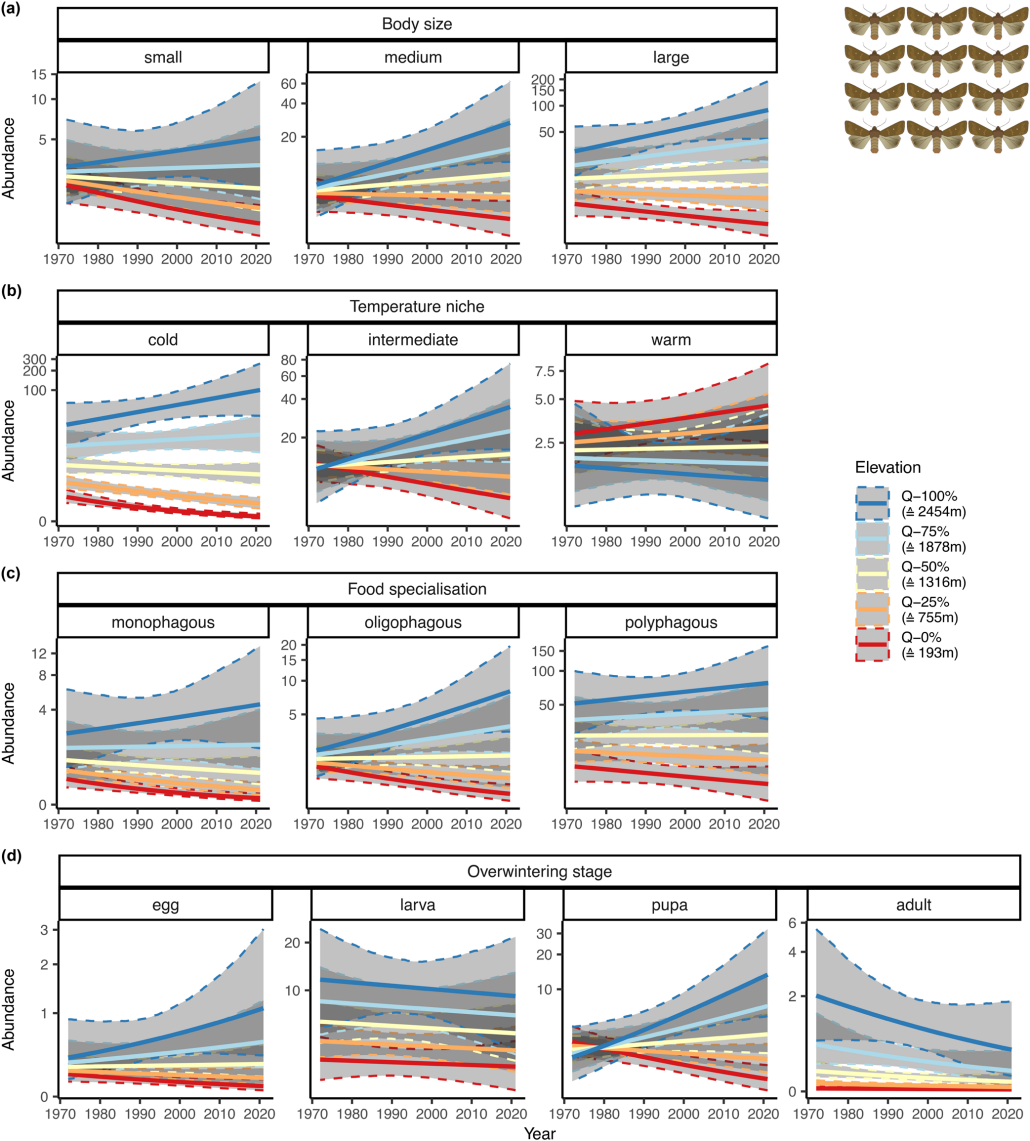
Change in moth abundance across the 50 study years (1972–2021) in relation to the elevation of the study site for different moth groups defined by species traits. Traits are (a) body size (mass), (b) temperature niche (mean temperature of species occurrence in Europe), (c) food specialisation (monophagous, oligophagous, polyphagous), and (d) overwintering stage (egg, larva, pupa, adult). For continuous traits (body size, temperature niche), groups were built along the 33% and 66% quantiles (one third of the recorded species in each group). Lines are point estimates from model predictions (conditional effects). Separate trend predictions are shown for different quantiles (0%, 25%, 50%, 75%, and 100%) of the elevational range. The corresponding elevations are indicated in the legend, for example, 193 m asl for the 0%‐quantile (minimum elevation covered in the data). Shaded areas show 95% credible intervals for the predictions. Note that the *y* axes are log transformed and differ among panels. Results for richness and biomass are shown in Figures S10 and S11. Detailed model results in Tables S4, S7, S10, and S13.

**FIGURE 4:**
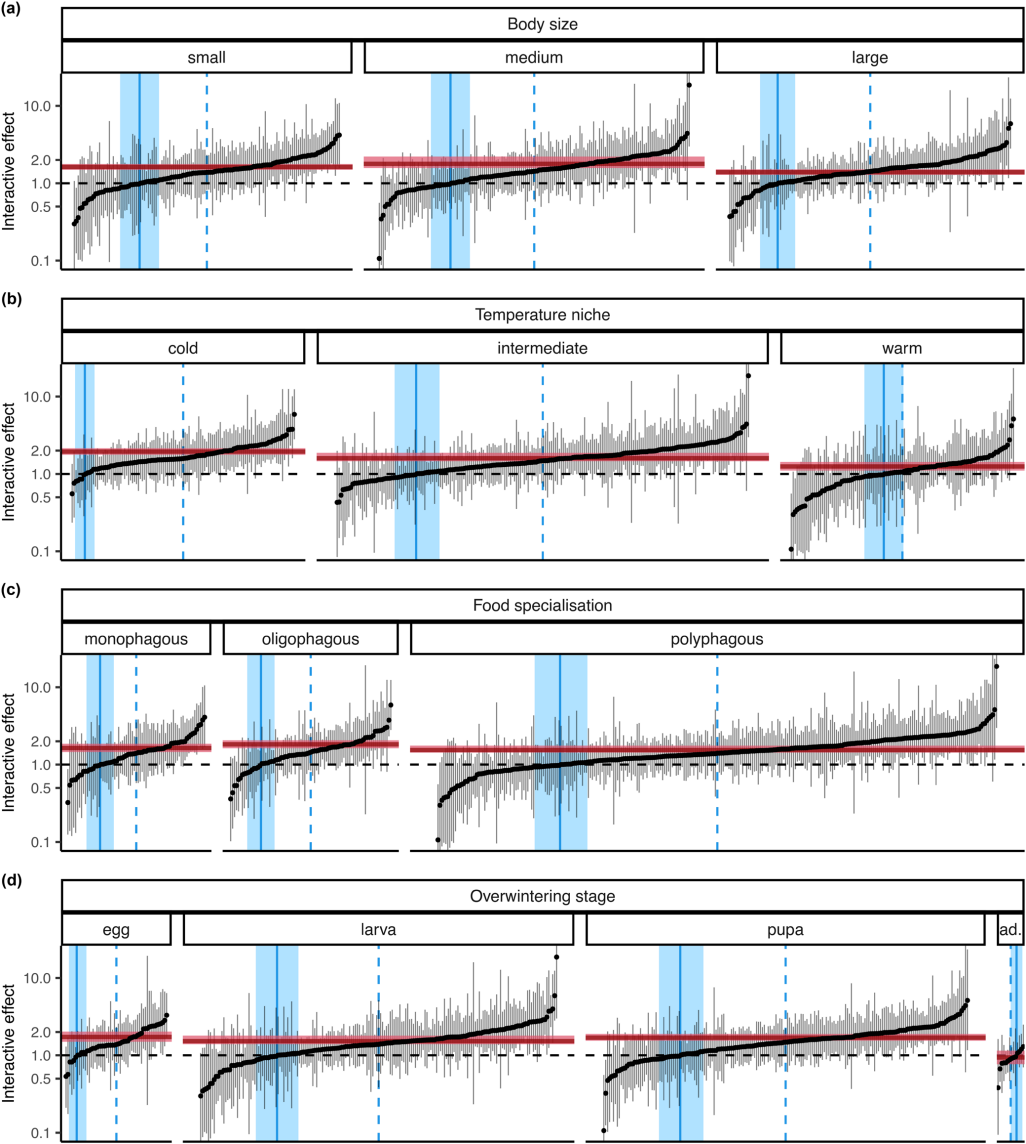
Single‐species model coefficients for the interactions between year and elevation, shown arranged along different trait values. Each point shows the mean of the posterior distribution of the interaction coefficient for one species, grey vertical lines show the 95% credible intervals (CIs). Each panel represents a categorical trait value for (a) body size, (b) temperature niche, (c) food specialisation and (d) overwintering stage and species within each panel are arranged along their mean values. The red horizontal lines show the mean coefficient across all species per trait value weighted by the total abundance of the species in the full dataset; the red shadings give the 95% CIs. The blue vertical dashed lines show the median of the number of species per panel, the vertical solid lines show where negative mean coefficient estimates change to positive mean coefficient estimates along with a bootstrap 95% confidence interval (*n* = 10,000). The *y* axis is scaled such that the numbers represent the change in the change factor across 10 years when moving 1000 m up the elevational gradient. For example, an estimate of 2 means that if abundance of a species was halved at 500 m asl over 10 years (i.e., factor of 0.5), it stayed constant at 1500 m asl (factor of 1). Only the 442 most frequently recorded species were included. ad. = adult.

The general 50‐year changes in moth community characteristics (total abundance, species richness, biomass) as well as the dependence of the changes on elevation varied among groups defined by different species traits (Figure 3, Table 1, Figures S10 and S11, Tables S3–S15). These community‐level patterns were also reflected in species‐level models (Figure 4).

For all trait groups defined by body size, there was evidence for decreases at low elevations and increases at high elevation in all community characteristics (Figure 3a, Table 1, Figures S10a and S11a, Tables S3–S6). The tendency towards decreases at low elevations was stronger for small species, while the tendency towards increases at high elevations was stronger for large species. In single‐species models, 71.7% (95% CI: 65.7% to 77.4%) of small species, 73.7% (95% CI: 68.8% to 78.8%) of medium species, and 76.9% (95% CI: 71.7% to 82.1%) of large species had a positive estimate of the interactive effect between year and elevation (Figure 4), indicating upward shifts of the focus of distribution of these species.

For trait groups defined by temperature niche, we found evidence for strongest changes for cold‐adapted species (Figure 3b, Table 1, Figures S10b and S11b, Tables S3 and S7–S9). For example, we estimated abundance decreases of cold‐adapted species at the lowest elevation to a factor of 0.144 (95% CI: 0.0689 to 0.268), which corresponds to a decrease of 85.6% (95% CI: −93.1% to −73.2%), and increases at the highest elevation were estimated to a factor of 4.61 (95% CI: 0.795 to 15.6), which corresponds to an increase of 361% (95% CI: −20.5% to +1465%). The change from low‐elevational decreases to high‐elevational increases was estimated to happen at about 1600 m asl, with decreases in community characteristics dominating at elevations below 1150 m asl with very high probability (Table 1, Table S3). Species adapted to intermediate temperatures also followed this pattern, but changes were estimated to be smaller (Table 1, Table S3). Warm‐adapted species, finally, showed no evidence for changes or partly even opposite patterns with some evidence for an increase in biomass at low elevations and a decrease in biomass at high elevations (Table S3, Figure S11b). In single‐species models, 87.3% (95% CI: 81.7% to 92.2%) of cold‐adapted species, 76.7% (95% CI: 72.2% to 81.1%) of species adapted to intermediate temperatures, and 56.1% (95% CI: 48.7% to 62.6%) of warm‐adapted species had a positive estimate of the interactive effect between year and elevation (Figure 4).

For trait groups defined by food specialisation, there was a dichotomous pattern of low‐elevational decreases and high‐elevational increases for all groups, but decreases at the lowest elevation were strongest for mono‐ and oligophagous species (Figure 3c, Table 1, Figures S10c and S11c, Tables S3, S10–S12). The interactive effect between year and elevation was strongest for oligophagous species, which was also reflected in the highest mean interactive effect estimates in the single‐species models for the oligophagous species (Figure 4). In these single‐species models, 71.9% (95% CI: 63.4% to 80.3%) of monophagous species, 75.8% (95% CI: 68.7% to 83.1%) of oligophagous, and 74.1% (95% CI: 70.0% to 77.7%) of polyphagous species had a positive estimate of the interactive effect between year and elevation (Figure 4).

We also found the tendency for dichotomous patterns along the elevational gradient for most trait groups defined by overwintering stage (Figure 3d, Table 1, Figures S10d and S11d, Tables S3, S13–S15). As an exception, the total abundance, richness, and biomass of species overwintering as adults decreased across the whole elevational gradient. The dichotomous pattern was strongest for species overwintering in the pupal stage (Table 1, Table S3). For example, abundance changes at the lowest elevation for this species group were estimated to a factor of 0.362 (95% CI: 0.174 to 0.672); that is, a decrease of 63.8% (95% CI: −82.6% to −32.8%), while for the highest elevation they were estimated to a factor of 8.33 (95% CI: 1.62–26.5), corresponding to an increase of 733% (95% CI: 61.7% to 2554%). The change from low‐elevational decreases to high‐elevational increases was estimated to happen at about 500–1000 m asl, with increases dominating with very high probability at elevations above 1300 m asl (richness, biomass) and 1700 m asl (abundance) (Table 1, Table S3). In single‐species models, 78.6% (95% CI: 67.9% to 86.8%) of species overwintering as egg, 73.2% (95% CI: 68.1% to 77.8%) of species overwintering as larva, 76.3% (95% CI: 72.0% to 81.0%) of species overwintering as pupa, and 37.6% (95% CI: 14.3% to 57.1%) of species overwintering as adult had a positive estimate of the interactive effect between year and elevation (Figure 4).

## 4 Discussion

We found that temporal trends of single species and moth community characteristics depended both on elevation and moth traits, showing the important role of local environmental conditions, anthropogenic pressures, and insect community composition for long‐term changes in insect communities. All community characteristics (i.e., total abundance, species richness and biomass) decreased at low elevation and increased at high elevation (question (i)). Also, the analyses of single species showed that a clear majority of species had more negative temporal trends at lower elevations. Thus, moth communities at low elevation impoverished and diminished, a pattern also found for butterflies in the Alps (Habel et al. 2023; Ulrich et al. 2025). The decline of the community characteristics at low elevations is also in line with recent findings on a general insect decline that mostly originate from low‐elevation sites (e.g., Hallmann et al. 2017; Seibold et al. 2019). With declines of total abundance, richness, and biomass at the lowest elevation estimated to 30%–40% over the 50 years, the magnitude of the decline reported here is lower than in some of the previous studies that found up to a 78% decline over just 10 years (Seibold et al. 2019), but is still considerable. At high elevation, the estimated increases corresponded to approximately a doubling in community characteristics, although subject to greater uncertainty. These strong changes in community characteristics may have profound effects on the functioning of both low‐ and high‐elevation ecosystems (Walton et al. 2020; Yazdanian et al. 2024). The dependence of temporal trends on elevation shows that trends in moth communities depend on local environmental conditions, their changes due to anthropogenic pressures, and differences in community composition based on adaptations to the specific local environmental conditions.

Our trait analyses confirmed that insect community composition is strongly related to long‐term temporal changes in insect communities, as temporal trends varied greatly among species groups defined by different traits (question (ii)). Declines at low elevation were most pronounced among cold‐adapted species, mono‐ and oligophagous species and species overwintering as pupa. Particularly for species adapted to cold and intermediate temperatures and for species overwintering as pupa, the declines were contrasted by increases at high elevation, indicating ongoing range shifts for these species (cf. Chen et al. 2009; Habel et al. 2023), which were also supported in single‐species models. These range shifts highlight the important role of climate change in driving insect community changes in general (Forister et al. 2021; Neff et al. 2022; Outhwaite et al. 2022; Soroye et al. 2020; Vidal et al. 2025) and moth community changes in particular (Fox 2013; Maes et al. 2024) during the last decades. Interestingly, while there were decreases of species adapted to cold and intermediate temperatures at low elevation, there was no clear evidence for increases of warm‐adapted species at low elevation, which would have been expected as a response to climate warming. As increases of warm‐adapted species at low elevations depend on latitudinal range shifts, which mean longer dispersal distances, the lack of an increase could indicate delayed immigration and hence a climatic debt of warm‐adapted species (Devictor et al. 2012). At the same time, the absence of clear patterns in community characteristics and single‐species range shifts of warm‐adapted species indicates that warm‐adapted species are more indifferent to climate change than species adapted to cold and intermediate temperatures, which have been shifting their elevational ranges considerably in recent decades.

The dependence of temporal trajectories on overwintering stage supports the role of climate change in driving the observed moth community changes. Our results match previous findings by Keret et al. (2020), who show elevational range shifts in Finland to be strongest among species overwintering in the pupal stages. Species overwintering as pupa spend most of the summer in the larval stage (cf. Neff et al. 2025b), which at least during early instars might be particularly sensitive to high temperatures (Ma et al. 2021). In the study region, late springs and summers have witnessed strong rises in average temperatures across the study period (Figure S12), which might explain the particularly strong range shifts in species overwintering as pupa. As previous studies repeatedly point out the important role of winter conditions in moth declines (Fox 2013; Hunter et al. 2014), the consequences of both winter and summer warming on different moth life stages need to be further studied to better understand the climate‐change driven risks different moth species are facing.

Generally, declines of moth numbers at low elevations could additionally be attributed to other anthropogenic pressures that are particularly prevalent in the lowlands, such as persisting high intensity of agricultural land use (Spörri et al. 2023) or increasing light pollution (Kyba et al. 2023), which are both known to negatively affect moth communities (Fox 2013; van Grunsven et al. 2020; Knop et al. 2017; Merckx and Van Dyck 2019). High agricultural land‐use intensity has also been related to decreases of plant species richness in agricultural habitats of the lowlands (Stehlik et al. 2007), indicating that anthropogenic pressures in lower elevations may have simultaneously impoverished plant and moth communities. The consequential lack of food plants would also explain the strong declines at low elevations among food‐specialised moths, which are in accordance with previous work (Franzén and Johannesson 2007; Roth et al. 2021; Valtonen et al. 2017; Wagner et al. 2021). Higher elevations are less affected by intensive land use or light pollution, which would explain the lack of decreases in community characteristics. At the same time, the increases that were indicated at high elevations for several trait groups, both in community‐level traits and for single species, indicate ongoing range shifts. Thus, the changes in moth community characteristics and the dependence of changes on species traits show the important role of climate change—resulting in range shifts of some species groups—but also support the role of other global change drivers such as land‐use intensification, urbanisation and light pollution in driving moth declines.

Our analyses based on a vast 50‐year dataset on moth communities showed considerable changes in the distribution and composition of moth communities in Switzerland. Climate change has led to elevational range shifts in many moth species, a process that has not come to a halt and will further affect moth communities in future decades. As the potential for elevational range shifts is limited, cold‐adapted species and species overwintering as pupa, which are the species that have been moving upwards the most, are especially vulnerable to extinction in the coming decades. Maintaining suitable high‐elevation habitats, which offer refugia for these species, will be key to sustaining diverse moth communities in the Alps. At the same time, other anthropogenic pressures such as land‐use change and intensification, urbanisation, and light pollution might have contributed to the impoverishment of moth communities in the lowlands. While the role of these different drivers needs to be evaluated in more detail, reducing these pressures can be key to halting ongoing declines in moth abundance and richness.

## Author Contributions

F.N., Y.C., G.L., E.R. collected and provided data; F.N. performed the analyses with support from F.K.‐N. and E.K.; F.N. wrote the first draft of the manuscript, with input from E.K.; all authors contributed substantially to the revisions.

## Peer Review

The peer review history for this article is available at https://www.webofscience.com/api/gateway/wos/peer‐review/10.1111/ele.70195.

## Supporting information


**Data S1:** ele70195‐sup‐0001‐supinfo.pdf.

## Data Availability

The moth records that support the findings of this study are openly available from the GBIF database https://doi.org/10.15468/dl.gcagva (GBIF.org 2024). A repository containing all codes and additional data necessary to reproduce the analyses is available from Zenodo https://doi.org/10.5281/zenodo.14506883 (Neff et al. 2025a).
