# Supplementary material for: Contrasting 50‐Year Trends of Moth Communities Depending on Elevation and Species Traits
Source: Ecol Lett. 2025 Aug 14;28(8):e70195. doi: 10.1111/ele.70195 (PMC12351669; doi:10.1111/ele.70195)
Supplement: Supplementary file 1 — Data S1: ele70195‐sup‐0001‐supinfo.pdf. [file ELE-28-0-s001.pdf]

SUPPORTING INFORMATION

Article Title: Contrasting 50-year trends of moth communities depending on elevation and species traits

Authors: Felix Neff, Yannick Chittaro, Fränzi Korner-Nievergelt, Glenn Litsios, Carlos Martínez-Núñez, Emmanuel Rey, Eva Knop

Corresponding Author: Felix Neff, Agroecology and Environment, Agroscope, Reckenholzstrasse 191, 8046 Zurich, Switzerland, Tel. 0041 58 463 54 20, mail@felixneff.ch

Appendix S1: Moth dataset details

The moth dataset contains macro-moth community data from light trapping, which was initiated and conducted by Dr. Ladislaus Rezbanyai-Reser (ZOBODAT 2014–2025) between 1972 and 2021 (no samples in 2019) (Fig. 1). L. Rezbanyai-Reser, curator at the Museum of Natural History in Lucerne for 40 years, was a naturalist and moth enthusiast with the aim to improve our knowledge of moths in various understudied regions of Switzerland. He did not have a predefined sampling strategy, because his basic aim was not to carry out long-term analyses, but to document the moth fauna of different sites, both in the lowlands and in the mountains. Thus, his approach was not strictly standardised but there was variation between sampling occasions, sites and years, which was influenced by e.g. time constraints, personal constraints or weather constraints. At the same time, L. Rezbanyai-Reser was always extremely rigorous in recording sampling information, which was systematically and clearly documented (number and type of traps used, type of bulb, often operating time of manual traps, etc.), all parameters that we considered in the analyses.

L. Rezbanyai-Reser used two different types of light traps (Figs S1–S2): On the one hand, he used fixed traps that were installed in a location and that then were active over a longer time (usually months), while being emptied daily by L. Rezbanyai-Reser and helpers. Two models of these fixed traps were in place, a more readily used trap model ("type 1", used in 154 site-year combinations) and a more rarely used trap model ("type 2", used in 10 site-year combination). On the other hand, he used manual traps that were installed only for single nights and were active for some hours (between 1 and 13 hours) within that night. Usually, L. Rezbanyai-Reser would go to sleep in his car next to the sampling site after the peak of activity, leaving the trap active to sample late arrivals. He would then get up shortly before sunrise to collect any specimens that had arrived. However, on some occasions, sampling duration was shortened due to personal or weather-related constraints (e.g. storms). The duration of manual sampling

was noted for most nights (70% of nights), so that we could account it for in our analyses. Sometimes, several traps were installed at a location (up to four pieces) (Fig. S3), of which counts were pooled across the installed traps. Also, lamps differed between different samplings or even traps in a sampling night (3 lamp types: 80W mercury mixed-light lamps, 150–160W mercury mixed-light lamps, 125W mercury vapour lamps) (Fig. S4). Trap type, number of traps and lamp type were constant across the different sampling nights per site within a sampling year.

Some sites at which manual traps were used were in close proximity (38m to 6.1km) and operated simultaneously in the same nights. These 94 sites, which can be grouped into 34 site groups, were still treated as separate sites, but their grouping was accounted for in the analyses. No data on trap failures of fixed traps across their active time periods was available, which is why stretches of 10 and more nights in a row without any records were interpreted as trap failures and excluded from analyses (1,614 nights). Basically, sampled nights spread across the entire year. For fixed traps, 194 nights per location were on average sampled per year (range: 5–362 nights), with July 24 being the mean sampling date (range of mean: February 3 to August 28). For manual traps, 8 nights per location were on average sampled per year (range: 1–71 nights), with July 16 being the mean sampling date (range of mean: February 3 to November 11) (Fig. S5).

The moths captured in the fixed traps were directly killed with a sampling fluid (mostly chloroform). The manual traps also had a collecting container with an interception panel to capture the majority of moths directly. Individuals resting on the white sheet were added to this container manually as they arrived, so that no individual could be counted more than once. Identification, counting and preparation of specimens were always carried out in the lab, usually the following day for manual traps and weekly for fixed traps, from which the regional collaborators sent the various daily bags of captured specimens once a week. The abundance of each

species for each night and each trap was manually recorded in handwritten notebooks (all of which are kept at the Lucerne Museum) and then digitised by collaborators at info fauna or by L. Rezbanyai-Reser. These data have then been integrated into the national database info fauna.

L. Rezbanyai-Reser created extensive reference collections for all sites. In addition, almost all samplings sites have been the subject of publications presenting the overall results (lists of species, information on trapping, etc.). A list of his more than 400 (regional) publications can be found in Rezbanyai-Reser (2018). Depending on the location of the study sites, the reference collections have been deposited at the Lucerne Museum, the Glarus Museum, the Lugano Museum or the Jura Museum. For each reported species, at least one specimen was prepared in dry condition by either L.Rezbanyai-Reser or his helper Erwin Schäffer and carefully labelled. Depending on the difficulty of identification and the possible existence of cryptic diversity, more specimens per species were included in these collections. When the examination of the genitalia was necessary for a reliable identification (e.g. numerous *Eupitecia* spp.), all specimens were dissected, with a large number (sometimes all) being prepared.

When the taxonomic status of a taxon was revised following sampling at a given site, L. Rezbanyai-Reser would systematically review his previous identifications and announce any necessary adaptations to the national fauna information database. Over time, and particularly im preparation for the publication of the book on the Noctuidae of Switzerland (Wymann *et al.* 2015), of which he is a co-author, he has conducted critical checks of distribution maps, elevational ranges and phenologies, resulting in further corrections to the data. The dataset used for our analyses has therefore undergone multiple and rigorous quality checks.

The taxonomy and systematics used here follow those on the Lepiforum e.V. website [status 2023], undoubtedly the most comprehensive and up-to-date reference for the Lepidoptera fauna of Central Europe.

Supplementary References

Rezbanyai-Reser, L. (2018). Vollständige Literaturliste "Rézbányai" bzw. "Rezbanyai-Reser" 1968-2018 (Lepidoptera und andere Insekten). *Lepidopterol. Mitteilungen Aus Luzern*, 22, 1–56.

Wymann, H.-P., Rezbanyai-Reser, L. & Hächler, M. (2015). *Die Eulenfalter der Schweiz: Lepidoptera Noctuidae, Pantheidae, Nolidae*. Fauna Helvetica. CSCF & SEG, Neuchâtel, Switzerland.

ZOBODAT. (2014–2025). *Dr. Ladislaus Rezbanyai-Reser (auch Rezbanyai)*. Available at: https://www.zobodat.at/personen.php?id=1004. Last accessed 20 January 2025.

Appendix S2: Sensitivity analyses

We performed several sensitivity analyses to check for confounding effects of dataset specifics. First, sampling characteristics such as trap type varied among sampling sites and years (Fig. S2). To analyse the role of these sampling characteristics, we fitted models to different subsets of the full dataset: On the one hand, we separated the dataset by trap type and ran models for fixed traps only as well as for manual traps only. On the other hand, we excluded data from manual traps for which we did not have data on sampling duration and ran the models on this subset. The interactive effect with (tendency for) decreases at low elevations and (tendency for) increases at high elevations could be confirmed in most analyses including only subsets of the data (Fig. S13, Tables S16–S18), even though statistical uncertainty of single estimates was large. As an exception, analyses based on the dataset including manual traps only indicated a general decrease in abundance irrespective of elevation (Fig. S13, Table S17).

Second, sampling sites changed across the years and no site was sampled across the full study period (Fig. 1c). To rule out that a change in the choice of sampling sites was driving the results, we fitted additional single-site models. We selected sites that were sampled in at least four years and had 10 or more sampling nights (73 out of 171 sites). For each of these sites, we fitted the three community-level models (total abundance, species richness, biomass). The models had the same structure as the above models but only included factors to correct for sampling characteristics (e.g. trap type) if these were relevant for the respective subset of the data. We also excluded elevation and the interaction between elevation and year from the single-site models, as they are not meaningful at the site level. We reduced the random structure to a random effect for the year. From each single-site model, we then extracted the coefficient estimate for year. Finally, we related these estimates to the elevations of the sites to test whether the interactive effect of year and elevation was reflected in the temporal changes per site. If

site selection was not driving the observed results, the positive interactive effect between elevation and year, which we found for the full dataset, should be reflected in these single-site models with more positive coefficients for the year at higher-elevation sites. Indeed, there was a positive relation between the coefficient for the year and the elevation of the site (Fig. S14), showing that temporal changes at sites of higher elevation were more positive. Thus, the site selection was not driving the patterns observed in the full dataset.

Table S1 Overview of all moth species recorded, their according categorical traits (body size, temperature niche, specialisation, overwintering stage), and the total count (i.e., summed abundance) in the complete dataset**.**

Family	Species	Body size	Temp. niche	Specialisation	Overw. stage	Total count
Brahmaeidae	*Lemonia taraxaci*	large	intermediate	oligophagous	egg	1338
Cossidae	*Cossus cossus*	large	intermediate	polyphagous	larva	189
Cossidae	*Phragmataecia castaneae*	large	warm	monophagous	larva	2095
Cossidae	*Zeuzera pyrina*	large	warm	polyphagous	larva	552
Drepanidae	*Achlya flavicornis*	large	cold	monophagous	pupa	1746
Drepanidae	*Cilix glaucata*	small	warm	oligophagous	pupa	110
Drepanidae	*Cymatophorina diluta*	medium	warm	monophagous	egg	817
Drepanidae	*Drepana curvatula*	medium	intermediate	oligophagous	pupa	196
Drepanidae	*Drepana falcataria*	medium	cold	oligophagous	pupa	2078
Drepanidae	*Falcaria lacertinaria*	medium	cold	oligophagous	pupa	202
Drepanidae	*Habrosyne pyritoides*	large	warm	monophagous	pupa	7265
Drepanidae	*Ochropacha duplaris*	medium	cold	polyphagous	pupa	5061
Drepanidae	*Polyploca ridens*	medium	warm	monophagous	pupa	836
Drepanidae	*Sabra harpagula*	medium	intermediate	polyphagous	pupa	1820
Drepanidae	*Tethea ocularis*	large	warm	monophagous	pupa	95
Drepanidae	*Tethea or*	large	intermediate	monophagous	pupa	1377
Drepanidae	*Tetheella fluctuosa*	large	cold	monophagous	pupa	46
Drepanidae	*Thyatira batis*	large	intermediate	monophagous	pupa	3764
Drepanidae	*Watsonalla binaria*	medium	warm	monophagous	pupa	1683
Drepanidae	*Watsonalla cultraria*	medium	warm	monophagous	pupa	5458
Drepanidae	*Watsonalla uncinula*	medium	warm	monophagous	pupa	64
Endromidae	*Endromis versicolora*	large	cold	polyphagous	pupa	223
Erebidae	*Arctia caja*	large	intermediate	polyphagous	larva	1619
Erebidae	*Arctia flavia*	large	cold	polyphagous	larva	17
Erebidae	*Arctia matronula*	large	cold	polyphagous	larva	59
Erebidae	*Arctia testudinaria*	medium	warm	polyphagous	larva	411
Erebidae	*Arctia villica*	large	warm	polyphagous	larva	1081
Erebidae	*Arctornis l-nigrum*	medium	warm	polyphagous	larva	765
Erebidae	*Atolmis rubricollis*	medium	intermediate	polyphagous	pupa	2897
Erebidae	*Autophila dilucida*	medium	warm	oligophagous	adult	4
Erebidae	*Callimorpha dominula*	large	intermediate	polyphagous	larva	909
Erebidae	*Calliteara pudibunda*	large	warm	polyphagous	pupa	6091
Erebidae	*Calyptra thalictri*	medium	cold	monophagous	larva	127
Erebidae	*Catephia alchymista*	medium	warm	monophagous	pupa	80
Erebidae	*Catocala coniuncta*	large	warm	monophagous	egg	1
Erebidae	*Catocala dilecta*	large	warm	monophagous	egg	2
Erebidae	*Catocala electa*	large	warm	oligophagous	egg	33
Erebidae	*Catocala elocata*	large	warm	monophagous	egg	18
Erebidae	*Catocala fraxini*	large	cold	polyphagous	egg	188
Erebidae	*Catocala fulminea*	large	intermediate	monophagous	egg	4
Erebidae	*Catocala nupta*	large	warm	polyphagous	egg	168
Erebidae	*Catocala nymphaea*	large	warm	monophagous	egg	44
Erebidae	*Catocala nymphagoga*	medium	warm	monophagous	egg	1
Erebidae	*Catocala promissa*	large	warm	monophagous	egg	33
Erebidae	*Catocala puerpera*	large	warm	oligophagous	egg	8
Erebidae	*Catocala sponsa*	large	intermediate	monophagous	egg	21
Erebidae	*Chelis simplonica*	medium	cold	polyphagous	larva	3
Erebidae	*Colobochyla salicalis*	medium	intermediate	oligophagous	pupa	506
Erebidae	*Coscinia cribraria*	medium	intermediate	polyphagous	larva	1232
Erebidae	*Cybosia mesomella*	medium	intermediate	polyphagous	larva	255
Erebidae	*Diacrisia purpurata*	large	intermediate	polyphagous	larva	126
Erebidae	*Diacrisia sannio*	medium	intermediate	polyphagous	larva	2569
Erebidae	*Diaphora mendica*	medium	intermediate	polyphagous	pupa	2677
Erebidae	*Diaphora sordida*	small	cold	polyphagous	pupa	4
Erebidae	*Dysgonia algira*	medium	warm	polyphagous	pupa	1536
Erebidae	*Eilema caniola*	medium	warm	polyphagous	larva	4143
Erebidae	*Eilema cereola*	medium	cold	polyphagous	larva	235
Erebidae	*Eilema complana*	medium	intermediate	polyphagous	larva	23799
Erebidae	*Eilema depressa*	medium	intermediate	polyphagous	larva	50470
Erebidae	*Eilema griseola*	medium	intermediate	polyphagous	larva	4166
Erebidae	*Eilema lurideola*	medium	intermediate	polyphagous	larva	22667
Erebidae	*Eilema lutarella*	medium	cold	polyphagous	larva	270
Erebidae	*Eilema palliatella*	medium	intermediate	polyphagous	larva	186
Erebidae	*Eilema pseudocomplana*	medium	warm	polyphagous	larva	305
Erebidae	*Eilema pygmaeola*	medium	warm	polyphagous	larva	994
Erebidae	*Eilema sororcula*	medium	warm	polyphagous	pupa	12858
Erebidae	*Epatolmis luctifera*	medium	warm	polyphagous	pupa	50
Erebidae	*Eublemma ostrina*	small	warm	monophagous	pupa	19
Erebidae	*Eublemma parva*	small	warm	oligophagous	pupa	46
Erebidae	*Eublemma polygramma*	small	warm	NA	NA	24
Erebidae	*Eublemma purpurina*	small	warm	oligophagous	larva	30
Erebidae	*Euplagia quadripunctaria*	large	warm	polyphagous	larva	1396
Erebidae	*Euproctis chrysorrhoea*	medium	warm	polyphagous	larva	1853
Erebidae	*Grammodes stolida*	medium	warm	polyphagous	pupa	1

Erebidae	*Gynaephora fascelina*	medium	cold	polyphagous	larva	1321
Erebidae	*Herminia grisealis*	medium	intermediate	polyphagous	pupa	4335
Erebidae	*Herminia tarsicrinalis*	medium	intermediate	polyphagous	larva	7467
Erebidae	*Herminia tarsipennalis*	medium	intermediate	polyphagous	larva	2459
Erebidae	*Herminia tenuialis*	small	warm	polyphagous	pupa	1672
Erebidae	*Hypena crassalis*	medium	cold	monophagous	pupa	754
Erebidae	*Hypena lividalis*	small	warm	oligophagous	adult	1
Erebidae	*Hypena obesalis*	medium	intermediate	polyphagous	adult	857
Erebidae	*Hypena obsitalis*	medium	warm	oligophagous	adult	18
Erebidae	*Hypena proboscidalis*	medium	intermediate	polyphagous	larva	8608
Erebidae	*Hypena rostralis*	medium	intermediate	monophagous	adult	450
Erebidae	*Hypenodes humidalis*	small	cold	polyphagous	larva	52
Erebidae	*Hyphantria cunea*	medium	warm	polyphagous	pupa	1
Erebidae	*Idia calvaria*	medium	warm	polyphagous	larva	154
Erebidae	*Laspeyria flexula*	medium	intermediate	polyphagous	larva	4559
Erebidae	*Leucoma salicis*	large	cold	oligophagous	larva	135
Erebidae	*Lithosia quadra*	medium	warm	polyphagous	larva	6088
Erebidae	*Lygephila craccae*	medium	warm	oligophagous	egg	2153
Erebidae	*Lygephila lusoria*	large	intermediate	oligophagous	larva	25
Erebidae	*Lygephila pastinum*	medium	intermediate	oligophagous	larva	342
Erebidae	*Lygephila viciae*	medium	cold	oligophagous	pupa	299
Erebidae	*Lymantria dispar*	large	warm	polyphagous	egg	1345
Erebidae	*Lymantria monacha*	large	intermediate	polyphagous	egg	7370
Erebidae	*Macrochilo cribrumalis*	medium	intermediate	polyphagous	larva	18
Erebidae	*Metachrostis dardouini*	small	warm	monophagous	pupa	33
Erebidae	*Miltochrista miniata*	medium	intermediate	polyphagous	larva	13805
Erebidae	*Minucia lunaris*	large	warm	monophagous	pupa	134
Erebidae	*Nudaria mundana*	small	intermediate	polyphagous	larva	1022
Erebidae	*Ocneria rubea*	medium	warm	polyphagous	larva	148
Erebidae	*Ocnogyna parasita*	medium	intermediate	polyphagous	pupa	32
Erebidae	*Orgyia antiqua*	medium	intermediate	polyphagous	egg	79
Erebidae	*Orgyia recens*	medium	intermediate	polyphagous	larva	1
Erebidae	*Paidia rica*	medium	warm	polyphagous	larva	16
Erebidae	*Paracolax tristalis*	medium	warm	polyphagous	larva	13119
Erebidae	*Parascotia fuliginaria*	small	intermediate	polyphagous	larva	248
Erebidae	*Pechipogo strigilata*	medium	cold	polyphagous	larva	1593
Erebidae	*Pelosia muscerda*	medium	intermediate	polyphagous	larva	7356
Erebidae	*Pelosia obtusa*	small	intermediate	NA	larva	16
Erebidae	*Phragmatobia fuliginosa*	medium	intermediate	polyphagous	larva	6383
Erebidae	*Phytometra viridaria*	small	warm	monophagous	pupa	403
Erebidae	*Polypogon gryphalis*	medium	intermediate	polyphagous	larva	280
Erebidae	*Polypogon plumigeralis*	medium	warm	polyphagous	larva	56
Erebidae	*Polypogon tentacularia*	medium	cold	polyphagous	larva	125
Erebidae	*Rivula sericealis*	small	intermediate	polyphagous	larva	12847
Erebidae	*Schrankia costaestrigalis*	small	intermediate	polyphagous	larva	406
Erebidae	*Schrankia taenialis*	small	warm	polyphagous	pupa	23
Erebidae	*Scoliopteryx libatrix*	medium	intermediate	polyphagous	adult	635
Erebidae	*Setina irrorella*	medium	cold	polyphagous	larva	1254
Erebidae	*Sphrageidus similis*	medium	cold	polyphagous	larva	805
Erebidae	*Spilarctia lutea*	medium	intermediate	polyphagous	pupa	7163
Erebidae	*Spilosoma lubricipeda*	medium	intermediate	polyphagous	pupa	8149
Erebidae	*Spilosoma urticae*	medium	intermediate	polyphagous	pupa	146
Erebidae	*Thumatha senex*	small	intermediate	polyphagous	larva	648
Erebidae	*Trisateles emortualis*	medium	intermediate	polyphagous	pupa	2208
Erebidae	*Tyria jacobaeae*	medium	warm	oligophagous	pupa	27
Erebidae	*Watsonarctia deserta*	medium	warm	oligophagous	pupa	1
Erebidae	*Zanclognatha lunalis*	medium	warm	polyphagous	larva	5633
Erebidae	*Zanclognatha zelleralis*	medium	warm	polyphagous	larva	273
Euteliidae	*Eutelia adulatrix*	medium	warm	polyphagous	pupa	126
Geometridae	*Abraxas grossulariata*	medium	warm	polyphagous	larva	466
Geometridae	*Abraxas sylvata*	medium	intermediate	polyphagous	pupa	4366
Geometridae	*Acasis appensata*	small	cold	monophagous	pupa	1
Geometridae	*Acasis viretata*	small	intermediate	polyphagous	pupa	413
Geometridae	*Adactylotis contaminaria*	small	warm	monophagous	NA	59
Geometridae	*Aethalura punctulata*	small	intermediate	oligophagous	pupa	1058
Geometridae	*Agriopis aurantiaria*	medium	intermediate	polyphagous	egg	1117
Geometridae	*Agriopis bajaria*	small	warm	polyphagous	egg	36
Geometridae	*Agriopis leucophaearia*	small	warm	polyphagous	pupa	349
Geometridae	*Agriopis marginaria*	medium	warm	polyphagous	pupa	2034
Geometridae	*Alcis deversata*	medium	cold	polyphagous	larva	300
Geometridae	*Alcis jubata*	small	cold	monophagous	larva	25
Geometridae	*Alcis repandata*	medium	intermediate	polyphagous	larva	44342
Geometridae	*Aleucis distinctata*	small	warm	monophagous	pupa	242
Geometridae	*Alsophila aceraria*	small	warm	polyphagous	larva	43
Geometridae	*Alsophila aescularia*	small	warm	polyphagous	pupa	4474
Geometridae	*Angerona prunaria*	medium	intermediate	polyphagous	larva	2594
Geometridae	*Anticlea derivata*	small	intermediate	monophagous	pupa	261
Geometridae	*Anticollix sparsata*	small	cold	monophagous	pupa	104

Geometridae	*Apeira syringaria*	medium	intermediate	polyphagous	larva	429
Geometridae	*Aplocera plagiata*	medium	intermediate	monophagous	larva	358
Geometridae	*Aplocera praeformata*	medium	cold	monophagous	larva	8731
Geometridae	*Apocheima hispidaria*	medium	warm	polyphagous	pupa	83
Geometridae	*Arichanna melanaria*	medium	cold	monophagous	larva	14
Geometridae	*Ascotis selenaria*	medium	warm	polyphagous	pupa	307
Geometridae	*Aspitates gilvaria*	small	warm	polyphagous	larva	1
Geometridae	*Asthena albulata*	small	intermediate	polyphagous	pupa	2103
Geometridae	*Asthena anseraria*	small	intermediate	monophagous	pupa	161
Geometridae	*Biston betularia*	medium	intermediate	polyphagous	pupa	7720
Geometridae	*Biston strataria*	medium	warm	polyphagous	pupa	2561
Geometridae	*Bupalus piniaria*	small	intermediate	oligophagous	pupa	1284
Geometridae	*Cabera exanthemata*	small	cold	polyphagous	pupa	6131
Geometridae	*Cabera pusaria*	small	intermediate	polyphagous	pupa	16479
Geometridae	*Campaea margaritaria*	medium	intermediate	polyphagous	larva	24197
Geometridae	*Camptogramma bilineata*	small	warm	polyphagous	larva	3004
Geometridae	*Camptogramma scripturata*	small	cold	polyphagous	larva	56
Geometridae	*Carsia sororiata*	small	cold	monophagous	egg	9
Geometridae	*Cataclysme riguata*	small	warm	oligophagous	pupa	3191
Geometridae	*Catarhoe cuculata*	small	intermediate	monophagous	pupa	1189
Geometridae	*Catarhoe rubidata*	small	warm	monophagous	pupa	559
Geometridae	*Cepphis advenaria*	small	intermediate	polyphagous	pupa	534
Geometridae	*Charissa ambiguata*	small	intermediate	polyphagous	larva	737
Geometridae	*Charissa glaucinaria*	medium	cold	polyphagous	larva	2327
Geometridae	*Charissa italohelveticus*	small	cold	NA	larva	137
Geometridae	*Charissa obscurata*	small	intermediate	polyphagous	larva	395
Geometridae	*Charissa pullata*	medium	cold	polyphagous	larva	289
Geometridae	*Charissa variegata*	small	warm	polyphagous	larva	236
Geometridae	*Chesias legatella*	medium	warm	oligophagous	egg	110
Geometridae	*Chesias rufata*	small	warm	oligophagous	pupa	9
Geometridae	*Chiasmia clathrata*	small	intermediate	oligophagous	pupa	15542
Geometridae	*Chlorissa cloraria*	small	warm	polyphagous	pupa	48
Geometridae	*Chlorissa viridata*	small	intermediate	polyphagous	pupa	29
Geometridae	*Chloroclysta miata*	medium	cold	polyphagous	adult	2300
Geometridae	*Chloroclysta siterata*	small	intermediate	polyphagous	adult	9779
Geometridae	*Chloroclystis v-ata*	small	warm	polyphagous	pupa	12398
Geometridae	*Cidaria fulvata*	small	intermediate	monophagous	egg	1722
Geometridae	*Cleora cinctaria*	medium	cold	polyphagous	pupa	870
Geometridae	*Cleorodes lichenaria*	small	warm	polyphagous	larva	110
Geometridae	*Coenotephria ablutaria*	small	warm	monophagous	larva	405
Geometridae	*Coenotephria salicata*	small	intermediate	monophagous	larva	4030
Geometridae	*Coenotephria tophaceata*	small	intermediate	monophagous	larva	476
Geometridae	*Colostygia aptata*	small	cold	monophagous	larva	855
Geometridae	*Colostygia aqueata*	small	cold	polyphagous	pupa	498
Geometridae	*Colostygia kollariaria*	medium	cold	monophagous	larva	119
Geometridae	*Colostygia laetaria*	small	cold	monophagous	larva	65
Geometridae	*Colostygia olivata*	small	intermediate	monophagous	larva	2097
Geometridae	*Colostygia pectinataria*	small	intermediate	polyphagous	larva	3312
Geometridae	*Colostygia puengeleri*	medium	cold	monophagous	NA	247
Geometridae	*Colostygia turbata*	small	cold	monophagous	larva	2649
Geometridae	*Colotois pennaria*	medium	intermediate	polyphagous	egg	3555
Geometridae	*Comibaena bajularia*	small	warm	monophagous	larva	335
Geometridae	*Cosmorhoe ocellata*	small	intermediate	monophagous	larva	4348
Geometridae	*Costaconvexa polygrammata*	small	warm	monophagous	pupa	2
Geometridae	*Crocallis elinguaria*	medium	intermediate	polyphagous	larva	2181
Geometridae	*Crocallis tusciaria*	medium	warm	polyphagous	egg	77
Geometridae	*Cyclophora albiocellaria*	small	warm	monophagous	pupa	1
Geometridae	*Cyclophora albipunctata*	small	cold	monophagous	pupa	65
Geometridae	*Cyclophora annularia*	small	warm	monophagous	pupa	2903
Geometridae	*Cyclophora linearia*	small	warm	polyphagous	pupa	10387
Geometridae	*Cyclophora pendularia*	small	intermediate	monophagous	pupa	22
Geometridae	*Cyclophora porata*	small	warm	monophagous	pupa	6
Geometridae	*Cyclophora punctaria*	small	warm	monophagous	pupa	1689
Geometridae	*Cyclophora puppillaria*	small	warm	polyphagous	pupa	245
Geometridae	*Cyclophora quercimontaria*	small	intermediate	monophagous	pupa	31
Geometridae	*Cyclophora ruficiliaria*	small	warm	monophagous	pupa	351
Geometridae	*Cyclophora suppunctaria*	small	warm	monophagous	pupa	1
Geometridae	*Deileptenia ribeata*	medium	intermediate	polyphagous	larva	4531
Geometridae	*Dysstroma citrata*	small	cold	polyphagous	egg	17765
Geometridae	*Dysstroma truncata*	small	cold	polyphagous	larva	11100
Geometridae	*Earophila badiata*	small	intermediate	monophagous	pupa	461
Geometridae	*Ecliptopera capitata*	small	cold	monophagous	pupa	1620
Geometridae	*Ecliptopera silaceata*	small	cold	polyphagous	pupa	3274
Geometridae	*Ectropis crepuscularia*	medium	intermediate	polyphagous	pupa	11280
Geometridae	*Electrophaes corylata*	small	cold	polyphagous	pupa	1305
Geometridae	*Elophos caelibaria*	small	cold	polyphagous	larva	118
Geometridae	*Elophos operaria*	medium	cold	polyphagous	larva	112
Geometridae	*Ematurga atomaria*	small	intermediate	polyphagous	pupa	146

Geometridae	*Emmiltis pygmaearia*	small	warm	NA	larva	6
Geometridae	*Ennomos alniaria*	medium	intermediate	polyphagous	egg	53
Geometridae	*Ennomos erosaria*	medium	intermediate	polyphagous	egg	67
Geometridae	*Ennomos fuscantaria*	medium	intermediate	polyphagous	egg	295
Geometridae	*Ennomos quercaria*	medium	warm	monophagous	egg	5
Geometridae	*Ennomos quercinaria*	medium	warm	polyphagous	egg	2705
Geometridae	*Entephria caesiata*	medium	cold	polyphagous	larva	26147
Geometridae	*Entephria cyanata*	small	cold	monophagous	larva	296
Geometridae	*Entephria flavata*	small	cold	NA	larva	24
Geometridae	*Entephria flavicinctata*	medium	cold	polyphagous	larva	337
Geometridae	*Entephria infidaria*	small	cold	polyphagous	larva	362
Geometridae	*Entephria nobiliaria*	small	cold	monophagous	larva	326
Geometridae	*Epilobophora sabinata*	small	cold	monophagous	larva	924
Geometridae	*Epione repandaria*	small	cold	polyphagous	egg	686
Geometridae	*Epione vespertaria*	small	cold	polyphagous	egg	129
Geometridae	*Epirrhoe alternata*	small	intermediate	monophagous	pupa	8934
Geometridae	*Epirrhoe galiata*	small	warm	monophagous	pupa	2769
Geometridae	*Epirrhoe molluginata*	small	intermediate	monophagous	pupa	3612
Geometridae	*Epirrhoe rivata*	small	intermediate	monophagous	pupa	2076
Geometridae	*Epirrhoe tristata*	small	cold	monophagous	pupa	249
Geometridae	*Epirrita autumnata*	small	cold	polyphagous	egg	4426
Geometridae	*Epirrita christyi*	small	intermediate	polyphagous	egg	10066
Geometridae	*Epirrita dilutata*	medium	intermediate	polyphagous	egg	1695
Geometridae	*Erannis defoliaria*	medium	intermediate	polyphagous	egg	2008
Geometridae	*Euchoeca nebulata*	small	intermediate	oligophagous	pupa	3756
Geometridae	*Eulithis mellinata*	small	cold	monophagous	egg	16
Geometridae	*Eulithis populata*	medium	cold	polyphagous	egg	15626
Geometridae	*Eulithis prunata*	medium	cold	monophagous	egg	298
Geometridae	*Eulithis testata*	small	cold	polyphagous	egg	177
Geometridae	*Euphyia biangulata*	small	warm	oligophagous	pupa	20
Geometridae	*Euphyia frustata*	small	warm	polyphagous	larva/pupa	3346
Geometridae	*Euphyia unangulata*	small	cold	monophagous	pupa	33
Geometridae	*Eupithecia abbreviata*	small	warm	monophagous	pupa	1996
Geometridae	*Eupithecia abietaria*	small	cold	oligophagous	pupa	524
Geometridae	*Eupithecia absinthiata*	small	cold	polyphagous	pupa	305
Geometridae	*Eupithecia actaeata*	small	cold	oligophagous	pupa	39
Geometridae	*Eupithecia analoga*	small	cold	monophagous	pupa	55
Geometridae	*Eupithecia assimilata*	small	cold	monophagous	pupa	389
Geometridae	*Eupithecia carpophagata*	small	warm	monophagous	pupa	8
Geometridae	*Eupithecia cauchiata*	small	cold	monophagous	pupa	61
Geometridae	*Eupithecia centaureata*	small	warm	polyphagous	pupa	58
Geometridae	*Eupithecia conterminata*	small	cold	monophagous	pupa	6
Geometridae	*Eupithecia cretaceata*	small	cold	monophagous	pupa	75
Geometridae	*Eupithecia denotata*	small	cold	monophagous	pupa	198
Geometridae	*Eupithecia denticulata*	small	warm	monophagous	pupa	25
Geometridae	*Eupithecia distinctaria*	small	warm	oligophagous	pupa	624
Geometridae	*Eupithecia dodoneata*	small	warm	polyphagous	pupa	1643
Geometridae	*Eupithecia egenaria*	small	intermediate	monophagous	pupa	926
Geometridae	*Eupithecia ericeata*	small	warm	polyphagous	egg	363
Geometridae	*Eupithecia exiguata*	small	intermediate	polyphagous	pupa	542
Geometridae	*Eupithecia expallidata*	small	intermediate	oligophagous	pupa	103
Geometridae	*Eupithecia extraversaria*	small	intermediate	oligophagous	pupa	65
Geometridae	*Eupithecia gemellata*	small	warm	monophagous	pupa	59
Geometridae	*Eupithecia graphata*	small	intermediate	oligophagous	pupa	13
Geometridae	*Eupithecia gueneata*	small	warm	oligophagous	pupa	3
Geometridae	*Eupithecia haworthiata*	small	warm	monophagous	pupa	3837
Geometridae	*Eupithecia icterata*	small	intermediate	oligophagous	pupa	6154
Geometridae	*Eupithecia immundata*	small	cold	monophagous	pupa	1
Geometridae	*Eupithecia impurata*	small	cold	monophagous	pupa	479
Geometridae	*Eupithecia indigata*	small	cold	monophagous	pupa	264
Geometridae	*Eupithecia innotata*	small	intermediate	polyphagous	pupa	154
Geometridae	*Eupithecia insigniata*	small	warm	oligophagous	pupa	8
Geometridae	*Eupithecia intricata*	small	cold	monophagous	pupa	188
Geometridae	*Eupithecia inturbata*	small	intermediate	monophagous	egg	221
Geometridae	*Eupithecia irriguata*	small	warm	monophagous	pupa	17
Geometridae	*Eupithecia lanceata*	small	cold	monophagous	pupa	391
Geometridae	*Eupithecia laquaearia*	small	warm	oligophagous	pupa	18
Geometridae	*Eupithecia lariciata*	small	intermediate	monophagous	pupa	10004
Geometridae	*Eupithecia linariata*	small	intermediate	monophagous	pupa	130
Geometridae	*Eupithecia nanata*	small	intermediate	monophagous	pupa	747
Geometridae	*Eupithecia ochridata*	small	cold	polyphagous	pupa	325
Geometridae	*Eupithecia orphnata*	small	cold	polyphagous	pupa	61
Geometridae	*Eupithecia pernotata*	small	cold	polyphagous	pupa	43
Geometridae	*Eupithecia pimpinellata*	small	cold	monophagous	pupa	186
Geometridae	*Eupithecia plumbeolata*	small	cold	oligophagous	pupa	352
Geometridae	*Eupithecia pusillata*	small	cold	monophagous	egg	3991
Geometridae	*Eupithecia pyreneata*	small	cold	monophagous	pupa	353
Geometridae	*Eupithecia satyrata*	small	cold	polyphagous	pupa	580

Geometridae	*Eupithecia schiefereri*	small	warm	monophagous	pupa	96
Geometridae	*Eupithecia selinata*	small	cold	polyphagous	pupa	382
Geometridae	*Eupithecia semigraphata*	small	warm	polyphagous	pupa	396
Geometridae	*Eupithecia silenata*	small	cold	monophagous	pupa	161
Geometridae	*Eupithecia sinuosaria*	small	cold	polyphagous	pupa	120
Geometridae	*Eupithecia subfuscata*	small	cold	polyphagous	pupa	5460
Geometridae	*Eupithecia subumbrata*	small	intermediate	polyphagous	pupa	310
Geometridae	*Eupithecia succenturiata*	small	cold	oligophagous	pupa	13
Geometridae	*Eupithecia tantillaria*	small	cold	oligophagous	pupa	6948
Geometridae	*Eupithecia tenuiata*	small	cold	monophagous	egg	308
Geometridae	*Eupithecia tripunctaria*	small	intermediate	polyphagous	pupa	145
Geometridae	*Eupithecia trisignaria*	small	cold	oligophagous	pupa	106
Geometridae	*Eupithecia undata*	small	cold	oligophagous	pupa	25
Geometridae	*Eupithecia valerianata*	small	cold	monophagous	pupa	92
Geometridae	*Eupithecia venosata*	small	intermediate	monophagous	pupa	328
Geometridae	*Eupithecia veratraria*	small	cold	monophagous	pupa	592
Geometridae	*Eupithecia virgaureata*	small	cold	polyphagous	pupa	360
Geometridae	*Eupithecia vulgata*	small	cold	polyphagous	pupa	651
Geometridae	*Eustroma reticulata*	small	cold	monophagous	pupa	231
Geometridae	*Fagivorina arenaria*	small	warm	polyphagous	pupa	381
Geometridae	*Gagitodes sagittata*	small	cold	monophagous	pupa	40
Geometridae	*Gandaritis pyraliata*	small	intermediate	monophagous	egg	1942
Geometridae	*Geometra papilionaria*	medium	cold	polyphagous	larva	954
Geometridae	*Gnophos furvata*	medium	warm	polyphagous	larva	613
Geometridae	*Gnophos obfuscata*	medium	cold	polyphagous	larva	4678
Geometridae	*Gymnoscelis rufifasciata*	small	warm	polyphagous	pupa	1870
Geometridae	*Heliomata glarearia*	small	warm	oligophagous	pupa	660
Geometridae	*Hemistola chrysoprasaria*	small	warm	monophagous	larva	2016
Geometridae	*Hemithea aestivaria*	small	warm	polyphagous	larva	4517
Geometridae	*Horisme aemulata*	small	cold	oligophagous	pupa	633
Geometridae	*Horisme calligraphata*	small	cold	monophagous	pupa	207
Geometridae	*Horisme radicaria*	small	warm	monophagous	pupa	1167
Geometridae	*Horisme tersata*	small	intermediate	polyphagous	pupa	1938
Geometridae	*Horisme vitalbata*	small	warm	monophagous	pupa	2452
Geometridae	*Hydrelia flammeolaria*	small	intermediate	polyphagous	pupa	2314
Geometridae	*Hydrelia sylvata*	small	cold	polyphagous	pupa	702
Geometridae	*Hydria cervinalis*	medium	intermediate	monophagous	pupa	1401
Geometridae	*Hydria montivagata*	small	intermediate	monophagous	pupa	149
Geometridae	*Hydria undulata*	small	intermediate	polyphagous	pupa	128
Geometridae	*Hydriomena furcata*	small	cold	polyphagous	egg	11510
Geometridae	*Hydriomena impluviata*	small	cold	monophagous	pupa	4068
Geometridae	*Hydriomena ruberata*	medium	cold	monophagous	pupa	867
Geometridae	*Hylaea fasciaria*	small	cold	oligophagous	larva	2282
Geometridae	*Hypomecis punctinalis*	medium	warm	polyphagous	pupa	7150
Geometridae	*Hypomecis roboraria*	medium	intermediate	oligophagous	larva	4612
Geometridae	*Idaea aureolaria*	small	intermediate	polyphagous	larva	51
Geometridae	*Idaea aversata*	small	intermediate	polyphagous	larva	21608
Geometridae	*Idaea biselata*	small	intermediate	polyphagous	larva	14698
Geometridae	*Idaea calunetaria*	small	warm	polyphagous	pupa	202
Geometridae	*Idaea contiguaria*	small	warm	polyphagous	larva	168
Geometridae	*Idaea degeneraria*	small	warm	polyphagous	larva	1627
Geometridae	*Idaea deversaria*	small	intermediate	polyphagous	larva	4634
Geometridae	*Idaea dilutaria*	small	warm	monophagous	larva	1537
Geometridae	*Idaea dimidiata*	small	intermediate	polyphagous	larva	956
Geometridae	*Idaea emarginata*	small	intermediate	polyphagous	larva	17
Geometridae	*Idaea flaveolaria*	small	cold	polyphagous	larva	13
Geometridae	*Idaea fuscovenosa*	small	warm	polyphagous	larva	467
Geometridae	*Idaea humiliata*	small	warm	polyphagous	larva	2154
Geometridae	*Idaea inquinata*	small	warm	polyphagous	larva	4
Geometridae	*Idaea laevigata*	small	warm	polyphagous	larva	1
Geometridae	*Idaea macilentaria*	small	warm	polyphagous	larva	52
Geometridae	*Idaea moniliata*	small	warm	polyphagous	larva	578
Geometridae	*Idaea muricata*	small	intermediate	polyphagous	larva	705
Geometridae	*Idaea nitidata*	small	intermediate	polyphagous	larva	5
Geometridae	*Idaea obsoletaria*	small	warm	polyphagous	larva	539
Geometridae	*Idaea ochrata*	small	warm	polyphagous	larva	1225
Geometridae	*Idaea politaria*	small	warm	polyphagous	larva	4
Geometridae	*Idaea rubraria*	small	warm	polyphagous	larva	188
Geometridae	*Idaea rufaria*	small	warm	polyphagous	larva	25
Geometridae	*Idaea rusticata*	small	warm	polyphagous	larva	2063
Geometridae	*Idaea seriata*	small	warm	polyphagous	larva	106
Geometridae	*Idaea sericeata*	small	warm	polyphagous	larva	87
Geometridae	*Idaea serpentata*	small	cold	polyphagous	larva	8
Geometridae	*Idaea straminata*	small	intermediate	polyphagous	larva	1490
Geometridae	*Idaea subsericeata*	small	warm	polyphagous	larva	1239
Geometridae	*Idaea sylvestraria*	small	intermediate	polyphagous	larva	19
Geometridae	*Idaea trigeminata*	small	warm	polyphagous	larva	8
Geometridae	*Idaea typicata*	small	warm	polyphagous	larva	1447

Geometridae	*Isturgia arenacearia*	small	warm	oligophagous	pupa	9
Geometridae	*Jodis lactearia*	small	intermediate	polyphagous	pupa	1177
Geometridae	*Jodis putata*	small	cold	oligophagous	pupa	142
Geometridae	*Lampropteryx suffumata*	small	cold	monophagous	pupa	1219
Geometridae	*Larentia clavaria*	medium	intermediate	oligophagous	egg	1
Geometridae	*Ligdia adustata*	small	warm	monophagous	pupa	10705
Geometridae	*Lobophora halterata*	small	intermediate	oligophagous	pupa	847
Geometridae	*Lomaspilis marginata*	small	intermediate	polyphagous	pupa	12697
Geometridae	*Lomographa bimaculata*	small	intermediate	polyphagous	pupa	2174
Geometridae	*Lomographa temerata*	small	intermediate	polyphagous	pupa	5695
Geometridae	*Lycia alpina*	medium	cold	polyphagous	pupa	2358
Geometridae	*Lycia hirtaria*	medium	intermediate	polyphagous	pupa	6121
Geometridae	*Lycia zonaria*	small	intermediate	polyphagous	pupa	2
Geometridae	*Macaria alternata*	small	intermediate	polyphagous	pupa	6938
Geometridae	*Macaria artesiaria*	small	cold	monophagous	egg	72
Geometridae	*Macaria brunneata*	small	cold	monophagous	egg	887
Geometridae	*Macaria liturata*	small	intermediate	oligophagous	pupa	6451
Geometridae	*Macaria notata*	small	intermediate	polyphagous	pupa	3510
Geometridae	*Macaria signaria*	small	cold	oligophagous	larva	466
Geometridae	*Macaria wauaria*	small	cold	monophagous	egg	184
Geometridae	*Martania taeniata*	small	cold	polyphagous	larva	290
Geometridae	*Melanthia alaudaria*	small	cold	monophagous	pupa	8
Geometridae	*Melanthia procellata*	medium	warm	monophagous	pupa	3594
Geometridae	*Menophra abruptaria*	medium	warm	polyphagous	pupa	870
Geometridae	*Menophra nycthemeraria*	medium	warm	polyphagous	larva	16
Geometridae	*Mesoleuca albicillata*	small	intermediate	oligophagous	pupa	581
Geometridae	*Mesotype didymata*	small	cold	polyphagous	egg	284
Geometridae	*Mesotype parallelolineata*	small	cold	polyphagous	egg	428
Geometridae	*Mesotype verberata*	small	cold	polyphagous	egg	10168
Geometridae	*Minoa murinata*	small	warm	monophagous	pupa	998
Geometridae	*Nebula achromaria*	small	intermediate	monophagous	larva	271
Geometridae	*Nebula nebulata*	small	cold	monophagous	larva	1480
Geometridae	*Nothocasis sertata*	small	intermediate	monophagous	egg	846
Geometridae	*Odontopera bidentata*	medium	cold	polyphagous	pupa	4917
Geometridae	*Operophtera brumata*	small	cold	polyphagous	egg	5898
Geometridae	*Operophtera fagata*	medium	cold	polyphagous	egg	2029
Geometridae	*Opisthograptis luteolata*	medium	intermediate	polyphagous	pupa	12102
Geometridae	*Orthonama obstipata*	small	warm	polyphagous	pupa	695
Geometridae	*Orthonama vittata*	small	cold	polyphagous	larva	649
Geometridae	*Ourapteryx sambucaria*	medium	intermediate	polyphagous	larva	403
Geometridae	*Pachycnemia hippocastanaria*	small	warm	monophagous	pupa	1
Geometridae	*Paradarisa consonaria*	medium	intermediate	polyphagous	pupa	2380
Geometridae	*Parectropis similaria*	small	warm	polyphagous	pupa	2977
Geometridae	*Pareulype berberata*	small	intermediate	monophagous	pupa	3042
Geometridae	*Pasiphila chloerata*	small	cold	monophagous	egg	43
Geometridae	*Pasiphila debiliata*	small	cold	monophagous	egg	304
Geometridae	*Pasiphila rectangulata*	small	intermediate	oligophagous	egg	1622
Geometridae	*Pelurga comitata*	small	intermediate	oligophagous	pupa	26
Geometridae	*Pennithera firmata*	small	cold	monophagous	larva	2540
Geometridae	*Perconia strigillaria*	medium	intermediate	polyphagous	larva	444
Geometridae	*Peribatodes perversaria*	medium	warm	monophagous	larva	177
Geometridae	*Peribatodes rhomboidaria*	medium	warm	polyphagous	larva	14814
Geometridae	*Peribatodes secundaria*	medium	intermediate	oligophagous	larva	7330
Geometridae	*Perizoma affinitata*	small	cold	monophagous	pupa	498
Geometridae	*Perizoma albulata*	small	cold	monophagous	pupa	4352
Geometridae	*Perizoma alchemillata*	small	cold	oligophagous	pupa	11263
Geometridae	*Perizoma bifaciata*	small	intermediate	oligophagous	pupa	94
Geometridae	*Perizoma blandiata*	small	cold	monophagous	pupa	1436
Geometridae	*Perizoma flavofasciata*	small	intermediate	monophagous	pupa	169
Geometridae	*Perizoma hydrata*	small	cold	monophagous	pupa	2019
Geometridae	*Perizoma incultaria*	small	cold	polyphagous	pupa	337
Geometridae	*Perizoma juracolaria*	small	intermediate	monophagous	pupa	25
Geometridae	*Perizoma lugdunaria*	small	warm	monophagous	pupa	113
Geometridae	*Perizoma minorata*	small	cold	monophagous	pupa	3113
Geometridae	*Perizoma obsoletata*	small	cold	monophagous	pupa	321
Geometridae	*Petrophora chlorosata*	small	intermediate	monophagous	pupa	1954
Geometridae	*Phaiogramma etruscaria*	small	warm	polyphagous	pupa	45
Geometridae	*Phigalia pilosaria*	medium	intermediate	polyphagous	pupa	798
Geometridae	*Philereme transversata*	medium	warm	oligophagous	egg	285
Geometridae	*Philereme vetulata*	small	intermediate	oligophagous	egg	1411
Geometridae	*Plagodis dolabraria*	small	warm	polyphagous	pupa	4419
Geometridae	*Plagodis pulveraria*	small	cold	polyphagous	pupa	3858
Geometridae	*Plemyria rubiginata*	small	cold	polyphagous	egg	579
Geometridae	*Pseudoterpna pruinata*	medium	warm	oligophagous	larva	584
Geometridae	*Pterapherapteryx sexalata*	small	cold	oligophagous	pupa	253
Geometridae	*Pungeleria capreolaria*	small	warm	oligophagous	larva	1050
Geometridae	*Rheumaptera subhastata*	small	cold	monophagous	pupa	1
Geometridae	*Rhodometra sacraria*	small	warm	polyphagous	NA	276

Geometridae	*Rhodostrophia calabra*	small	warm	polyphagous	larva	260
Geometridae	*Rhodostrophia vibicaria*	small	intermediate	polyphagous	larva	957
Geometridae	*Sciadia zelleraria*	medium	cold	polyphagous	larva	92
Geometridae	*Scopula caricaria*	small	intermediate	polyphagous	larva	2578
Geometridae	*Scopula confinaria*	small	warm	NA	larva	265
Geometridae	*Scopula decorata*	small	warm	monophagous	larva	40
Geometridae	*Scopula floslactata*	small	cold	polyphagous	larva	1015
Geometridae	*Scopula imitaria*	small	warm	polyphagous	larva	808
Geometridae	*Scopula immorata*	small	cold	polyphagous	larva	1390
Geometridae	*Scopula immutata*	small	intermediate	polyphagous	larva	1130
Geometridae	*Scopula incanata*	small	cold	polyphagous	larva	3011
Geometridae	*Scopula marginepunctata*	small	warm	polyphagous	larva	2770
Geometridae	*Scopula nigropunctata*	small	warm	polyphagous	larva	3711
Geometridae	*Scopula ornata*	small	warm	polyphagous	larva	1384
Geometridae	*Scopula rubiginata*	small	warm	polyphagous	larva	12
Geometridae	*Scopula submutata*	small	warm	oligophagous	larva	29
Geometridae	*Scopula subpunctaria*	small	warm	polyphagous	larva	1983
Geometridae	*Scopula ternata*	small	cold	oligophagous	larva	900
Geometridae	*Scopula umbelaria*	small	warm	polyphagous	larva	76
Geometridae	*Scopula virgulata*	small	intermediate	polyphagous	larva	236
Geometridae	*Scotopteryx angularia*	medium	warm	oligophagous	larva	19
Geometridae	*Scotopteryx bipunctaria*	small	warm	polyphagous	larva	1829
Geometridae	*Scotopteryx chenopodiata*	small	cold	oligophagous	larva	7737
Geometridae	*Scotopteryx luridata*	medium	warm	oligophagous	larva	1406
Geometridae	*Scotopteryx moeniata*	small	warm	oligophagous	larva	319
Geometridae	*Scotopteryx mucronata*	small	warm	oligophagous	larva	28
Geometridae	*Scotopteryx octodurensis*	small	intermediate	monophagous	larva	145
Geometridae	*Scotopteryx vicinaria*	small	cold	NA	pupa	83
Geometridae	*Selenia dentaria*	medium	cold	polyphagous	pupa	3475
Geometridae	*Selenia lunularia*	medium	warm	polyphagous	pupa	1800
Geometridae	*Selenia tetralunaria*	medium	intermediate	polyphagous	pupa	4326
Geometridae	*Selidosema brunnearia*	small	warm	polyphagous	larva	230
Geometridae	*Selidosema plumaria*	medium	warm	polyphagous	larva	45
Geometridae	*Siona lineata*	medium	intermediate	polyphagous	larva	1586
Geometridae	*Spargania luctuata*	small	cold	monophagous	pupa	24
Geometridae	*Stegania cararia*	small	intermediate	polyphagous	pupa	208
Geometridae	*Stegania trimaculata*	small	warm	monophagous	pupa	474
Geometridae	*Synopsia sociaria*	medium	warm	polyphagous	larva	39
Geometridae	*Tephronia sepiaria*	small	warm	polyphagous	larva	224
Geometridae	*Thalera fimbrialis*	small	warm	polyphagous	larva	1042
Geometridae	*Thera britannica*	small	intermediate	oligophagous	larva	10178
Geometridae	*Thera cembrae*	small	cold	monophagous	larva	469
Geometridae	*Thera cognata*	small	cold	monophagous	egg	8208
Geometridae	*Thera cupressata*	small	warm	monophagous	egg	33
Geometridae	*Thera juniperata*	small	cold	monophagous	egg	339
Geometridae	*Thera obeliscata*	small	cold	monophagous	larva	992
Geometridae	*Thera variata*	small	cold	oligophagous	larva	11005
Geometridae	*Thera vetustata*	small	intermediate	oligophagous	larva	908
Geometridae	*Theria primaria*	medium	warm	oligophagous	pupa	32
Geometridae	*Theria rupicapraria*	small	warm	oligophagous	pupa	8
Geometridae	*Thetidia smaragdaria*	small	intermediate	oligophagous	larva	50
Geometridae	*Timandra comae*	small	warm	oligophagous	larva	1896
Geometridae	*Trichopteryx carpinata*	small	cold	polyphagous	pupa	590
Geometridae	*Trichopteryx polycommata*	small	intermediate	polyphagous	pupa	109
Geometridae	*Triphosa dubitata*	medium	intermediate	oligophagous	adult	902
Geometridae	*Triphosa sabaudiata*	medium	intermediate	monophagous	adult	50
Geometridae	*Triphosa tauteli*	medium	warm	monophagous	adult	3
Geometridae	*Venusia blomeri*	small	cold	monophagous	pupa	731
Geometridae	*Venusia cambrica*	small	cold	polyphagous	pupa	73
Geometridae	*Xanthorhoe biriviata*	small	cold	monophagous	pupa	1336
Geometridae	*Xanthorhoe decoloraria*	small	cold	monophagous	larva	1314
Geometridae	*Xanthorhoe designata*	small	cold	oligophagous	pupa	2046
Geometridae	*Xanthorhoe ferrugata*	small	intermediate	polyphagous	pupa	6263
Geometridae	*Xanthorhoe fluctuata*	small	intermediate	oligophagous	pupa	3842
Geometridae	*Xanthorhoe incursata*	small	cold	monophagous	pupa	47
Geometridae	*Xanthorhoe montanata*	small	cold	polyphagous	larva	11670
Geometridae	*Xanthorhoe quadrifasiata*	small	cold	polyphagous	larva	897
Geometridae	*Xanthorhoe spadicearia*	small	cold	polyphagous	pupa	5627
Geometridae	*Yezognophos dilucidaria*	small	cold	polyphagous	larva	3944
Geometridae	*Yezognophos serotinaria*	medium	cold	polyphagous	larva	230
Geometridae	*Yezognophos vittaria*	medium	cold	polyphagous	larva	325
Hepialidae	*Gazoryctra ganna*	medium	cold	polyphagous	NA	5
Hepialidae	*Hepialus humuli*	large	cold	polyphagous	larva	544
Hepialidae	*Korscheltellus fusconebulosa*	large	cold	polyphagous	larva	200
Hepialidae	*Korscheltellus lupulina*	medium	warm	polyphagous	larva	240
Hepialidae	*Phymatopus hecta*	medium	cold	polyphagous	larva	24
Hepialidae	*Triodia sylvina*	large	warm	polyphagous	larva	1094
Lasiocampidae	*Cosmotriche lobulina*	large	cold	oligophagous	larva	157

Lasiocampidae	*Dendrolimus pini*	large	intermediate	oligophagous	larva	2098
Lasiocampidae	*Eriogaster arbusculae*	large	cold	polyphagous	pupa	62
Lasiocampidae	*Eriogaster catax*	large	warm	polyphagous	egg	3
Lasiocampidae	*Eriogaster lanestris*	large	cold	polyphagous	pupa	3
Lasiocampidae	*Euthrix potatoria*	large	intermediate	polyphagous	larva	1415
Lasiocampidae	*Gastropacha quercifolia*	large	warm	polyphagous	larva	1
Lasiocampidae	*Lasiocampa quercus*	large	intermediate	polyphagous	larva	350
Lasiocampidae	*Lasiocampa trifolii*	large	warm	polyphagous	egg	538
Lasiocampidae	*Macrothylacia rubi*	large	intermediate	polyphagous	larva	642
Lasiocampidae	*Malacosoma alpicola*	medium	cold	polyphagous	egg	1
Lasiocampidae	*Malacosoma castrensis*	large	intermediate	polyphagous	egg	36
Lasiocampidae	*Malacosoma neustria*	medium	warm	polyphagous	egg	1192
Lasiocampidae	*Odonestis pruni*	large	warm	polyphagous	larva	84
Lasiocampidae	*Phyllodesma tremulifolia*	medium	warm	polyphagous	pupa	242
Lasiocampidae	*Poecilocampa alpina*	large	intermediate	monophagous	egg	612
Lasiocampidae	*Poecilocampa populi*	large	cold	polyphagous	egg	3080
Lasiocampidae	*Trichiura crataegi*	medium	cold	polyphagous	egg	2522
Noctuidae	*Abrostola agnorista*	medium	warm	oligophagous	pupa	291
Noctuidae	*Abrostola asclepiadis*	large	intermediate	monophagous	pupa	1831
Noctuidae	*Abrostola tripartita*	medium	cold	monophagous	pupa	1474
Noctuidae	*Abrostola triplasia*	medium	intermediate	monophagous	pupa	1067
Noctuidae	*Acontia trabealis*	medium	warm	monophagous	pupa	1483
Noctuidae	*Acosmetia caliginosa*	medium	warm	monophagous	pupa	5
Noctuidae	*Acronicta aceris*	large	warm	polyphagous	pupa	197
Noctuidae	*Acronicta alni*	medium	intermediate	polyphagous	pupa	1417
Noctuidae	*Acronicta auricoma*	large	cold	polyphagous	pupa	430
Noctuidae	*Acronicta cuspis*	large	cold	oligophagous	pupa	107
Noctuidae	*Acronicta euphorbiae*	large	warm	polyphagous	pupa	790
Noctuidae	*Acronicta leporina*	large	intermediate	polyphagous	pupa	527
Noctuidae	*Acronicta megacephala*	large	intermediate	polyphagous	pupa	410
Noctuidae	*Acronicta menyanthidis*	large	cold	polyphagous	pupa	10
Noctuidae	*Acronicta psi*	large	intermediate	polyphagous	pupa	1570
Noctuidae	*Acronicta rumicis*	large	warm	polyphagous	pupa	1484
Noctuidae	*Acronicta strigosa*	large	cold	oligophagous	pupa	21
Noctuidae	*Acronicta tridens*	large	warm	polyphagous	pupa	1
Noctuidae	*Actebia multifida*	large	cold	polyphagous	larva	35
Noctuidae	*Actebia praecox*	large	cold	polyphagous	larva	54
Noctuidae	*Actinotia polyodon*	medium	intermediate	monophagous	pupa	470
Noctuidae	*Aedia funesta*	medium	warm	oligophagous	larva	83
Noctuidae	*Aedia leucomelas*	large	warm	oligophagous	larva	1216
Noctuidae	*Agrochola helvola*	large	cold	polyphagous	egg	1380
Noctuidae	*Agrochola laevis*	large	warm	polyphagous	egg	53
Noctuidae	*Agrochola litura*	medium	intermediate	polyphagous	egg	4220
Noctuidae	*Agrochola lota*	large	intermediate	polyphagous	egg	555
Noctuidae	*Agrochola lychnidis*	large	warm	polyphagous	egg	844
Noctuidae	*Agrochola macilenta*	large	intermediate	polyphagous	egg	15784
Noctuidae	*Agrochola nitida*	medium	intermediate	polyphagous	egg	227
Noctuidae	*Agrochola pistacinoides*	medium	warm	NA	egg	414
Noctuidae	*Agrochola ruticilla*	medium	warm	monophagous	pupa	140
Noctuidae	*Agrotis bigramma*	large	warm	polyphagous	larva	6
Noctuidae	*Agrotis cinerea*	large	intermediate	polyphagous	larva	6612
Noctuidae	*Agrotis clavis*	large	intermediate	polyphagous	larva	4547
Noctuidae	*Agrotis exclamationis*	large	intermediate	polyphagous	larva	52482
Noctuidae	*Agrotis fatidica*	large	cold	oligophagous	larva	388
Noctuidae	*Agrotis ipsilon*	large	warm	polyphagous	larva	130576
Noctuidae	*Agrotis puta*	medium	warm	polyphagous	larva	14
Noctuidae	*Agrotis segetum*	large	warm	polyphagous	larva	2378
Noctuidae	*Agrotis simplonia*	large	cold	polyphagous	larva	3974
Noctuidae	*Agrotis trux*	large	warm	polyphagous	larva	1799
Noctuidae	*Agrotis vestigialis*	medium	intermediate	polyphagous	larva	71
Noctuidae	*Allophyes oxyacanthae*	large	intermediate	polyphagous	egg	1771
Noctuidae	*Ammoconia caecimacula*	large	intermediate	polyphagous	egg	1595
Noctuidae	*Ammoconia senex*	large	warm	polyphagous	egg	20
Noctuidae	*Amphipoea fucosa*	medium	cold	oligophagous	egg	42
Noctuidae	*Amphipoea lucens*	medium	cold	oligophagous	egg	166
Noctuidae	*Amphipoea oculea*	medium	cold	oligophagous	egg	1618
Noctuidae	*Amphipyra berbera*	large	intermediate	polyphagous	egg	346
Noctuidae	*Amphipyra cinnamomea*	large	cold	monophagous	adult	1
Noctuidae	*Amphipyra livida*	large	warm	polyphagous	egg	41
Noctuidae	*Amphipyra perflua*	large	cold	polyphagous	egg	83
Noctuidae	*Amphipyra pyramidea*	large	intermediate	polyphagous	egg	4204
Noctuidae	*Amphipyra tragopoginis*	large	intermediate	polyphagous	egg	1080
Noctuidae	*Anaplectoides prasina*	large	cold	polyphagous	larva	5548
Noctuidae	*Anarta melanopa*	medium	cold	polyphagous	pupa	1
Noctuidae	*Anarta myrtilli*	medium	intermediate	oligophagous	pupa	56
Noctuidae	*Anarta odontites*	medium	intermediate	monophagous	pupa	6959
Noctuidae	*Anarta trifolii*	large	intermediate	polyphagous	pupa	1346
Noctuidae	*Anorthoa munda*	large	warm	polyphagous	pupa	4665

Noctuidae	*Antitype chi*	large	cold	polyphagous	egg	630
Noctuidae	*Antitype suda*	medium	intermediate	polyphagous	egg	19
Noctuidae	*Apamea anceps*	large	warm	oligophagous	larva	699
Noctuidae	*Apamea aquila*	large	intermediate	monophagous	larva	468
Noctuidae	*Apamea crenata*	large	cold	oligophagous	larva	3934
Noctuidae	*Apamea epomidion*	large	warm	oligophagous	larva	239
Noctuidae	*Apamea furva*	large	cold	oligophagous	larva	974
Noctuidae	*Apamea illyria*	medium	cold	oligophagous	larva	541
Noctuidae	*Apamea lateritia*	large	cold	oligophagous	larva	1436
Noctuidae	*Apamea lithoxylaea*	large	warm	oligophagous	larva	502
Noctuidae	*Apamea maillardi*	large	cold	oligophagous	larva	4468
Noctuidae	*Apamea monoglypha*	large	intermediate	oligophagous	larva	21283
Noctuidae	*Apamea platinea*	large	cold	oligophagous	larva	150
Noctuidae	*Apamea remissa*	large	cold	oligophagous	larva	122
Noctuidae	*Apamea rubrirena*	large	cold	oligophagous	larva	1107
Noctuidae	*Apamea scolopacina*	medium	intermediate	polyphagous	larva	1480
Noctuidae	*Apamea sordens*	large	intermediate	oligophagous	larva	488
Noctuidae	*Apamea sublustris*	large	intermediate	oligophagous	larva	1764
Noctuidae	*Apamea unanimis*	medium	intermediate	oligophagous	larva	166
Noctuidae	*Apamea zeta*	large	cold	oligophagous	larva	1535
Noctuidae	*Aporophyla lutulenta*	large	warm	polyphagous	larva	1043
Noctuidae	*Aporophyla nigra*	large	warm	polyphagous	larva	1
Noctuidae	*Apterogenum ypsillon*	large	warm	oligophagous	egg	342
Noctuidae	*Archanara dissoluta*	medium	intermediate	monophagous	egg	9
Noctuidae	*Archanara neurica*	medium	intermediate	oligophagous	egg	83
Noctuidae	*Asteroscopus sphinx*	large	warm	polyphagous	egg	430
Noctuidae	*Atethmia centrago*	medium	warm	monophagous	egg	695
Noctuidae	*Athetis gluteosa*	medium	cold	polyphagous	larva	922
Noctuidae	*Athetis hospes*	medium	warm	polyphagous	larva	1196
Noctuidae	*Athetis pallustris*	medium	cold	monophagous	larva	437
Noctuidae	*Atypha pulmonaris*	medium	warm	monophagous	egg	749
Noctuidae	*Auchmis detersa*	large	cold	monophagous	larva	677
Noctuidae	*Autographa aemula*	large	cold	polyphagous	larva	1680
Noctuidae	*Autographa bractea*	large	cold	polyphagous	larva	1773
Noctuidae	*Autographa gamma*	large	intermediate	polyphagous	larva	146440
Noctuidae	*Autographa jota*	large	intermediate	polyphagous	larva	848
Noctuidae	*Autographa pulchrina*	large	cold	polyphagous	larva	2073
Noctuidae	*Axylia putris*	medium	intermediate	polyphagous	pupa	16798
Noctuidae	*Brachionycha nubeculosa*	large	cold	polyphagous	pupa	587
Noctuidae	*Brachylomia viminalis*	medium	cold	oligophagous	egg	3166
Noctuidae	*Bryophila domestica*	medium	warm	oligophagous	larva	124
Noctuidae	*Bryophila ereptricula*	medium	cold	polyphagous	larva	26
Noctuidae	*Bryophila petricolor*	medium	cold	NA	larva	50
Noctuidae	*Bryophila raptricula*	medium	intermediate	polyphagous	larva	528
Noctuidae	*Bryophila ravula*	medium	warm	polyphagous	larva	206
Noctuidae	*Bryopsis muralis*	medium	warm	polyphagous	larva	490
Noctuidae	*Calamia tridens*	large	intermediate	oligophagous	egg	471
Noctuidae	*Calliergis ramosa*	medium	cold	monophagous	pupa	368
Noctuidae	*Callopistria juventina*	large	warm	monophagous	larva	1332
Noctuidae	*Callopistria latreillei*	medium	warm	polyphagous	egg	507
Noctuidae	*Calophasia lunula*	medium	intermediate	oligophagous	pupa	251
Noctuidae	*Calophasia platyptera*	medium	warm	oligophagous	pupa	8
Noctuidae	*Caradrina aspersa*	medium	warm	polyphagous	larva	562
Noctuidae	*Caradrina clavipalpis*	medium	intermediate	polyphagous	larva	3554
Noctuidae	*Caradrina flavirena*	medium	warm	polyphagous	larva	1243
Noctuidae	*Caradrina gilva*	medium	cold	polyphagous	larva	76
Noctuidae	*Caradrina kadenii*	medium	warm	polyphagous	larva	375
Noctuidae	*Caradrina montana*	medium	cold	polyphagous	larva	6
Noctuidae	*Caradrina morpheus*	large	intermediate	polyphagous	larva	1143
Noctuidae	*Caradrina selini*	medium	cold	polyphagous	larva	2250
Noctuidae	*Caradrina terrea*	medium	cold	polyphagous	larva	135
Noctuidae	*Caradrina wullschlegeli*	medium	warm	polyphagous	larva	320
Noctuidae	*Ceramica pisi*	large	cold	polyphagous	pupa	19215
Noctuidae	*Cerapteryx graminis*	medium	cold	polyphagous	larva	2890
Noctuidae	*Cerastis leucographa*	large	cold	polyphagous	pupa	215
Noctuidae	*Cerastis rubricosa*	large	intermediate	polyphagous	pupa	5293
Noctuidae	*Charanyca trigrammica*	large	warm	polyphagous	larva	5865
Noctuidae	*Chersotis alpestris*	medium	cold	polyphagous	larva	174
Noctuidae	*Chersotis andereggii*	medium	cold	polyphagous	larva	103
Noctuidae	*Chersotis cuprea*	large	cold	polyphagous	larva	10703
Noctuidae	*Chersotis fimbriola*	medium	warm	polyphagous	larva	128
Noctuidae	*Chersotis margaritacea*	large	intermediate	oligophagous	larva	1092
Noctuidae	*Chersotis multangula*	medium	intermediate	monophagous	larva	871
Noctuidae	*Chersotis ocellina*	medium	cold	polyphagous	larva	5184
Noctuidae	*Chersotis oreina*	medium	cold	polyphagous	larva	1036
Noctuidae	*Chersotis rectangula*	medium	cold	oligophagous	larva	31
Noctuidae	*Chilodes maritima*	medium	intermediate	monophagous	larva	220
Noctuidae	*Chloantha hyperici*	medium	warm	monophagous	pupa	590

Noctuidae	*Chrysodeixis chalcites*	large	warm	polyphagous	NA	298
Noctuidae	*Cirrhia gilvago*	large	warm	polyphagous	egg	22
Noctuidae	*Cirrhia icteritia*	medium	cold	polyphagous	egg	667
Noctuidae	*Cirrhia ocellaris*	large	warm	monophagous	egg	13
Noctuidae	*Clemathada calberlai*	medium	warm	monophagous	pupa	400
Noctuidae	*Colocasia coryli*	medium	intermediate	polyphagous	pupa	9345
Noctuidae	*Conistra erythrocephala*	large	warm	polyphagous	adult	2200
Noctuidae	*Conistra ligula*	medium	warm	polyphagous	adult	82
Noctuidae	*Conistra rubiginea*	medium	intermediate	polyphagous	adult	6472
Noctuidae	*Conistra rubiginosa*	medium	intermediate	polyphagous	adult	981
Noctuidae	*Conistra torrida*	large	intermediate	polyphagous	adult	1
Noctuidae	*Conistra vaccinii*	medium	intermediate	polyphagous	adult	16032
Noctuidae	*Cosmia affinis*	medium	warm	polyphagous	egg	178
Noctuidae	*Cosmia pyralina*	medium	intermediate	polyphagous	egg	162
Noctuidae	*Cosmia trapezina*	medium	intermediate	polyphagous	egg	14132
Noctuidae	*Craniophora ligustri*	medium	warm	polyphagous	pupa	13553
Noctuidae	*Cryphia algae*	medium	warm	polyphagous	larva	1738
Noctuidae	*Cryphia ochsi*	medium	warm	polyphagous	larva	87
Noctuidae	*Cryphia simulatricula*	medium	warm	polyphagous	larva	3
Noctuidae	*Crypsedra gemmea*	large	cold	oligophagous	egg	772
Noctuidae	*Cucullia absinthii*	large	intermediate	monophagous	pupa	8
Noctuidae	*Cucullia artemisiae*	large	intermediate	monophagous	pupa	1
Noctuidae	*Cucullia asteris*	large	intermediate	oligophagous	pupa	23
Noctuidae	*Cucullia campanulae*	large	cold	monophagous	pupa	116
Noctuidae	*Cucullia gnaphalii*	large	cold	oligophagous	pupa	24
Noctuidae	*Cucullia lactucae*	large	cold	oligophagous	pupa	64
Noctuidae	*Cucullia lucifuga*	large	cold	oligophagous	pupa	537
Noctuidae	*Cucullia lychnitis*	large	warm	monophagous	pupa	22
Noctuidae	*Cucullia prenanthis*	large	cold	monophagous	pupa	70
Noctuidae	*Cucullia santonici*	large	cold	oligophagous	pupa	17
Noctuidae	*Cucullia scrophulariae*	large	warm	oligophagous	pupa	6
Noctuidae	*Cucullia thapsiphaga*	large	warm	monophagous	pupa	1
Noctuidae	*Cucullia umbratica*	large	intermediate	oligophagous	pupa	566
Noctuidae	*Cucullia verbasci*	large	warm	oligophagous	pupa	29
Noctuidae	*Dasypolia ferdinandi*	large	cold	monophagous	adult	2
Noctuidae	*Dasypolia templi*	large	cold	oligophagous	adult	983
Noctuidae	*Deltote bankiana*	medium	intermediate	oligophagous	pupa	127
Noctuidae	*Deltote deceptoria*	medium	intermediate	oligophagous	pupa	157
Noctuidae	*Deltote pygarga*	medium	intermediate	oligophagous	pupa	15519
Noctuidae	*Deltote uncula*	medium	intermediate	oligophagous	pupa	669
Noctuidae	*Denticucullus pygmina*	medium	intermediate	polyphagous	larva	245
Noctuidae	*Diachrysia chrysitis* aggr.	medium	intermediate	polyphagous	larva	3498
Noctuidae	*Diachrysia chryson*	large	intermediate	monophagous	larva	114
Noctuidae	*Diachrysia nadeja*	large	intermediate	polyphagous	larva	45
Noctuidae	*Diarsia brunnea*	large	cold	polyphagous	larva	7815
Noctuidae	*Diarsia dahlii*	medium	cold	polyphagous	larva	294
Noctuidae	*Diarsia mendica*	medium	cold	polyphagous	larva	7214
Noctuidae	*Diarsia rubi*	medium	cold	polyphagous	larva	1681
Noctuidae	*Dichagyris candelisequa*	large	intermediate	polyphagous	larva	144
Noctuidae	*Dichagyris flammatra*	large	warm	polyphagous	larva	7
Noctuidae	*Dichagyris forcipula*	large	intermediate	polyphagous	larva	198
Noctuidae	*Dichagyris musiva*	large	cold	polyphagous	larva	171
Noctuidae	*Dichagyris nigrescens*	large	cold	polyphagous	larva	359
Noctuidae	*Dichagyris signifera*	large	cold	polyphagous	larva	469
Noctuidae	*Dichagyris vallesiaca*	medium	cold	polyphagous	larva	1
Noctuidae	*Dichonia convergens*	large	warm	monophagous	egg	426
Noctuidae	*Dicycla oo*	large	warm	monophagous	egg	22
Noctuidae	*Diloba caeruleocephala*	large	warm	oligophagous	egg	1724
Noctuidae	*Dryobotodes eremita*	large	warm	monophagous	egg	567
Noctuidae	*Dypterygia scabriuscula*	large	intermediate	oligophagous	larva	1273
Noctuidae	*Egira conspicillaris*	large	warm	polyphagous	pupa	1302
Noctuidae	*Elaphria venustula*	medium	warm	polyphagous	pupa	1500
Noctuidae	*Enargia paleacea*	large	cold	polyphagous	egg	665
Noctuidae	*Enterpia laudeti*	medium	intermediate	oligophagous	pupa	6
Noctuidae	*Epilecta linogrisea*	large	warm	polyphagous	larva	488
Noctuidae	*Epimecia ustula*	medium	warm	polyphagous	pupa	3
Noctuidae	*Epipsilia grisescens*	medium	cold	oligophagous	larva	1929
Noctuidae	*Epipsilia latens*	large	cold	oligophagous	larva	47
Noctuidae	*Episema glaucina*	large	warm	oligophagous	larva	290
Noctuidae	*Eriopygodes imbecilla*	medium	cold	polyphagous	larva	3721
Noctuidae	*Eucarta amethystina*	medium	warm	oligophagous	pupa	27
Noctuidae	*Eucarta virgo*	medium	cold	oligophagous	pupa	136
Noctuidae	*Euchalcia modestoides*	medium	cold	oligophagous	larva	12
Noctuidae	*Euchalcia variabilis*	large	cold	oligophagous	larva	2338
Noctuidae	*Eugnorisma depuncta*	large	intermediate	polyphagous	larva	3464
Noctuidae	*Eugnorisma glareosa*	large	warm	polyphagous	larva	361
Noctuidae	*Eugraphe sigma*	large	cold	polyphagous	larva	1097
Noctuidae	*Euplexia lucipara*	medium	intermediate	polyphagous	pupa	4976

Noctuidae	*Eupsilia transversa*	large	intermediate	polyphagous	adult	5841
Noctuidae	*Eurois occulta*	large	cold	polyphagous	larva	293
Noctuidae	*Euxoa aquilina*	large	intermediate	polyphagous	larva	114
Noctuidae	*Euxoa birivia*	large	cold	NA	larva	3
Noctuidae	*Euxoa cos*	large	warm	polyphagous	larva	12
Noctuidae	*Euxoa culminicola*	large	cold	polyphagous	larva	40
Noctuidae	*Euxoa decora*	large	cold	polyphagous	larva	1823
Noctuidae	*Euxoa distinguenda*	medium	cold	polyphagous	larva	25
Noctuidae	*Euxoa eruta*	medium	cold	polyphagous	larva	143
Noctuidae	*Euxoa nigricans*	medium	cold	polyphagous	larva	1027
Noctuidae	*Euxoa obelisca*	large	intermediate	polyphagous	larva	334
Noctuidae	*Euxoa recussa*	large	cold	polyphagous	larva	2435
Noctuidae	*Euxoa vitta*	large	cold	polyphagous	larva	35
Noctuidae	*Gortyna flavago*	large	intermediate	polyphagous	egg	189
Noctuidae	*Graphiphora augur*	large	cold	polyphagous	larva	788
Noctuidae	*Griposia aprilina*	large	intermediate	polyphagous	egg	291
Noctuidae	*Hada plebeja*	medium	cold	polyphagous	pupa	53849
Noctuidae	*Hadena albimacula*	medium	intermediate	monophagous	pupa	122
Noctuidae	*Hadena bicruris*	large	intermediate	oligophagous	pupa	210
Noctuidae	*Hadena caesia*	large	cold	monophagous	pupa	2767
Noctuidae	*Hadena compta*	medium	intermediate	oligophagous	pupa	1125
Noctuidae	*Hadena confusa*	medium	intermediate	oligophagous	pupa	908
Noctuidae	*Hadena filograna*	medium	intermediate	monophagous	pupa	134
Noctuidae	*Hadena irregularis*	medium	intermediate	oligophagous	pupa	17
Noctuidae	*Hadena luteocincta*	medium	warm	NA	NA	6
Noctuidae	*Hadena magnolii*	medium	warm	monophagous	pupa	163
Noctuidae	*Hadena perplexa*	medium	intermediate	oligophagous	pupa	1648
Noctuidae	*Hadena tephroleuca*	medium	cold	oligophagous	pupa	106
Noctuidae	*Hecatera bicolorata*	medium	intermediate	oligophagous	pupa	542
Noctuidae	*Hecatera dysodea*	medium	warm	oligophagous	pupa	50
Noctuidae	*Helicoverpa armigera*	large	warm	polyphagous	pupa	1683
Noctuidae	*Heliothis nubigera*	large	warm	polyphagous	pupa	9
Noctuidae	*Heliothis peltigera*	large	warm	polyphagous	pupa	454
Noctuidae	*Heliothis viriplaca*	medium	warm	polyphagous	pupa	19
Noctuidae	*Helotropha leucostigma*	large	cold	polyphagous	egg	623
Noctuidae	*Hoplodrina ambigua*	medium	warm	polyphagous	larva	10593
Noctuidae	*Hoplodrina blanda*	medium	intermediate	polyphagous	larva	19476
Noctuidae	*Hoplodrina octogenaria*	medium	intermediate	polyphagous	larva	40013
Noctuidae	*Hoplodrina respersa*	medium	intermediate	polyphagous	larva	4413
Noctuidae	*Hoplodrina superstes*	medium	warm	polyphagous	larva	863
Noctuidae	*Hydraecia micacea*	large	cold	polyphagous	egg	237
Noctuidae	*Hyppa rectilinea*	large	cold	polyphagous	larva	287
Noctuidae	*Ipimorpha retusa*	medium	intermediate	oligophagous	egg	913
Noctuidae	*Ipimorpha subtusa*	medium	intermediate	oligophagous	egg	744
Noctuidae	*Jodia croceago*	large	warm	monophagous	adult	8
Noctuidae	*Lacanobia aliena*	large	cold	polyphagous	pupa	95
Noctuidae	*Lacanobia contigua*	large	cold	polyphagous	pupa	798
Noctuidae	*Lacanobia oleracea*	large	intermediate	polyphagous	pupa	2703
Noctuidae	*Lacanobia splendens*	large	warm	polyphagous	pupa	1431
Noctuidae	*Lacanobia suasa*	large	cold	polyphagous	pupa	5889
Noctuidae	*Lacanobia thalassina*	large	cold	polyphagous	pupa	2528
Noctuidae	*Lacanobia w-latinum*	large	warm	polyphagous	pupa	2426
Noctuidae	*Lamprotes c-aureum*	large	cold	oligophagous	larva	79
Noctuidae	*Lasionhada proxima*	medium	cold	polyphagous	larva	7830
Noctuidae	*Lateroligia ophiogramma*	medium	intermediate	oligophagous	larva	679
Noctuidae	*Lenisa geminipuncta*	medium	intermediate	monophagous	egg	19
Noctuidae	*Leucania comma*	large	cold	oligophagous	larva	8176
Noctuidae	*Leucania loreyi*	large	warm	oligophagous	larva	44
Noctuidae	*Leucania obsoleta*	medium	intermediate	monophagous	larva	820
Noctuidae	*Lithophane consocia*	large	cold	oligophagous	adult	103
Noctuidae	*Lithophane furcifera*	large	cold	polyphagous	adult	127
Noctuidae	*Lithophane leautieri*	large	warm	oligophagous	adult	71
Noctuidae	*Lithophane ornitopus*	large	warm	polyphagous	adult	1022
Noctuidae	*Lithophane semibrunnea*	large	warm	monophagous	adult	23
Noctuidae	*Lithophane socia*	large	cold	polyphagous	adult	716
Noctuidae	*Litoligia literosa*	medium	cold	oligophagous	larva	806
Noctuidae	*Luperina dumerilii*	medium	warm	oligophagous	larva	1
Noctuidae	*Luperina testacea*	large	warm	oligophagous	larva	2633
Noctuidae	*Luteohadena luteago*	large	warm	oligophagous	pupa	1305
Noctuidae	*Lycophotia porphyrea*	medium	intermediate	oligophagous	larva	9041
Noctuidae	*Macdunnoughia confusa*	medium	warm	polyphagous	larva	1474
Noctuidae	*Mamestra brassicae*	large	intermediate	polyphagous	pupa	4298
Noctuidae	*Melanchra persicariae*	large	intermediate	polyphagous	pupa	4174
Noctuidae	*Mesapamea secalis* aggr.	medium	intermediate	oligophagous	larva	11795
Noctuidae	*Mesogona acetosellae*	large	warm	polyphagous	egg	3
Noctuidae	*Mesogona oxalina*	large	cold	polyphagous	egg	285
Noctuidae	*Mesoligia furuncula*	medium	intermediate	polyphagous	larva	1831
Noctuidae	*Mniotype adusta*	large	cold	polyphagous	larva	9212

Noctuidae	*Mniotype satura*	large	cold	polyphagous	egg	3332
Noctuidae	*Mniotype solieri*	large	warm	NA	larva	11
Noctuidae	*Moma alpium*	medium	intermediate	polyphagous	pupa	1160
Noctuidae	*Mormo maura*	large	warm	polyphagous	larva	32
Noctuidae	*Mythimna albipuncta*	medium	warm	oligophagous	larva	13268
Noctuidae	*Mythimna anderreggii*	medium	cold	oligophagous	pupa	10286
Noctuidae	*Mythimna congrua*	medium	warm	oligophagous	larva	6
Noctuidae	*Mythimna conigera*	medium	cold	oligophagous	larva	11184
Noctuidae	*Mythimna ferrago*	large	intermediate	oligophagous	larva	6925
Noctuidae	*Mythimna impura*	medium	intermediate	polyphagous	larva	4922
Noctuidae	*Mythimna l-album*	medium	warm	oligophagous	larva	5396
Noctuidae	*Mythimna pallens*	large	intermediate	oligophagous	larva	6605
Noctuidae	*Mythimna pudorina*	large	intermediate	polyphagous	larva	1531
Noctuidae	*Mythimna riparia*	medium	warm	oligophagous	larva	13
Noctuidae	*Mythimna sicula*	large	warm	oligophagous	larva	2529
Noctuidae	*Mythimna straminea*	large	intermediate	polyphagous	larva	645
Noctuidae	*Mythimna turca*	large	intermediate	polyphagous	larva	3209
Noctuidae	*Mythimna unipuncta*	large	warm	oligophagous	larva	3099
Noctuidae	*Mythimna vitellina*	large	warm	oligophagous	larva	1452
Noctuidae	*Naenia typica*	large	intermediate	polyphagous	larva	35
Noctuidae	*Noctua comes*	large	warm	polyphagous	larva	5002
Noctuidae	*Noctua fimbriata*	large	intermediate	polyphagous	larva	7735
Noctuidae	*Noctua interjecta*	medium	warm	polyphagous	larva	421
Noctuidae	*Noctua interposita*	large	intermediate	polyphagous	larva	13
Noctuidae	*Noctua janthe/janthina* aggr.	large	warm	polyphagous	larva	9771
Noctuidae	*Noctua orbona*	large	intermediate	polyphagous	larva	22
Noctuidae	*Noctua pronuba*	large	intermediate	polyphagous	larva	123954
Noctuidae	*Noctua tirrenica*	large	warm	polyphagous	larva	6
Noctuidae	*Nonagria typhae*	large	intermediate	monophagous	egg	91
Noctuidae	*Ochropleura plecta*	medium	intermediate	polyphagous	pupa	43114
Noctuidae	*Oligia dubia*	medium	warm	NA	larva	159
Noctuidae	*Oligia latruncula*	medium	intermediate	oligophagous	larva	9271
Noctuidae	*Oligia strigilis*	medium	intermediate	oligophagous	larva	22459
Noctuidae	*Oligia versicolor*	medium	warm	polyphagous	larva	13809
Noctuidae	*Opigena polygona*	large	cold	polyphagous	larva	226
Noctuidae	*Orbona fragariae*	large	cold	polyphagous	adult	12
Noctuidae	*Orthosia cerasi*	large	warm	polyphagous	pupa	45714
Noctuidae	*Orthosia cruda*	medium	warm	polyphagous	pupa	14815
Noctuidae	*Orthosia gothica*	large	intermediate	polyphagous	pupa	27820
Noctuidae	*Orthosia gracilis*	large	intermediate	polyphagous	pupa	488
Noctuidae	*Orthosia incerta*	large	intermediate	polyphagous	pupa	7399
Noctuidae	*Orthosia miniosa*	medium	warm	polyphagous	pupa	14
Noctuidae	*Orthosia opima*	large	cold	polyphagous	pupa	40
Noctuidae	*Orthosia populeti*	large	cold	monophagous	pupa	288
Noctuidae	*Pachetra sagittigera*	large	intermediate	polyphagous	larva	2688
Noctuidae	*Panchrysia deaurata*	large	cold	monophagous	larva	27
Noctuidae	*Panchrysia v-argenteum*	large	cold	monophagous	larva	60
Noctuidae	*Panemeria tenebrata*	medium	warm	oligophagous	pupa	6
Noctuidae	*Panolis flammea*	large	intermediate	oligophagous	pupa	825
Noctuidae	*Panthea coenobita*	large	cold	oligophagous	pupa	770
Noctuidae	*Papestra biren*	medium	cold	polyphagous	pupa	10888
Noctuidae	*Paradiarsia punicea*	medium	cold	polyphagous	larva	90
Noctuidae	*Parastichtis suspecta*	medium	cold	polyphagous	egg	85
Noctuidae	*Peridroma saucia*	large	warm	polyphagous	larva	734
Noctuidae	*Phlogophora meticulosa*	large	warm	polyphagous	larva	13445
Noctuidae	*Phlogophora scita*	large	cold	polyphagous	larva	249
Noctuidae	*Photedes captiuncula*	medium	cold	polyphagous	larva	7
Noctuidae	*Photedes fluxa*	medium	cold	monophagous	larva	4
Noctuidae	*Photedes minima*	medium	cold	monophagous	larva	119
Noctuidae	*Photedes morrisii*	medium	warm	monophagous	larva	1
Noctuidae	*Phragmatiphila nexa*	medium	intermediate	polyphagous	egg/larva	9
Noctuidae	*Phyllophila obliterata*	medium	warm	monophagous	pupa	711
Noctuidae	*Plusia festucae*	large	intermediate	polyphagous	larva	142
Noctuidae	*Polia bombycina*	large	cold	polyphagous	larva	3292
Noctuidae	*Polia hepatica*	large	cold	polyphagous	larva	180
Noctuidae	*Polia nebulosa*	large	intermediate	polyphagous	larva	1517
Noctuidae	*Polia serratilinea*	large	cold	polyphagous	larva	62
Noctuidae	*Polychrysia moneta*	large	cold	oligophagous	larva	190
Noctuidae	*Polymixis rufocincta*	large	warm	polyphagous	egg	228
Noctuidae	*Polymixis xanthomista*	large	warm	polyphagous	egg	643
Noctuidae	*Polyphaenis sericata*	large	warm	polyphagous	larva	837
Noctuidae	*Protolampra sobrina*	large	cold	polyphagous	larva	275
Noctuidae	*Pseudeustrotia candidula*	medium	intermediate	polyphagous	pupa	794
Noctuidae	*Pyrrhia umbra*	medium	intermediate	polyphagous	pupa	890
Noctuidae	*Rhizedra lutosa*	large	intermediate	monophagous	egg	185
Noctuidae	*Rhyacia helvetina*	large	cold	polyphagous	larva	602
Noctuidae	*Rhyacia lucipeta*	large	cold	polyphagous	larva	30
Noctuidae	*Rhyacia simulans*	large	intermediate	polyphagous	larva	131

Noctuidae	*Rusina ferruginea*	large	intermediate	polyphagous	larva	4860
Noctuidae	*Sedina buettneri*	medium	intermediate	polyphagous	egg	151
Noctuidae	*Sideridis kitti*	large	cold	oligophagous	pupa	180
Noctuidae	*Sideridis lampra*	medium	cold	polyphagous	larva	42
Noctuidae	*Sideridis reticulata*	large	cold	NA	pupa	1814
Noctuidae	*Sideridis rivularis*	medium	intermediate	oligophagous	pupa	1838
Noctuidae	*Sideridis turbida*	large	warm	polyphagous	pupa	51
Noctuidae	*Spaelotis ravida*	large	cold	polyphagous	larva	1
Noctuidae	*Spaelotis senna*	large	cold	polyphagous	larva	14
Noctuidae	*Spodoptera exigua*	medium	warm	polyphagous	larva	1417
Noctuidae	*Standfussiana lucernea*	large	cold	polyphagous	larva	646
Noctuidae	*Standfussiana wiskotti*	large	cold	polyphagous	larva	10
Noctuidae	*Staurophora celsia*	large	cold	oligophagous	egg	19
Noctuidae	*Sunira circellaris*	large	intermediate	polyphagous	egg	13144
Noctuidae	*Syngrapha ain*	large	cold	monophagous	larva	355
Noctuidae	*Syngrapha hochenwarthi*	medium	cold	polyphagous	larva	17
Noctuidae	*Syngrapha interrogationis*	large	cold	oligophagous	larva	353
Noctuidae	*Thalpophila matura*	large	warm	oligophagous	larva	3565
Noctuidae	*Tholera cespitis*	large	cold	oligophagous	larva	560
Noctuidae	*Tholera decimalis*	large	intermediate	oligophagous	egg	3865
Noctuidae	*Tiliacea aurago*	medium	intermediate	polyphagous	egg	3940
Noctuidae	*Tiliacea citrago*	medium	intermediate	monophagous	egg	1213
Noctuidae	*Trachea atriplicis*	large	intermediate	polyphagous	pupa	1862
Noctuidae	*Trichoplusia ni*	large	warm	polyphagous	larva	19
Noctuidae	*Trigonophora flammea*	large	warm	polyphagous	larva	4065
Noctuidae	*Tyta luctuosa*	medium	warm	monophagous	pupa	92
Noctuidae	*Xanthia togata*	medium	cold	polyphagous	egg	669
Noctuidae	*Xestia alpicola*	large	cold	polyphagous	larva	85
Noctuidae	*Xestia ashworthii*	large	cold	polyphagous	larva	1130
Noctuidae	*Xestia baja*	large	cold	polyphagous	larva	7203
Noctuidae	*Xestia c-nigrum*	large	intermediate	polyphagous	larva	100916
Noctuidae	*Xestia castanea*	large	intermediate	polyphagous	larva	549
Noctuidae	*Xestia collina*	medium	cold	polyphagous	larva	41
Noctuidae	*Xestia ditrapezium*	large	intermediate	polyphagous	larva	8501
Noctuidae	*Xestia lorezi*	large	cold	polyphagous	larva	26
Noctuidae	*Xestia ochreago*	large	cold	polyphagous	larva	2153
Noctuidae	*Xestia rhaetica*	large	cold	monophagous	larva	46
Noctuidae	*Xestia sexstrigata*	large	intermediate	polyphagous	larva	127
Noctuidae	*Xestia speciosa*	large	cold	polyphagous	larva	909
Noctuidae	*Xestia stigmatica*	large	intermediate	polyphagous	larva	2216
Noctuidae	*Xestia triangulum*	large	intermediate	polyphagous	larva	3511
Noctuidae	*Xestia xanthographa*	medium	warm	polyphagous	larva	15026
Noctuidae	*Xylena exsoleta*	large	warm	polyphagous	adult	1
Noctuidae	*Xylena solidaginis*	large	cold	polyphagous	egg	32
Noctuidae	*Xylena vetusta*	large	cold	polyphagous	adult	141
Noctuidae	*Xylocampa areola*	large	warm	monophagous	pupa	4
Nolidae	*Bena bicolorana*	large	warm	monophagous	larva	812
Nolidae	*Earias biplaga*	small	warm	NA	NA	1
Nolidae	*Earias clorana*	small	intermediate	monophagous	pupa	1714
Nolidae	*Earias vernana*	small	warm	monophagous	pupa	57
Nolidae	*Meganola albula*	small	warm	polyphagous	larva	650
Nolidae	*Meganola strigula*	small	warm	polyphagous	larva	581
Nolidae	*Meganola togatulalis*	medium	warm	monophagous	larva	1
Nolidae	*Nola aerugula*	small	cold	polyphagous	larva	1599
Nolidae	*Nola confusalis*	small	intermediate	polyphagous	pupa	3435
Nolidae	*Nola cucullatella*	small	intermediate	oligophagous	larva	30
Nolidae	*Nola subchlamydula*	small	warm	oligophagous	pupa	76
Nolidae	*Nycteola asiatica*	medium	intermediate	oligophagous	adult	81
Nolidae	*Nycteola degenerana*	medium	cold	monophagous	adult	83
Nolidae	*Nycteola revayana*	medium	warm	monophagous	adult	299
Nolidae	*Nycteola siculana*	medium	warm	oligophagous	adult	3
Nolidae	*Pseudoips prasinana*	medium	intermediate	polyphagous	pupa	5763
Notodontidae	*Cerura erminea*	large	warm	monophagous	pupa	26
Notodontidae	*Cerura vinula*	large	intermediate	oligophagous	pupa	54
Notodontidae	*Clostera anachoreta*	medium	intermediate	oligophagous	pupa	17
Notodontidae	*Clostera anastomosis*	medium	cold	oligophagous	larva	4
Notodontidae	*Clostera curtula*	medium	intermediate	oligophagous	pupa	397
Notodontidae	*Clostera pigra*	medium	cold	polyphagous	pupa	2473
Notodontidae	*Drymonia dodonaea*	medium	warm	monophagous	pupa	1589
Notodontidae	*Drymonia obliterata*	medium	warm	oligophagous	pupa	828
Notodontidae	*Drymonia querna*	large	warm	oligophagous	pupa	493
Notodontidae	*Drymonia ruficornis*	large	warm	monophagous	pupa	656
Notodontidae	*Drymonia velitaris*	large	warm	monophagous	pupa	3
Notodontidae	*Furcula bicuspis*	large	intermediate	polyphagous	pupa	100
Notodontidae	*Furcula bifida*	large	intermediate	oligophagous	pupa	79
Notodontidae	*Furcula furcula*	medium	intermediate	polyphagous	pupa	193
Notodontidae	*Gluphisia crenata*	medium	intermediate	monophagous	pupa	583
Notodontidae	*Harpyia milhauseri*	large	warm	polyphagous	pupa	126

Notodontidae	*Leucodonta bicoloria*	medium	cold	monophagous	pupa	136
Notodontidae	*Notodonta dromedarius*	large	intermediate	polyphagous	pupa	1710
Notodontidae	*Notodonta tritophus*	large	intermediate	oligophagous	pupa	87
Notodontidae	*Notodonta ziczac*	large	intermediate	polyphagous	pupa	705
Notodontidae	*Odontosia carmelita*	large	cold	oligophagous	pupa	177
Notodontidae	*Peridea anceps*	large	warm	monophagous	pupa	2092
Notodontidae	*Phalera bucephala*	large	intermediate	polyphagous	pupa	3086
Notodontidae	*Pheosia gnoma*	large	cold	polyphagous	pupa	2018
Notodontidae	*Pheosia tremula*	large	intermediate	oligophagous	pupa	979
Notodontidae	*Pterostoma palpina*	large	intermediate	oligophagous	pupa	2098
Notodontidae	*Ptilodon capucina*	large	intermediate	polyphagous	pupa	3550
Notodontidae	*Ptilodon cucullina*	large	warm	monophagous	pupa	1734
Notodontidae	*Ptilophora plumigera*	large	intermediate	monophagous	egg	1827
Notodontidae	*Spatalia argentina*	large	warm	monophagous	pupa	420
Notodontidae	*Stauropus fagi*	large	warm	polyphagous	pupa	2163
Notodontidae	*Thaumetopoea pityocampa*	large	warm	monophagous	larva	634
Notodontidae	*Thaumetopoea processionea*	medium	warm	monophagous	egg	1254
Saturniidae	*Aglia tau*	large	intermediate	polyphagous	pupa	354
Saturniidae	*Saturnia pavonia*	large	intermediate	polyphagous	pupa	28
Saturniidae	*Saturnia pavoniella*	large	warm	polyphagous	pupa	88
Saturniidae	*Saturnia pyri*	large	warm	oligophagous	pupa	79
Sphingidae	*Acherontia atropos*	large	warm	polyphagous	pupa	66
Sphingidae	*Agrius convolvuli*	large	warm	oligophagous	pupa	1958
Sphingidae	*Deilephila elpenor*	large	intermediate	polyphagous	pupa	1313
Sphingidae	*Deilephila porcellus*	large	intermediate	monophagous	pupa	3001
Sphingidae	*Hyles euphorbiae*	large	warm	monophagous	pupa	174
Sphingidae	*Hyles gallii*	large	cold	polyphagous	pupa	24
Sphingidae	*Hyles livornica*	large	warm	polyphagous	pupa	130
Sphingidae	*Hyles vespertilio*	large	intermediate	monophagous	pupa	8
Sphingidae	*Laothoe populi*	large	intermediate	oligophagous	pupa	2528
Sphingidae	*Mimas tiliae*	large	warm	polyphagous	pupa	1342
Sphingidae	*Proserpinus proserpina*	large	warm	oligophagous	pupa	3
Sphingidae	*Smerinthus ocellata*	large	intermediate	polyphagous	pupa	226
Sphingidae	*Sphinx ligustri*	large	intermediate	polyphagous	pupa	368
Sphingidae	*Sphinx pinastri*	large	intermediate	oligophagous	pupa	2740

Table S2 Detailed model results for the model analysing the effect of year, elevation, their interaction (and co-variates) on moth abundance, richness, and biomass. Fixed effects of linear effects and splines and standard deviations of random factors and splines are given. For factors, sum-to-zero contrasts were used. Point estimates and 95% credible intervals (95%-CI) are given. If 95%-CI do not include zero, numbers are bold.

Para-meter	Type	Variable	Abundance			Richness			Biomass		
			**Estimate**	**Lower 95%-CI**	**Upper 95%-CI**	**Estimate**	**Lower 95%-CI**	**Upper 95%-CI**	**Estimate**	**Lower 95%-CI**	**Upper 95%-CI**
Fixed effect	Fixed	Year	-0.0538	-0.140	0.0322	-0.0201	-0.0760	0.0361	-0.0253	-0.108	0.0582
		Elevation	**0.418**	**0.299**	**0.538**	-0.0225	-0.101	0.0553	**0.458**	**0.336**	**0.578**
		Precipitation	**0.0142**	**0.00224**	**0.0261**	-0.000778	-0.0104	0.00885	**0.0207**	**0.00914**	**0.0323**
		Temperature	**0.730**	**0.706**	**0.754**	**0.578**	**0.559**	**0.597**	**0.525**	**0.502**	**0.549**
		Trap type (contr. sum 1)	**-0.526**	**-0.890**	**-0.164**	**-0.382**	**-0.613**	**-0.150**	**-0.355**	**-0.706**	**-0.00999**
		Trap type (contr. sum 2)	**-0.674**	**-1.32**	**-0.0218**	-0.135	-0.550	0.286	**-0.743**	**-1.41**	**-0.110**
		Lamp type (contr. sum 1)	-0.146	-0.462	0.165	-0.0460	-0.268	0.176	-0.179	-0.496	0.147
		Lamp type (contr. sum 2)	0.208	-0.442	0.857	0.0609	-0.443	0.550	0.0105	-0.650	0.674
		Lamp type (contr. sum 3)	-0.243	-0.570	0.0709	0.00760	-0.213	0.230	-0.286	-0.604	0.0353
		Nr. of traps (linear)	0.418	-0.596	1.41	0.369	-0.346	1.09	0.334	-0.632	1.32
		Nr. of traps (quad-ratic)	-0.446	-1.24	0.388	-0.340	-0.885	0.202	-0.551	-1.32	0.214
		Nr. of traps (cubic)	-0.204	-0.588	0.188	-0.0147	-0.302	0.274	-0.285	-0.660	0.0830
		Sampl. prev. night (contr. sum 1)	**-0.104**	**-0.169**	**-0.0424**	**-0.0876**	**-0.135**	**-0.0392**	**-0.129**	**-0.190**	**-0.0680**
		Year × Elevation	0.0476	-0.0212	0.115	**0.0680**	**0.0202**	**0.114**	0.0428	-0.0206	0.108
	Spline	Day of year	**-23.3**	**-27.8**	**-18.8**	**-3.55**	**-6.99**	**-0.309**	**-18.7**	**-23.0**	**-14.5**
		Hours active	**6.63**	**1.82**	**11.3**	2.41	-0.400	5.53	**5.57**	**0.849**	**10.1**
SD	Random	Spat-temp. cluster	0.257	0.189	0.326	0.119	0.0684	0.162	0.269	0.202	0.333
		Sampling night × Location	0.794	0.771	0.816	0.367	0.346	0.388	0.598	0.577	0.619
		Site ID	0.597	0.524	0.676	0.390	0.339	0.447	0.556	0.485	0.633
		Site ID × Year	0.239	0.200	0.284	0.143	0.118	0.172	0.228	0.191	0.271
	Spline	Day of year	12.6	8.27	19.7	4.09	2.60	6.68	11.3	7.37	17.8
		Hours active	1.88	0.906	3.66	0.907	0.270	2.12	1.85	0.837	3.70

Nr. of traps: Number of traps
Sampl. prev. night: Sampling in previous night
Spat.-temp. cluster: Spatio-temporal cluster

Table S3 Change in total biomass across the 50 study years based on model predictions in dependence of elevation and for different species groups defined by traits. For three different elevations (lowest, median, highest), the change in the prediction from the first to the last study year is given, once as a factor and once as percentage change. Numbers are means and 95%-credible intervals (CIs). The threshold elevation indicates the elevation at which the model terms for year and for the interactions between year and elevation cancel each other out, resulting in no predicted change across years for that respective elevation. Above and below the threshold, the model predicts yearly changes in opposite directions. Mean and 95%-CIs are given for the thresholds. Threshold elevations that are outside of the elevational range studied here are simplified to "<min" (below lowest site) and ">max" (above highest site). Red indicates decreases with CIs not including no change, blue indicates increases with CIs not including no change.

Trait	Trait-value	Elevation: lowest	Elevation: median	Elevation: highest	Threshold elevation (m asl)
Full data		0.711 (0.396–1.21)<br>-28.9% (-60.4%–+20.6%)	1.13 (0.614–1.92)<br>+12.6% (-38.6%–+92.0%)	2.03 (0.568–5.44)<br>+103% (-43.2%–+444%)	279<br>(<min – >max)
Body size	small	0.409 (0.225–0.680)<br>-59.1% (-77.5%–-32.0%)	0.619 (0.323–1.09)<br>-38.1% (-67.7%–+8.82%)	1.09 (0.252–3.06)<br>+8.52% (-74.8%–+206%)	>max<br>(<min – >max)
Body size	medium	0.669 (0.371–1.13)<br>-33.1% (-62.9%–+12.9%)	1.31 (0.721–2.24)<br>+31.3% (-27.9%–+124%)	2.94 (0.779–7.89)<br>+194% (-22.1%–+689%)	1326<br>(<min – >max)
Body size	large	0.643 (0.352–1.11)<br>-35.7% (-64.8%–+11.2%)	1.25 (0.674–2.17)<br>+25.0% (-32.6%–+117%)	2.79 (0.706–7.81)<br>+179% (-29.4%–+681%)	915<br>(<min – >max)
Temperature niche	cold	0.141 (0.0734–0.244)<br>-85.9% (-92.7%–-75.6%)	0.823 (0.442–1.42)<br>-17.7% (-55.8%–+41.7%)	5.57 (1.43–15.4)<br>+457% (+43.4%–+1438%)	1513<br>(1145 – 2086)
Temperature niche	interm.	0.562 (0.310–0.933)<br>-43.8% (-69.0%–-6.74%)	1.22 (0.684–2.04)<br>+21.6% (-31.6%–+104%)	2.98 (0.826–7.82)<br>+198% (-17.4%–+682%)	>max<br>(238 – >max)
Temperature niche	warm	2.09 (0.972–4.05)<br>+109% (-2.85%–+305%)	0.805 (0.367–1.58)<br>-19.5% (-63.3%–+58.3%)	0.397 (0.0641–1.49)<br>-60.3% (-93.6%–+48.9%)	1285<br>(<min – >max)
Food specialisation	monoph.	0.585 (0.304–1.04)<br>-41.5% (-69.6%–+4.28%)	1.07 (0.535–1.94)<br>+6.56% (-46.5%–+94.0%)	2.31 (0.500–7.17)<br>+131% (-50.0%–+617%)	2338<br>(<min – >max)
Food specialisation	oligoph.	0.488 (0.247–0.838)<br>-51.2% (-75.3%–-16.2%)	1.12 (0.571–2.01)<br>+12.0% (-42.9%–+101%)	3.02 (0.717–8.92)<br>+202% (-28.3%–+792%)	1286<br>(348 – >max)
Food specialisation	polyph.	0.742 (0.413–1.24)<br>-25.8% (-58.7%–+23.9%)	1.11 (0.610–1.88)<br>+11.3% (-39.0%–+88.1%)	1.91 (0.497–5.27)<br>+91.4% (-50.3%–+427%)	1080<br>(<min – >max)
Overwintering stage	egg	0.895 (0.454–1.59)<br>-10.5% (-54.6%–+59.2%)	1.70 (0.829–2.98)<br>+70.1% (-17.1%–+198%)	3.81 (0.761–10.9)<br>+281% (-23.9%–+995%)	479<br>(<min – >max)
Overwintering stage	larva	0.690 (0.351–1.19)<br>-31.0% (-64.9%–+18.9%)	0.879 (0.446–1.52)<br>-12.1% (-55.4%–+51.6%)	1.29 (0.305–3.55)<br>+28.7% (-69.5%–+255%)	<min<br>(<min – >max)
Overwintering stage	pupa	0.740 (0.390–1.26)<br>-26.0% (-61.0%–+26.5%)	1.90 (0.987–3.31)<br>+90.3% (-1.30%–+231%)	5.67 (1.37–16.0)<br>+467% (+36.8%–+1501%)	563<br>(<min – 1368)
Overwintering stage	adult	0.675 (0.407–1.07)<br>-32.5% (-59.3%–+7.50%)	0.530 (0.313–0.854)<br>-47.0% (-68.7%–-14.6%)	0.461 (0.142–1.12)<br>-53.9% (-85.8%–+12.1%)	<min<br>(<min – >max)

Table S4 Detailed model results for the model analysing the effect of year, elevation, their interaction (and co-variates) on moth abundance for different body size categories (small, medium, large). Fixed effects of linear effects and splines and standard deviations of random factors and splines are given. For factors, sum-to-zero contrasts were used. Point estimates and 95% credible intervals (95%-CI) are given. If 95%-CI do not include zero, numbers are bold.

Para-meter	Type	Variable	Small			Medium			Large		
			Estimate	Lower 95%-CI	Upper 95%-CI	Estimate	Lower 95%-CI	Upper 95%-CI	Estimate	Lower 95%-CI	Upper 95%-CI
Fixed effect	Fixed	Year	**-0.119**	**-0.227**	**-0.00699**	-0.0117	-0.104	0.0842	-0.0312	-0.122	0.0573
		Elevation	**0.235**	**0.0572**	**0.394**	**0.226**	**0.0725**	**0.374**	**0.515**	**0.393**	**0.643**
		Precipitation	0.00640	-0.0111	0.0225	**0.0231**	**0.00948**	**0.0368**	0.0102	-0.00329	0.0239
		Temperature	**1.18**	**1.14**	**1.22**	**0.939**	**0.911**	**0.969**	**0.552**	**0.524**	**0.581**
		Trap type (contr. sum 1)	**-1.34**	**-1.86**	**-0.855**	**-0.707**	**-1.17**	**-0.280**	-0.182	-0.564	0.194
		Trap type (contr. sum 2)	-0.335	-1.24	0.567	-0.237	-1.01	0.604	**-0.933**	**-1.65**	**-0.219**
		Lamp type (contr. sum 1)	-0.184	-0.606	0.226	-0.0544	-0.432	0.336	-0.198	-0.553	0.153
		Lamp type (contr. sum 2)	0.510	-0.274	1.31	-0.0468	-0.760	0.702	-0.114	-0.848	0.610
		Lamp type (contr. sum 3)	-0.146	-0.571	0.265	-0.173	-0.557	0.216	-0.338	-0.705	0.0158
		Nr. of traps (linear)	0.416	-1.01	1.84	0.917	-0.334	2.24	0.342	-0.771	1.44
		Nr. of traps (quad-ratic)	-0.176	-1.30	0.940	-0.556	-1.55	0.430	-0.513	-1.40	0.297
		Nr. of traps (cubic)	0.0399	-0.507	0.578	-0.115	-0.610	0.364	-0.238	-0.662	0.180
		Sampl. prev. night (contr. sum 1)	**-0.194**	**-0.296**	**-0.0899**	-0.0480	-0.128	0.0284	-0.0537	-0.127	0.0197
		Year × Elevation	0.0752	-0.0207	0.165	**0.0894**	**0.0106**	**0.172**	**0.0810**	**0.00783**	**0.153**
	Spline	Day of year	**15.6**	**7.61**	**23.3**	**-17.4**	**-22.7**	**-12.3**	**-21.5**	**-28.2**	**-14.7**
		Hours active	**5.38**	**0.971**	**9.83**	**6.87**	**2.03**	**11.8**	4.19	-0.708	8.57
SD	Random	Spat-temp. cluster	0.153	0.0333	0.244	0.186	0.0920	0.265	0.332	0.259	0.403
		Sampling night × Location	1.06	1.04	1.08	0.920	0.902	0.939	0.876	0.859	0.893
		Site ID	0.889	0.786	1.01	0.774	0.681	0.874	0.606	0.528	0.692
		Site ID × Year	0.254	0.206	0.304	0.253	0.213	0.296	0.260	0.216	0.307
	Spline	Day of year	12.7	8.10	20.2	11.7	7.64	18.3	17.7	11.5	28.1
		Hours active	1.50	0.611	3.15	1.94	0.901	3.92	1.80	0.694	3.76

Nr. of traps: Number of traps
Sampl. prev. night: Sampling in previous night
Spat.-temp. cluster: Spatio-temporal cluster

Table S5 Detailed model results for the model analysing the effect of year, elevation, their interaction (and co-variates) on moth richness for different body size categories (small, medium, large). Fixed effects of linear effects and splines and standard deviations of random factors and splines are given. For factors, sum-to-zero contrasts were used. Point estimates and 95% credible intervals (95%-CI) are given. If 95%-CI do not include zero, numbers are bold.

Para-meter	Type	Variable	Small			Medium			Large		
			Estimate	Lower 95%-CI	Upper 95%-CI	Estimate	Lower 95%-CI	Upper 95%-CI	Estimate	Lower 95%-CI	Upper 95%-CI
Fixed effect	Fixed	Year	-0.0288	-0.0921	0.0354	-0.0285	-0.0907	0.0301	-0.0268	-0.0844	0.0314
		Elevation	-0.0497	-0.136	0.0360	**-0.109**	**-0.196**	**-0.0235**	**0.115**	**0.0340**	**0.199**
		Precipitation	0.00319	-0.00958	0.0158	0.00309	-0.00676	0.0131	-0.00580	-0.0153	0.00391
		Temperature	**0.603**	**0.577**	**0.630**	**0.552**	**0.531**	**0.572**	**0.423**	**0.404**	**0.442**
		Trap type (contr. sum 1)	**-0.540**	**-0.800**	**-0.277**	**-0.436**	**-0.716**	**-0.169**	-0.187	-0.441	0.0582
		Trap type (contr. sum 2)	-0.0251	-0.503	0.456	0.108	-0.354	0.590	-0.212	-0.650	0.247
		Lamp type (contr. sum 1)	-0.0843	-0.321	0.159	-0.0140	-0.252	0.224	-0.0423	-0.273	0.181
		Lamp type (contr. sum 2)	0.237	-0.272	0.751	0.0489	-0.467	0.560	-0.0749	-0.598	0.423
		Lamp type (contr. sum 3)	0.0625	-0.185	0.315	-0.00201	-0.237	0.235	-0.0794	-0.316	0.152
		Nr. of traps (linear)	0.191	-0.620	0.966	0.492	-0.301	1.30	0.544	-0.183	1.29
		Nr. of traps (quad-ratic)	-0.301	-0.901	0.291	-0.309	-0.941	0.302	-0.311	-0.900	0.278
		Nr. of traps (cubic)	-0.0561	-0.348	0.241	0.0129	-0.276	0.309	-0.0400	-0.325	0.243
		Sampl. prev. night (contr. sum 1)	-0.0281	-0.106	0.0484	-0.0201	-0.0751	0.0368	-0.00799	-0.0559	0.0421
		Year × Elevation	**0.0597**	**0.0103**	**0.109**	**0.0570**	**0.00854**	**0.106**	**0.0698**	**0.0239**	**0.118**
	Spline	Day of year	-1.20	-7.45	4.59	**-8.07**	**-11.9**	**-4.34**	0.391	-4.49	5.17
		Hours active	0.455	-4.97	5.69	2.53	-1.68	6.88	2.51	-1.29	6.16
SD	Random	Spat-temp. cluster	0.0878	0.0145	0.144	0.0764	0.0125	0.130	0.142	0.0952	0.187
		Sampling night × Location	0.656	0.636	0.674	0.520	0.499	0.540	0.484	0.464	0.505
		Site ID	0.449	0.393	0.514	0.462	0.409	0.524	0.409	0.356	0.466
		Site ID × Year	0.131	0.0976	0.164	0.151	0.125	0.177	0.151	0.125	0.179
	Spline	Day of year	4.12	2.52	6.91	5.62	3.56	9.12	4.18	2.69	6.76
		Hours active	2.98	1.33	5.66	1.83	0.828	3.54	1.42	0.501	3.20

Nr. of traps: Number of traps
Sampl. prev. night: Sampling in previous night
Spat.-temp. cluster: Spatio-temporal cluster

Table S6 Detailed model results for the model analysing the effect of year, elevation, their interaction (and co-variates) on moth biomass for different body size categories (small, medium, large). Fixed effects of linear effects and splines and standard deviations of random factors and splines are given. For factors, sum-to-zero contrasts were used. Point estimates and 95% credible intervals (95%-CI) are given. If 95%-CI do not include zero, numbers are bold.

Para-meter	Type	Variable	Small			Medium			Large		
			**Estimate**	**Lower 95%-CI**	**Upper 95%-CI**	**Estimate**	**Lower 95%-CI**	**Upper 95%-CI**	**Estimate**	**Lower 95%-CI**	**Upper 95%-CI**
Fixed effect	Fixed	Year	**-0.134**	**-0.215**	**-0.0506**	-0.0146	-0.0962	0.0647	-0.0239	-0.109	0.0646
		Elevation	**0.280**	**0.150**	**0.410**	**0.225**	**0.100**	**0.350**	**0.471**	**0.358**	**0.591**
		Precipitation	0.00783	-0.00604	0.0217	**0.0242**	**0.0121**	**0.0370**	**0.0184**	**0.00627**	**0.0303**
		Temperature	**0.654**	**0.625**	**0.683**	**0.609**	**0.585**	**0.634**	**0.402**	**0.378**	**0.427**
		Trap type (contr. sum 1)	**-0.832**	**-1.22**	**-0.458**	**-0.475**	**-0.843**	**-0.112**	-0.233	-0.588	0.131
		Trap type (contr. sum 2)	-0.242	-0.924	0.472	-0.127	-0.770	0.524	**-0.796**	**-1.43**	**-0.151**
		Lamp type (contr. sum 1)	-0.143	-0.460	0.184	-0.0898	-0.404	0.224	-0.177	-0.490	0.139
		Lamp type (contr. sum 2)	0.436	-0.239	1.05	0.103	-0.566	0.769	-0.140	-0.809	0.529
		Lamp type (contr. sum 3)	-0.125	-0.447	0.206	-0.180	-0.493	0.146	-0.316	-0.631	0.00275
		Nr. of traps (linear)	0.364	-0.729	1.47	0.645	-0.375	1.69	0.382	-0.658	1.42
		Nr. of traps (quad-ratic)	-0.289	-1.14	0.554	-0.490	-1.29	0.342	-0.481	-1.25	0.316
		Nr. of traps (cubic)	-0.0384	-0.455	0.380	-0.202	-0.595	0.204	-0.253	-0.641	0.142
		Sampl. prev. night (contr. sum 1)	-0.0713	-0.160	0.0127	-0.00179	-0.0704	0.0638	**-0.0878**	**-0.154**	**-0.0227**
		Year × Elevation	0.0381	-0.0317	0.108	0.0628	-0.00489	0.127	0.0623	-0.00762	0.132
	Spline	Day of year	**7.07**	**0.0812**	**14.3**	**-12.2**	**-16.9**	**-7.69**	**-10.6**	**-17.0**	**-3.92**
		Hours active	4.20	-1.99	9.64	**6.04**	**0.233**	**11.7**	3.05	-2.31	7.62
SD	Random	Spat-temp. cluster	0.116	0.0245	0.187	0.155	0.0809	0.216	0.297	0.232	0.361
		Sampling night × Location	0.736	0.715	0.757	0.692	0.666	0.716	0.771	0.750	0.791
		Site ID	0.684	0.603	0.777	0.635	0.557	0.722	0.562	0.491	0.641
		Site ID × Year	0.191	0.149	0.234	0.207	0.174	0.244	0.217	0.181	0.259
	Spline	Day of year	7.98	4.88	13.1	9.28	6.14	14.8	10.6	6.94	16.6
		Hours active	3.02	1.42	5.52	3.35	1.63	6.14	1.80	0.678	3.79

Nr. of traps: Number of traps
Sampl. prev. night: Sampling in previous night
Spat.-temp. cluster: Spatio-temporal cluster

Table S7 Detailed model results for the model analysing the effect of year, elevation, their interaction (and co-variates) on moth abundance for different temperature niche categories (cold, intermediate, warm). Fixed effects of linear effects and splines and standard deviations of random factors and splines are given. For factors, sum-to-zero contrasts were used. Point estimates and 95% credible intervals (95%-CI) are given. If 95%-CI do not include zero, numbers are bold.

Para-meter	Type	Variable	Cold			Intermediate			Warm		
			**Estimate**	**Lower 95%-CI**	**Upper 95%-CI**	**Estimate**	**Lower 95%-CI**	**Upper 95%-CI**	**Estimate**	**Lower 95%-CI**	**Upper 95%-CI**
Fixed effect	Fixed	Year	**-0.211**	**-0.313**	**-0.111**	-0.0334	-0.123	0.0552	0.0456	-0.0837	0.178
		Elevation	**1.04**	**0.889**	**1.19**	**0.128**	**0.00276**	**0.260**	**-0.208**	**-0.392**	**-0.0149**
		Precipitation	0.0109	-0.00599	0.0277	-0.00424	-0.0169	0.00822	0.00430	-0.0118	0.0208
		Temperature	**0.896**	**0.863**	**0.929**	**0.725**	**0.698**	**0.753**	**0.761**	**0.729**	**0.793**
		Trap type (contr. sum 1)	**-0.750**	**-1.24**	**-0.282**	**-0.402**	**-0.779**	**-0.0422**	-0.551	-1.15	0.0195
		Trap type (contr. sum 2)	-0.447	-1.31	0.399	-0.566	-1.23	0.114	-0.726	-1.80	0.351
		Lamp type (contr. sum 1)	-0.0294	-0.426	0.380	-0.132	-0.478	0.220	-0.139	-0.626	0.356
		Lamp type (contr. sum 2)	0.282	-0.534	1.11	0.193	-0.523	0.906	0.514	-0.458	1.44
		Lamp type (contr. sum 3)	-0.172	-0.581	0.242	-0.245	-0.590	0.113	-0.244	-0.742	0.265
		Nr. of traps (linear)	0.925	-0.370	2.29	0.511	-0.548	1.62	-0.0817	-1.61	1.51
		Nr. of traps (quad-ratic)	-0.257	-1.27	0.779	-0.427	-1.26	0.411	-0.354	-1.58	0.924
		Nr. of traps (cubic)	0.0732	-0.404	0.573	-0.210	-0.611	0.212	-0.410	-1.02	0.172
		Sampl. prev. night (contr. sum 1)	**-0.0950**	**-0.187**	**-0.00647**	0.00842	-0.0639	0.0793	-0.0874	-0.178	0.00704
		Year × Elevation	**0.152**	**0.0683**	**0.238**	**0.0840**	**0.00776**	**0.152**	-0.0348	-0.147	0.0729
	Spline	Day of year	7.98	-0.722	16.9	**-21.3**	**-26.2**	**-16.6**	**-20.1**	**-27.9**	**-12.2**
		Hours active	4.75	-1.25	10.6	**6.40**	**1.59**	**10.9**	5.64	-0.166	11.9
SD	Random	Spat-temp. cluster	0.180	0.0531	0.265	0.324	0.242	0.401	0.408	0.309	0.511
		Sampling night × Location	0.977	0.953	1.00	0.893	0.876	0.909	1.03	1.01	1.05
		Site ID	0.823	0.729	0.927	0.584	0.508	0.671	0.979	0.863	1.12
		Site ID × Year	0.254	0.208	0.304	0.267	0.223	0.318	0.361	0.305	0.426
	Spline	Day of year	12.8	8.20	20.2	9.97	6.46	15.8	18.0	11.5	28.3
		Hours active	2.97	1.33	5.91	1.74	0.785	3.48	2.76	1.12	5.57

Nr. of traps: Number of traps
Sampl. prev. night: Sampling in previous night
Spat.-temp. cluster: Spatio-temporal cluster

Table S8 Detailed model results for the model analysing the effect of year, elevation, their interaction (and co-variates) on moth richness for different temperature niche categories (cold, intermediate, warm). Fixed effects of linear effects and splines and standard deviations of random factors and splines are given. For factors, sum-to-zero contrasts were used. Point estimates and 95% credible intervals (95%-CI) are given. If 95%-CI do not include zero, numbers are bold.

Para-meter	Type	Variable	Cold			Intermediate			Warm		
			Estimate	Lower 95%-CI	Upper 95%-CI	Estimate	Lower 95%-CI	Upper 95%-CI	Estimate	Lower 95%-CI	Upper 95%-CI
Fixed effect	Fixed	Year	**-0.149**	**-0.215**	**-0.0863**	-0.0185	-0.0759	0.0373	-0.00317	-0.0719	0.0661
		Elevation	**0.502**	**0.401**	**0.603**	**-0.247**	**-0.325**	**-0.173**	**-0.347**	**-0.454**	**-0.237**
		Precipitation	-0.000416	-0.0125	0.0112	**-0.0137**	**-0.0234**	**-0.00409**	-0.00177	-0.0121	0.00868
		Temperature	**0.446**	**0.422**	**0.470**	**0.550**	**0.531**	**0.568**	**0.456**	**0.434**	**0.478**
		Trap type (contr. sum 1)	**-0.490**	**-0.783**	**-0.204**	**-0.293**	**-0.527**	**-0.0624**	**-0.394**	**-0.718**	**-0.0639**
		Trap type (contr. sum 2)	0.126	-0.433	0.647	-0.142	-0.549	0.274	-0.0739	-0.673	0.519
		Lamp type (contr. sum 1)	-0.0405	-0.305	0.229	-0.0517	-0.275	0.178	0.0750	-0.187	0.344
		Lamp type (contr. sum 2)	0.156	-0.388	0.714	0.208	-0.289	0.684	0.0820	-0.471	0.587
		Lamp type (contr. sum 3)	-0.0455	-0.310	0.220	-0.0484	-0.267	0.180	0.139	-0.132	0.410
		Nr. of traps (linear)	0.491	-0.395	1.33	0.324	-0.370	1.05	0.307	-0.654	1.31
		Nr. of traps (quad-ratic)	-0.345	-1.02	0.350	-0.199	-0.758	0.360	-0.214	-0.953	0.505
		Nr. of traps (cubic)	0.0281	-0.293	0.370	0.0124	-0.259	0.289	-0.103	-0.466	0.257
		Sampl. prev. night (contr. sum 1)	-0.0288	-0.0949	0.0411	-0.0469	-0.0944	0.00279	0.0262	-0.0372	0.0879
		Year × Elevation	**0.109**	**0.0530**	**0.161**	**0.0876**	**0.0434**	**0.131**	-0.00701	-0.0606	0.0494
	Spline	Day of year	4.31	-3.58	12.	**-5.81**	**-9.29**	**-2.34**	**-6.24**	**-12.3**	**-0.404**
		Hours active	0.713	-5.28	6.34	2.28	-1.26	5.75	2.86	-1.93	7.79
SD	Random	Spat-temp. cluster	0.101	0.0337	0.154	0.0903	0.0206	0.142	0.0866	0.0215	0.137
		Sampling night × Location	0.625	0.606	0.644	0.455	0.432	0.474	0.558	0.541	0.574
		Site ID	0.521	0.457	0.588	0.392	0.342	0.447	0.601	0.532	0.682
		Site ID × Year	0.128	0.0980	0.161	0.151	0.124	0.179	0.116	0.0835	0.150
	Spline	Day of year	4.35	2.75	6.91	4.29	2.71	6.92	6.30	4.05	10.
		Hours active	3.39	1.57	6.10	1.28	0.513	2.68	2.41	1.09	4.44

Nr. of traps: Number of traps
Sampl. prev. night: Sampling in previous night
Spat.-temp. cluster: Spatio-temporal cluster

Table S9 Detailed model results for the model analysing the effect of year, elevation, their interaction (and co-variates) on moth biomass for different temperature niche categories (cold, intermediate, warm). Fixed effects of linear effects and splines and standard deviations of random factors and splines are given. For factors, sum-to-zero contrasts were used. Point estimates and 95% credible intervals (95%-CI) are given. If 95%-CI do not include zero, numbers are bold.

Para-meter	Type	Variable	Cold			Intermediate			Warm		
			**Estimate**	**Lower 95%-CI**	**Upper 95%-CI**	**Estimate**	**Lower 95%-CI**	**Upper 95%-CI**	**Estimate**	**Lower 95%-CI**	**Upper 95%-CI**
Fixed effect	Fixed	Year	**-0.197**	**-0.282**	**-0.111**	-0.0375	-0.116	0.0447	0.0288	-0.0730	0.134
		Elevation	**0.996**	**0.867**	**1.12**	**0.237**	**0.126**	**0.353**	0.0925	-0.0736	0.261
		Precipitation	**0.0205**	**0.00518**	**0.0358**	0.00235	-0.00959	0.0146	**0.0228**	**0.00852**	**0.0372**
		Temperature	**0.403**	**0.371**	**0.434**	**0.466**	**0.441**	**0.490**	**0.369**	**0.338**	**0.399**
		Trap type (contr. sum 1)	-0.328	-0.698	0.0525	-0.261	-0.611	0.0771	-0.407	-0.874	0.0545
		Trap type (contr. sum 2)	-0.310	-1.01	0.380	**-0.637**	**-1.26**	**-0.0161**	-0.516	-1.36	0.351
		Lamp type (contr. sum 1)	-0.0105	-0.354	0.340	-0.185	-0.507	0.142	-0.0761	-0.492	0.339
		Lamp type (contr. sum 2)	-0.184	-0.950	0.560	0.0872	-0.614	0.747	0.257	-0.495	1.02
		Lamp type (contr. sum 3)	-0.191	-0.536	0.157	-0.329	-0.650	0.00402	-0.136	-0.545	0.285
		Nr. of traps (linear)	1.08	-0.0474	2.22	0.394	-0.572	1.37	-0.176	-1.50	1.15
		Nr. of traps (quad-ratic)	-0.571	-1.45	0.309	-0.446	-1.20	0.298	-0.284	-1.34	0.777
		Nr. of traps (cubic)	-0.0141	-0.457	0.408	-0.288	-0.639	0.0839	-0.444	-0.928	0.0648
		Sampl. prev. night (contr. sum 1)	-0.0332	-0.122	0.0537	-0.0451	-0.111	0.0178	-0.0846	-0.172	0.00590
		Year × Elevation	**0.165**	**0.0969**	**0.237**	**0.0720**	**0.00634**	**0.136**	-0.0897	-0.177	0.00304
	Spline	Day of year	-0.0683	-9.02	8.82	**-16.9**	**-21.6**	**-12.2**	**-18.6**	**-26.2**	**-11.2**
		Hours active	4.24	-2.41	10.9	**5.58**	**0.753**	**10.0**	2.24	-3.43	7.95
SD	Random	Spat-temp. cluster	0.106	0.0115	0.194	0.282	0.206	0.353	0.300	0.231	0.371
		Sampling night × Location	0.594	0.563	0.627	0.701	0.680	0.724	0.769	0.746	0.790
		Site ID	0.664	0.587	0.751	0.526	0.458	0.603	0.824	0.727	0.938
		Site ID × Year	0.220	0.176	0.260	0.244	0.199	0.291	0.195	0.151	0.245
	Spline	Day of year	7.36	4.74	11.9	9.07	5.83	14.5	14.1	9.17	22.0
		Hours active	4.13	1.93	7.62	1.71	0.741	3.43	2.80	1.04	6.01

Nr. of traps: Number of traps
Sampl. prev. night: Sampling in previous night
Spat.-temp. cluster: Spatio-temporal cluster

Table S10 Detailed model results for the model analysing the effect of year, elevation, their interaction (and co-variates) on moth abundance for different food specialisation categories (monophagous, oligophagous, polyphagous). Fixed effects of linear effects and splines and standard deviations of random factors and splines are given. For factors, sum-to-zero contrasts were used. Point estimates and 95% credible intervals (95%-CI) are given. If 95%-CI do not include zero, numbers are bold.

Para-meter	Type	Variable	Monophagous			Oligophagous			Polyphagous		
			Estimate	Lower 95%-CI	Upper 95%-CI	Estimate	Lower 95%-CI	Upper 95%-CI	Estimate	Lower 95%-CI	Upper 95%-CI
Fixed effect	Fixed	Year	**-0.178**	**-0.295**	**-0.0644**	-0.0880	-0.195	0.0200	-0.0317	-0.116	0.0522
		Elevation	**0.550**	**0.351**	**0.737**	**0.322**	**0.168**	**0.471**	**0.398**	**0.275**	**0.528**
		Precipitation	0.00733	-0.0115	0.0262	0.00723	-0.00798	0.0227	0.0116	-0.0000961	0.0235
		Temperature	**1.23**	**1.19**	**1.27**	**0.836**	**0.805**	**0.868**	**0.696**	**0.672**	**0.721**
		Trap type (contr. sum 1)	**-1.26**	**-1.82**	**-0.704**	**-0.660**	**-1.12**	**-0.204**	**-0.461**	**-0.827**	**-0.0852**
		Trap type (contr. sum 2)	-0.0976	-1.04	0.933	-0.593	-1.41	0.225	**-0.724**	**-1.40**	**-0.0387**
		Lamp type (contr. sum 1)	0.0167	-0.432	0.488	-0.201	-0.588	0.200	-0.148	-0.481	0.185
		Lamp type (contr. sum 2)	0.149	-0.743	1.01	0.389	-0.375	1.18	0.158	-0.544	0.844
		Lamp type (contr. sum 3)	-0.0674	-0.515	0.404	-0.341	-0.730	0.0577	-0.237	-0.569	0.0950
		Nr. of traps (linear)	0.775	-0.682	2.26	0.361	-0.922	1.66	0.401	-0.663	1.48
		Nr. of traps (quad-ratic)	-0.604	-1.77	0.580	-0.472	-1.53	0.549	-0.437	-1.25	0.356
		Nr. of traps (cubic)	0.0263	-0.570	0.612	-0.342	-0.833	0.160	-0.227	-0.622	0.177
		Sampl. prev. night (contr. sum 1)	-0.104	-0.219	0.0106	**-0.157**	**-0.259**	**-0.0497**	**-0.0940**	**-0.158**	**-0.0280**
		Year × Elevation	**0.102**	**0.00561**	**0.201**	**0.116**	**0.0341**	**0.198**	0.0377	-0.0328	0.107
	Spline	Day of year	4.05	-5.39	13.5	**-12.6**	**-20.2**	**-4.46**	**-25.8**	**-30.3**	**-21.1**
		Hours active	1.93	-4.66	8.18	3.98	-3.02	10.2	**6.66**	**1.86**	**11.5**
SD	Random	Spat-temp. cluster	0.181	0.0461	0.277	0.143	0.0121	0.253	0.286	0.218	0.353
		Sampling night × Location	1.07	1.04	1.09	0.907	0.886	0.929	0.815	0.798	0.831
		Site ID	0.962	0.853	1.09	0.767	0.678	0.869	0.598	0.523	0.680
		Site ID × Year	0.296	0.242	0.355	0.319	0.269	0.372	0.229	0.192	0.272
	Spline	Day of year	15.	9.56	23.7	6.47	4.13	10.5	13.3	8.74	21.2
		Hours active	2.92	1.45	5.19	3.50	1.42	6.91	1.89	0.943	3.53

Nr. of traps: Number of traps
Sampl. prev. night: Sampling in previous night
Spat.-temp. cluster: Spatio-temporal cluster

Table S11 Detailed model results for the model analysing the effect of year, elevation, their interaction (and co-variates) on moth richness for different food specialisation categories (monophagous, oligophagous, polyphagous). Fixed effects of linear effects and splines and standard deviations of random factors and splines are given. For factors, sum-to-zero contrasts were used. Point estimates and 95% credible intervals (95%-CI) are given. If 95%-CI do not include zero, numbers are bold.

Para-meter	Type	Variable	**Monophagous**			**Oligophagous**			**Polyphagous**		
			**Estimate**	**Lower 95%-CI**	**Upper 95%-CI**	**Estimate**	**Lower 95%-CI**	**Upper 95%-CI**	**Estimate**	**Lower 95%-CI**	**Upper 95%-CI**
Fixed effect	Fixed	Year	**-0.0687**	**-0.134**	**-0.00765**	**-0.0741**	**-0.137**	**-0.0145**	-0.00221	-0.0565	0.0521
		Elevation	**0.133**	**0.0335**	**0.230**	**0.105**	**0.0107**	**0.198**	-0.0715	-0.149	0.00472
		Precipitation	**0.0135**	**0.000885**	**0.0270**	-0.00503	-0.0165	0.00616	-0.000303	-0.00958	0.00907
		Temperature	**0.507**	**0.479**	**0.534**	**0.476**	**0.453**	**0.499**	**0.534**	**0.515**	**0.554**
		Trap type (contr. sum 1)	**-0.571**	**-0.852**	**-0.285**	**-0.370**	**-0.623**	**-0.121**	**-0.320**	**-0.537**	**-0.106**
		Trap type (contr. sum 2)	0.152	-0.339	0.648	-0.133	-0.596	0.318	-0.154	-0.558	0.243
		Lamp type (contr. sum 1)	-0.0158	-0.252	0.228	-0.0551	-0.276	0.175	-0.0420	-0.252	0.171
		Lamp type (contr. sum 2)	0.215	-0.296	0.708	-0.0410	-0.547	0.476	0.0820	-0.385	0.542
		Lamp type (contr. sum 3)	0.0222	-0.216	0.276	-0.0826	-0.305	0.155	0.00416	-0.209	0.220
		Nr. of traps (linear)	0.105	-0.697	0.918	0.547	-0.229	1.34	0.360	-0.315	1.04
		Nr. of traps (quad-ratic)	-0.417	-1.06	0.232	-0.341	-0.951	0.264	-0.283	-0.808	0.239
		Nr. of traps (cubic)	-0.194	-0.511	0.107	-0.0106	-0.307	0.296	-0.0166	-0.273	0.245
		Sampl. prev. night (contr. sum 1)	0.0498	-0.0309	0.135	-0.0656	-0.141	0.0106	**-0.0696**	**-0.117**	**-0.0239**
		Year × Elevation	0.0347	-0.0183	0.0881	**0.0797**	**0.0316**	**0.132**	**0.0623**	**0.0190**	**0.106**
	Spline	Day of year	-0.151	-7.03	7.26	-4.73	-11.5	1.87	**-3.75**	**-6.86**	**-0.635**
		Hours active	-0.277	-6.75	5.69	0.645	-4.73	5.52	**2.88**	**0.354**	**5.85**
SD	Random	Spat-temp. cluster	0.0432	0.00163	0.102	0.0627	0.00536	0.116	0.126	0.0828	0.167
		Sampling night × Location	0.568	0.550	0.586	0.572	0.555	0.591	0.404	0.384	0.425
		Site ID	0.498	0.437	0.564	0.463	0.409	0.525	0.371	0.323	0.425
		Site ID × Year	0.141	0.110	0.172	0.150	0.120	0.179	0.141	0.117	0.168
	Spline	Day of year	3.62	2.10	6.34	3.58	2.18	6.16	3.98	2.50	6.61
		Hours active	3.64	1.80	6.61	2.15	0.935	4.22	0.784	0.266	1.82

Nr. of traps: Number of traps
Sampl. prev. night: Sampling in previous night
Spat.-temp. cluster: Spatio-temporal cluster

Table S12 Detailed model results for the model analysing the effect of year, elevation, their interaction (and co-variates) on moth biomass for different food specialisation categories (monophagous, oligophagous, polyphagous). Fixed effects of linear effects and splines and standard deviations of random factors and splines are given. For factors, sum-to-zero contrasts were used. Point estimates and 95% credible intervals (95%-CI) are given. If 95%-CI do not include zero, numbers are bold.

Para-meter	Type	Variable	Monophagous			Oligophagous			Polyphagous		
			**Estimate**	**Lower 95%-CI**	**Upper 95%-CI**	**Estimate**	**Lower 95%-CI**	**Upper 95%-CI**	**Estimate**	**Lower 95%-CI**	**Upper 95%-CI**
Fixed effect	Fixed	Year	-0.0497	-0.145	0.0420	-0.0598	-0.151	0.0296	-0.0227	-0.103	0.0626
		Elevation	**0.339**	**0.208**	**0.476**	**0.466**	**0.331**	**0.600**	**0.431**	**0.318**	**0.550**
		Precipitation	**0.0197**	**0.00163**	**0.0383**	**0.0194**	**0.00267**	**0.0362**	**0.0157**	**0.00413**	**0.0272**
		Temperature	**0.523**	**0.483**	**0.563**	**0.412**	**0.377**	**0.447**	**0.475**	**0.451**	**0.498**
		Trap type (contr. sum 1)	**-0.522**	**-0.927**	**-0.113**	-0.184	-0.570	0.196	-0.350	-0.711	0.0168
		Trap type (contr. sum 2)	0.00279	-0.728	0.725	-0.672	-1.36	0.0208	**-0.717**	**-1.39**	**-0.0945**
		Lamp type (contr. sum 1)	-0.0723	-0.432	0.299	-0.241	-0.587	0.121	-0.136	-0.449	0.168
		Lamp type (contr. sum 2)	0.148	-0.587	0.860	0.178	-0.612	0.944	-0.0702	-0.744	0.629
		Lamp type (contr. sum 3)	-0.192	-0.547	0.180	**-0.383**	**-0.720**	**-0.0159**	-0.251	-0.564	0.0586
		Nr. of traps (linear)	0.243	-0.928	1.41	0.336	-0.853	1.49	0.391	-0.604	1.35
		Nr. of traps (quad-ratic)	-0.527	-1.46	0.416	-0.459	-1.38	0.451	-0.581	-1.30	0.153
		Nr. of traps (cubic)	-0.223	-0.668	0.234	**-0.489**	**-0.923**	**-0.0486**	-0.263	-0.624	0.101
		Sampl. prev. night (contr. sum 1)	0.0895	-0.0242	0.204	-0.0752	-0.188	0.0382	**-0.124**	**-0.183**	**-0.0608**
		Year × Elevation	0.0555	-0.0179	0.132	**0.0775**	**0.00597**	**0.149**	0.0376	-0.0299	0.107
	Spline	Day of year	-0.271	-9.00	8.55	-3.14	-11.1	4.17	**-19.9**	**-23.9**	**-16.1**
		Hours active	2.96	-2.63	8.66	0.351	-7.45	8.02	**5.43**	**0.945**	**9.93**
SD	Random	Spat-temp. cluster	0.193	0.124	0.259	0.155	0.0482	0.230	0.274	0.202	0.343
		Sampling night × Location	0.614	0.575	0.653	0.769	0.741	0.799	0.648	0.626	0.671
		Site ID	0.680	0.592	0.778	0.659	0.580	0.746	0.555	0.485	0.633
		Site ID × Year	0.195	0.150	0.244	0.234	0.186	0.284	0.226	0.188	0.272
	Spline	Day of year	7.51	4.67	12.1	4.47	2.73	7.49	10.9	6.99	18.3
		Hours active	2.20	1.11	4.13	5.95	2.95	10.4	1.79	0.791	3.61

Nr. of traps: Number of traps
Sampl. prev. night: Sampling in previous night
Spat.-temp. cluster: Spatio-temporal cluster

Table S13 Detailed model results for the model analysing the effect of year, elevation, their interaction (and covariates) on moth abundance for different overwintering stages (egg, larva, pupa, adult). Fixed effects of linear effects and splines and standard deviations of random factors and splines are given. For factors, sum-to-zero contrasts were used. Point estimates and 95% credible intervals (95%-CI) are given. If 95%-CI do not include zero, numbers are bold.

Para-meter	Type	Variable	Egg			Larva			Pupa			Adult		
			Estimate	Lower 95%-CI	Upper 95%-CI	Estimate	Lower 95%-CI	Upper 95%-CI	Estimate	Lower 95%-CI	Upper 95%-CI	Estimate	Lower 95%-CI	Upper 95%-CI
Fixed effect	Fixed	Year	-0.0597	-0.172	0.0562	-0.0296	-0.127	0.0708	-0.0536	-0.152	0.0469	-0.117	-0.242	0.0136
		Elevation	**0.312**	**0.143**	**0.476**	**0.305**	**0.167**	**0.445**	**0.147**	**0.00642**	**0.286**	**0.945**	**0.770**	**1.13**
		Precipitation	0.00822	-0.0110	0.0285	**0.0241**	**0.0103**	**0.0382**	0.00289	-0.0127	0.0184	-0.0208	-0.0510	0.00630
		Temperature	**0.844**	**0.801**	**0.888**	**0.585**	**0.555**	**0.614**	**0.992**	**0.961**	**1.02**	**0.964**	**0.906**	**1.02**
		Trap type (contr. sum 1)	**-0.680**	**-1.16**	**-0.233**	**-0.581**	**-0.991**	**-0.159**	**-0.653**	**-1.09**	**-0.224**	**-0.784**	**-1.34**	**-0.275**
		Trap type (contr. sum 2)	-0.310	-1.15	0.490	-0.699	-1.47	0.0475	-0.448	-1.23	0.311	-0.730	-1.65	0.258
		Lamp type (contr. sum 1)	-0.231	-0.682	0.193	-0.0812	-0.447	0.297	-0.0968	-0.477	0.270	-0.112	-0.619	0.409
		Lamp type (contr. sum 2)	**1.10**	**0.139**	**2.09**	-0.165	-0.948	0.583	-0.149	-0.890	0.657	0.536	-0.605	1.70
		Lamp type (contr. sum 3)	-0.167	-0.609	0.257	-0.126	-0.502	0.250	-0.194	-0.597	0.184	-0.113	-0.635	0.403
		Nr. of traps (linear)	0.510	-0.878	1.99	0.445	-0.725	1.59	0.399	-0.804	1.57	0.508	-1.07	2.18
		Nr. of traps (quad-ratic)	0.574	-0.547	1.66	**-0.924**	**-1.85**	**-0.00978**	-0.799	-1.70	0.142	-0.00645	-1.20	1.19
		Nr. of traps (cubic)	0.135	-0.397	0.659	-0.439	-0.882	0.00949	-0.155	-0.621	0.302	-0.282	-0.867	0.323
		Sampl. prev. night (contr. sum 1)	0.129	-0.00669	0.255	**-0.207**	**-0.303**	**-0.111**	-0.0107	-0.0923	0.0700	-0.0318	-0.149	0.0813
		Year × Elevation	0.0871	-0.00507	0.182	-0.00555	-0.0852	0.0724	**0.137**	**0.0588**	**0.218**	-0.0357	-0.139	0.0638
	Spline	Day of year	**-10.5**	**-19.2**	**-1.50**	**-8.57**	**-16.4**	**-0.394**	**-12.4**	**-20.3**	**-4.45**	2.67	-3.58	8.85
		Hours active	5.04	-2.39	11.8	**5.48**	**0.639**	**10.5**	4.91	-0.529	9.77	5.35	-1.40	11.8
SD	Random	Spat-temp. cluster	0.247	0.136	0.342	0.406	0.324	0.489	0.257	0.178	0.333	0.288	0.184	0.390
		Sampling night × Location	1.09	1.05	1.12	0.893	0.874	0.913	0.965	0.946	0.983	1.18	1.14	1.22
		Site ID	0.809	0.712	0.917	0.646	0.558	0.739	0.698	0.608	0.800	0.877	0.764	1.01
		Site ID × Year	0.264	0.207	0.326	0.256	0.212	0.307	0.273	0.230	0.319	0.310	0.247	0.375
	Spline	Day of year	16.2	10.3	26.3	11.6	7.52	18.1	17.2	11.4	26.3	15.3	9.75	26.6
		Hours active	3.66	1.62	7.24	1.81	0.885	3.51	1.92	0.757	4.03	3.61	1.41	7.06

Nr. of traps: Number of traps
Sampl. prev. night: Sampling in previous night
Spat.-temp. cluster: Spatio-temporal cluster

Table S14 Detailed model results for the model analysing the effect of year, elevation, their interaction (and covariates) on moth richness for different overwintering stages (egg, larva, pupa, adult). Fixed effects of linear effects and splines and standard deviations of random factors and splines are given. For factors, sum-to-zero contrasts were used. Point estimates and 95% credible intervals (95%-CI) are given. If 95%-CI do not include zero, numbers are bold.

			**Egg**			**Larva**			**Pupa**			**Adult**		
**Para-meter**	**Type**	**Variable**	**Estimate**	**Lower 95%-CI**	**Upper 95%-CI**	**Estimate**	**Lower 95%-CI**	**Upper 95%-CI**	**Estimate**	**Lower 95%-CI**	**Upper 95%-CI**	**Estimate**	**Lower 95%-CI**	**Upper 95%-CI**
Fixed effect	Fixed	Year	**-0.0666**	**-0.121**	**-0.00992**	-0.00723	-0.0650	0.0493	-0.00265	-0.0664	0.0634	**-0.0425**	**-0.0814**	**-0.00437**
		Elevation	0.0703	-0.00698	0.148	-0.0134	-0.0924	0.0689	**-0.128**	**-0.215**	**-0.0400**	**0.187**	**0.133**	**0.240**
		Precipitation	-0.000467	-0.0122	0.0113	0.00551	-0.00458	0.0158	-0.00109	-0.0135	0.0109	0.00366	-0.00957	0.0172
		Temperature	**0.279**	**0.251**	**0.308**	**0.472**	**0.449**	**0.493**	**0.653**	**0.628**	**0.677**	**0.176**	**0.150**	**0.202**
		Trap type (contr. sum 1)	**-0.382**	**-0.601**	**-0.154**	**-0.299**	**-0.540**	**-0.0591**	**-0.332**	**-0.607**	**-0.0675**	-0.144	-0.297	0.0120
		Trap type (contr. sum 2)	0.116	-0.273	0.515	-0.0742	-0.502	0.342	-0.271	-0.761	0.214	-0.120	-0.386	0.144
		Lamp type (contr. sum 1)	-0.144	-0.366	0.0683	-0.0288	-0.255	0.185	-0.100	-0.349	0.170	-0.109	-0.283	0.0705
		Lamp type (contr. sum 2)	**0.792**	**0.281**	**1.29**	-0.0283	-0.560	0.482	0.132	-0.426	0.661	0.198	-0.263	0.632
		Lamp type (contr. sum 3)	-0.0891	-0.319	0.125	0.0177	-0.217	0.239	-0.0573	-0.314	0.207	-0.0758	-0.252	0.101
		Nr. of traps (linear)	0.181	-0.519	0.902	0.159	-0.577	0.909	0.201	-0.597	0.986	0.205	-0.318	0.730
		Nr. of traps (quad-ratic)	0.359	-0.190	0.916	**-0.562**	**-1.11**	**-0.0165**	-0.280	-0.896	0.348	0.112	-0.296	0.509
		Nr. of traps (cubic)	0.0129	-0.255	0.285	-0.141	-0.426	0.144	-0.0471	-0.346	0.251	-0.00792	-0.207	0.194
		Sampl. prev. night (contr. sum 1)	-0.0155	-0.0920	0.0626	-0.0178	-0.0910	0.0575	-0.0446	-0.103	0.0141	-0.0435	-0.0938	0.00507
		Year × Elevation	0.0334	-0.0126	0.0793	0.0447	-0.000831	0.0904	**0.0950**	**0.0449**	**0.147**	-0.0000218	-0.0319	0.0311
	Spline	Day of year	-3.25	-8.70	2.39	**-6.80**	**-13.3**	**-0.497**	**-10.1**	**-17.7**	**-2.60**	1.14	-1.56	3.86
		Hours active	2.55	-1.51	6.11	1.19	-2.65	4.83	-1.04	-6.61	4.43	2.68	-0.351	6.42
SD	Random	Spat-temp. cluster	0.0882	0.0194	0.140	0.133	0.0754	0.185	0.155	0.103	0.204	0.0744	0.0169	0.117
		Sampling night × Location	0.563	0.547	0.577	0.454	0.432	0.476	0.540	0.513	0.566	0.383	0.369	0.397
		Site ID	0.372	0.323	0.425	0.386	0.333	0.445	0.433	0.378	0.494	0.227	0.193	0.266
		Site ID × Year	0.110	0.0733	0.146	0.150	0.122	0.181	0.149	0.119	0.183	0.100	0.0661	0.136
	Spline	Day of year	4.68	2.80	7.74	5.96	3.82	9.57	9.30	5.98	15.2	3.02	1.86	5.03
		Hours active	1.24	0.371	3.11	1.18	0.414	2.83	2.61	1.37	4.74	1.22	0.187	2.74

Nr. of traps: Number of traps
Sampl. prev. night: Sampling in previous night
Spat.-temp. cluster: Spatio-temporal cluster

Table S15 Detailed model results for the model analysing the effect of year, elevation, their interaction (and covariates) on moth biomass for different overwintering stages (egg, larva, pupa, adult). Fixed effects of linear effects and splines and standard deviations of random factors and splines are given. For factors, sum-to-zero contrasts were used. Point estimates and 95% credible intervals (95%-CI) are given. If 95%-CI do not include zero, numbers are bold.

Para-meter	Type	Variable	Egg			Larva			Pupa			Adult		
			Estimate	Lower 95%-CI	Upper 95%-CI	Estimate	Lower 95%-CI	Upper 95%-CI	Estimate	Lower 95%-CI	Upper 95%-CI	Esti-mate	Lower 95%-CI	Upper 95%-CI
Fixed effect	Fixed	Year	0.0345	-0.0563	0.126	-0.0540	-0.146	0.0343	0.0304	-0.0575	0.117	**-0.104**	**-0.174**	**-0.0326**
		Elevation	**0.173**	**0.0384**	**0.309**	**0.391**	**0.270**	**0.518**	**0.439**	**0.318**	**0.557**	**0.438**	**0.335**	**0.536**
		Precipitation	**0.0182**	**0.00123**	**0.0348**	**0.0270**	**0.0138**	**0.0403**	0.0124	-0.00453	0.0286	**0.0270**	**0.00309**	**0.0510**
		Temperature	**0.338**	**0.300**	**0.376**	**0.343**	**0.316**	**0.370**	**0.635**	**0.601**	**0.667**	**0.315**	**0.268**	**0.361**
		Trap type (contr. sum 1)	-0.359	-0.755	0.0351	-0.310	-0.688	0.0540	-0.139	-0.512	0.227	-0.0550	-0.331	0.231
		Trap type (contr. sum 2)	-0.323	-1.03	0.374	**-0.721**	**-1.39**	**-0.0453**	**-0.858**	**-1.52**	**-0.218**	**-0.532**	**-1.02**	**-0.0339**
		Lamp type (contr. sum 1)	-0.262	-0.603	0.103	-0.0617	-0.396	0.264	-0.136	-0.480	0.210	-0.290	-0.608	0.0445
		Lamp type (contr. sum 2)	**0.807**	**0.0269**	**1.57**	-0.438	-1.16	0.272	0.148	-0.586	0.895	0.445	-0.373	1.24
		Lamp type (contr. sum 3)	-0.188	-0.540	0.173	-0.180	-0.510	0.151	-0.223	-0.566	0.130	-0.239	-0.560	0.0922
		Nr. of traps (linear)	0.541	-0.563	1.67	0.414	-0.659	1.51	0.0246	-1.08	1.15	0.240	-0.738	1.21
		Nr. of traps (quad-ratic)	0.549	-0.327	1.47	**-0.958**	**-1.76**	**-0.132**	-0.606	-1.43	0.208	0.226	-0.525	0.971
		Nr. of traps (cubic)	0.111	-0.328	0.541	-0.369	-0.769	0.0368	**-0.429**	**-0.833**	**-0.0158**	-0.149	-0.520	0.215
		Sampl. prev. night (contr. sum 1)	0.0586	-0.0455	0.165	**-0.127**	**-0.219**	**-0.0320**	**-0.102**	**-0.185**	**-0.0181**	**-0.0890**	**-0.174**	**-0.00364**
		Year × Elevation	0.0598	-0.0182	0.135	0.0227	-0.0466	0.0920	**0.0883**	**0.0179**	**0.158**	-0.0227	-0.0815	0.0358
	Spline	Day of year	-3.83	-10.9	3.44	**-11.5**	**-18.7**	**-4.19**	-4.88	-13.7	3.40	3.33	-1.35	8.00
		Hours active	4.81	-2.06	10.5	2.88	-2.97	8.86	1.72	-5.91	7.95	4.74	-1.43	11.4
SD	Random	Spat-temp. cluster	0.156	0.0398	0.243	0.353	0.284	0.428	0.246	0.189	0.308	0.189	0.115	0.254
		Sampling night × Location	0.661	0.631	0.692	0.675	0.650	0.697	0.771	0.741	0.799	0.713	0.685	0.740
		Site ID	0.646	0.568	0.737	0.582	0.505	0.669	0.578	0.504	0.661	0.428	0.368	0.497
		Site ID × Year	0.234	0.181	0.291	0.226	0.185	0.272	0.193	0.154	0.235	0.178	0.122	0.235
	Spline	Day of year	9.59	5.99	15.4	8.09	5.31	13.	10.3	6.64	16.2	6.10	3.98	9.70
		Hours active	2.77	1.24	5.53	2.64	1.18	5.20	2.78	1.09	5.76	3.89	1.98	7.07

Nr. of traps: Number of traps
Sampl. prev. night: Sampling in previous night
Spat.-temp. cluster: Spatio-temporal cluster

Table S16 Detailed model results for the model analysing the effect of year, elevation, their interaction (and co-variates) on moth abundance, richness, and biomass for the dataset only including samples from fixed traps. Fixed effects of linear effects and splines and standard deviations of random factors and splines are given. For factors, sum-to-zero contrasts were used. Point estimates and 95% credible intervals (95%-CI) are given. If 95%-CI do not include zero, numbers are bold.

			Abundance			Richness			Biomass		
**Para-meter**	**Type**	**Variable**	**Estimate**	**Lower 95%-CI**	**Upper 95%-CI**	**Estimate**	**Lower 95%-CI**	**Upper 95%-CI**	**Estimate**	**Lower 95%-CI**	**Upper 95%-CI**
Fixed effect	Fixed	Year	0.0243	-0.162	0.208	-0.0122	-0.131	0.101	0.00382	-0.162	0.169
		Elevation	**0.446**	**0.244**	**0.663**	-0.00944	-0.152	0.132	**0.509**	**0.317**	**0.702**
		Precipitation	**0.0149**	**0.00211**	**0.0275**	-0.0000774	-0.0104	0.0101	**0.0193**	**0.00737**	**0.0318**
		Temperature	**0.744**	**0.719**	**0.769**	**0.605**	**0.583**	**0.626**	**0.529**	**0.504**	**0.554**
		Trap type (contr. sum 1)	0.167	-0.414	0.738	-0.0734	-0.471	0.334	0.258	-0.318	0.834
		Lamp type (contr. sum 1)	-0.106	-0.488	0.269	-0.0502	-0.328	0.204	-0.197	-0.566	0.170
		Lamp type (contr. sum 2)	-0.168	-0.550	0.196	0.0174	-0.259	0.271	-0.278	-0.641	0.0864
		Sampl. prev. night (contr. sum 1)	**-0.124**	**-0.201**	**-0.0461**	**-0.138**	**-0.193**	**-0.0813**	**-0.201**	**-0.271**	**-0.126**
		Year × Elevation	0.0490	-0.103	0.202	**0.134**	**0.0352**	**0.242**	0.0615	-0.0830	0.213
	Spline	Day of year	**-23.7**	**-28.6**	**-19.0**	-2.81	-6.57	1.05	**-17.2**	**-21.7**	**-12.7**
SD	Random	Spat-temp. cluster	0.300	0.120	0.430	0.0993	0.00959	0.178	0.297	0.0702	0.438
		Sampling night × Location	0.636	0.611	0.663	0.343	0.321	0.364	0.555	0.534	0.577
		Site ID	0.663	0.512	0.841	0.486	0.380	0.617	0.611	0.471	0.779
		Site ID × Year	0.330	0.249	0.433	0.193	0.149	0.240	0.324	0.237	0.441
	Spline	Day of year	12.7	8.31	20.4	4.38	2.79	7.32	10.7	6.95	16.8

Sampl. prev. night: Sampling in previous night
Spat.-temp. cluster: Spatio-temporal cluster

Table S17 Detailed model results for the model analysing the effect of year, elevation, their interaction (and co-variates) on moth abundance, richness, and biomass for the dataset only including samples from manual traps. Fixed effects of linear effects and splines and standard deviations of random factors and splines are given. For factors, sum-to-zero contrasts were used. Point estimates and 95% credible intervals (95%-CI) are given. If 95%-CI do not include zero, numbers are bold.

Para-meter	Type	Variable	Abundance			Richness			Biomass		
			Estimate	Lower 95%-CI	Upper 95%-CI	Estimate	Lower 95%-CI	Upper 95%-CI	Estimate	Lower 95%-CI	Upper 95%-CI
Fixed effect	Fixed	Year	**-0.116**	**-0.215**	**-0.0128**	-0.0527	-0.121	0.0165	-0.0707	-0.178	0.0362
		Elevation	**0.405**	**0.278**	**0.530**	-0.0283	-0.104	0.0496	**0.459**	**0.334**	**0.581**
		Precipitation	**0.0551**	**0.0248**	**0.0836**	**0.0335**	**0.0122**	**0.0555**	**0.0613**	**0.0299**	**0.0919**
		Temperature	**0.463**	**0.400**	**0.527**	**0.307**	**0.263**	**0.349**	**0.418**	**0.351**	**0.485**
		Lamp type (contr. sum 1)	-0.0367	-0.281	0.218	-0.00869	-0.185	0.161	0.0493	-0.206	0.309
		Lamp type (contr. sum 2)	0.372	-0.105	0.862	0.193	-0.127	0.528	0.244	-0.250	0.732
		Nr. of traps (linear)	0.222	-0.694	1.14	0.171	-0.421	0.763	0.154	-0.738	1.06
		Nr. of traps (quad-ratic)	-0.347	-1.02	0.349	-0.178	-0.635	0.274	-0.435	-1.12	0.281
		Nr. of traps (cubic)	-0.259	-0.599	0.0750	-0.0742	-0.301	0.155	-0.289	-0.643	0.0528
		Sampl. prev. night (contr. sum 1)	-0.00590	-0.0953	0.0879	0.0172	-0.0471	0.0834	0.0485	-0.0538	0.145
		Year × Elevation	-0.00583	-0.118	0.104	0.00798	-0.0654	0.0775	0.0246	-0.0887	0.139
	Spline	Day of year	-2.47	-9.11	4.14	1.81	-2.57	6.33	-1.17	-7.89	5.34
		Hours active	**5.98**	**1.29**	**10.4**	**3.70**	**0.703**	**7.00**	**5.74**	**1.17**	**10.1**
SD	Random	Spat-temp. cluster	0.202	0.130	0.277	0.102	0.0469	0.154	0.196	0.130	0.266
		Sampling night × Location	0.607	0.580	0.635	0.326	0.296	0.355	0.613	0.583	0.642
		Site ID	0.556	0.476	0.643	0.339	0.290	0.399	0.542	0.464	0.632
		Site ID × Year	0.106	0.0477	0.166	0.0543	0.00483	0.103	0.0873	0.0149	0.150
	Spline	Day of year	9.19	5.83	14.9	2.65	1.62	4.52	8.91	5.71	14.0
		Hours active	2.13	1.08	4.00	1.17	0.525	2.42	1.88	0.866	3.64

Nr. of traps: Number of traps
Sampl. prev. night: Sampling in previous night
Spat.-temp. cluster: Spatio-temporal cluster

Table S18 Detailed model results for the model analysing the effect of year, elevation, their interaction (and co-variates) on moth abundance, richness, and biomass for the dataset only including full sampling duration information (manual traps). Fixed effects of linear effects and splines and standard deviations of random factors and splines are given. For factors, sum-to-zero contrasts were used. Point estimates and 95% credible intervals (95%-CI) are given. If 95%-CI do not include zero, numbers are bold.

Parameter	Type	Variable	Abundance			Richness			Biomass		
			**Estimate**	**Lower 95%-CI**	**Upper 95%-CI**	**Estimate**	**Lower 95%-CI**	**Upper 95%-CI**	**Estimate**	**Lower 95%-CI**	**Upper 95%-CI**
Fixed effect	Fixed	Year	0.00561	-0.101	0.109	-0.00169	-0.0691	0.0646	0.0172	-0.0816	0.118
		Elevation	**0.394**	**0.264**	**0.527**	-0.0203	-0.102	0.0650	**0.448**	**0.319**	**0.585**
		Precipitation	**0.0147**	**0.00281**	**0.0270**	0.0000371	-0.00966	0.00973	**0.0208**	**0.00862**	**0.0330**
		Temperature	**0.733**	**0.708**	**0.756**	**0.585**	**0.566**	**0.604**	**0.526**	**0.502**	**0.550**
		Trap type (contr. sum 1)	**-0.397**	**-0.784**	**-0.0144**	**-0.332**	**-0.574**	**-0.0720**	-0.273	-0.646	0.105
		Trap type (contr. sum 2)	**-0.730**	**-1.44**	**-0.0249**	-0.169	-0.600	0.268	**-0.800**	**-1.46**	**-0.135**
		Lamp type (contr. sum 1)	-0.0699	-2.18	1.99	-0.185	-2.19	1.85	-0.300	-2.30	1.65
		Lamp type (contr. sum 2)	-0.187	-6.26	6.02	0.426	-5.75	6.42	0.271	-5.45	6.23
		Lamp type (contr. sum 3)	-0.154	-2.25	1.92	-0.129	-2.13	1.91	-0.400	-2.40	1.55
		Nr. of traps (linear)	0.825	-4.94	6.26	0.0863	-5.33	5.70	0.143	-5.20	5.40
		Nr. of traps (quadratic)	-0.751	-4.87	3.52	-0.108	-4.27	3.95	-0.451	-4.36	3.57
		Nr. of traps (cubic)	-0.153	-2.10	1.65	-0.173	-2.00	1.70	-0.439	-2.24	1.35
		Sampl. prev. night (contr. sum 1)	**-0.118**	**-0.186**	**-0.0503**	**-0.117**	**-0.169**	**-0.0664**	**-0.171**	**-0.234**	**-0.108**
		Year × Elevation	0.0607	-0.0184	0.137	**0.0767**	**0.0273**	**0.126**	0.0480	-0.0235	0.119
	Spline	Day of year	**-24.2**	**-28.7**	**-19.6**	-2.49	-5.79	0.503	**-17.5**	**-21.7**	**-13.4**
		Hours active	**6.57**	**1.63**	**11.3**	2.55	-0.377	5.85	**5.65**	**1.06**	**10.1**
SD	Random	Spat-temp. cluster	0.257	0.178	0.333	0.104	0.0449	0.156	0.271	0.193	0.348
		Sampling night × Location	0.764	0.733	0.791	0.362	0.341	0.382	0.587	0.567	0.606
		Site ID	0.618	0.534	0.717	0.407	0.352	0.470	0.575	0.493	0.667
		Site ID × Year	0.247	0.204	0.299	0.159	0.131	0.189	0.246	0.202	0.297
	Spline	Day of year	13.	8.65	20.3	3.75	2.33	6.16	10.0	6.65	15.9
		Hours active	1.91	0.900	3.62	0.947	0.292	2.29	1.84	0.836	3.64

Nr. of traps: Number of traps
Sampl. prev. night: Sampling in previous night
Spat.-temp. cluster: Spatio-temporal cluster

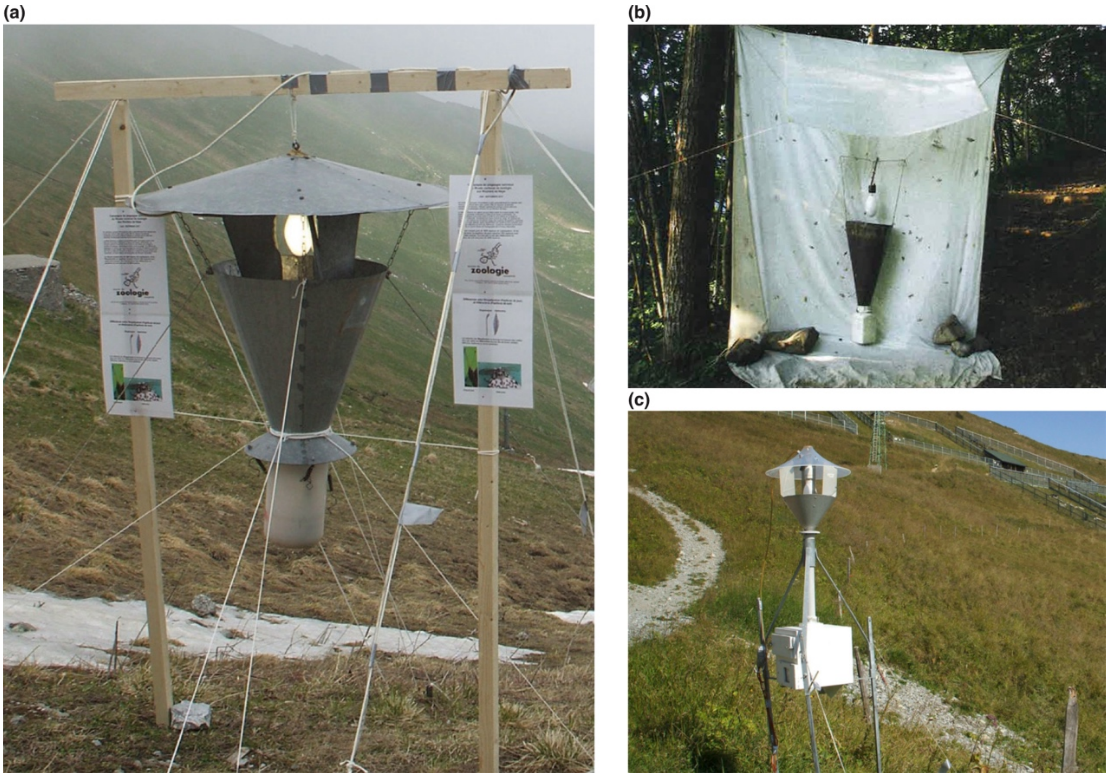

Fig. S1 Photographs of the different trap types that were used. (**a**), (**c**) Fixed traps, for which two different types were in place (type 1: panel a; type 2: panel c). (**b**) Manual trap. Photographs from Rezbanyai-Reser, L. (2014). Zur Nachtgrossfalterfauna von drei Lebensraumkomplexen im Kanton Waadt, Westschweiz. – Rochers de Naye, 1970m in den Alpen, sowie Chalet de la Dôle, 1430m und La Barillette, 1450m im Jura (Lepidoptera: „Macroheterocera“). *Lepidopterologische Mitteilungen Aus Luzern, 14, 1–96* and from Rezbanyai-Reser, L. (2018). Die wichtigsten Lichtfallen- und Lichtfangorte von L. Rezbanyai-Reser in der Schweiz, 1969-2018. *Lepidopterologische Mitteilungen Aus Luzern, 22, 67–72*.

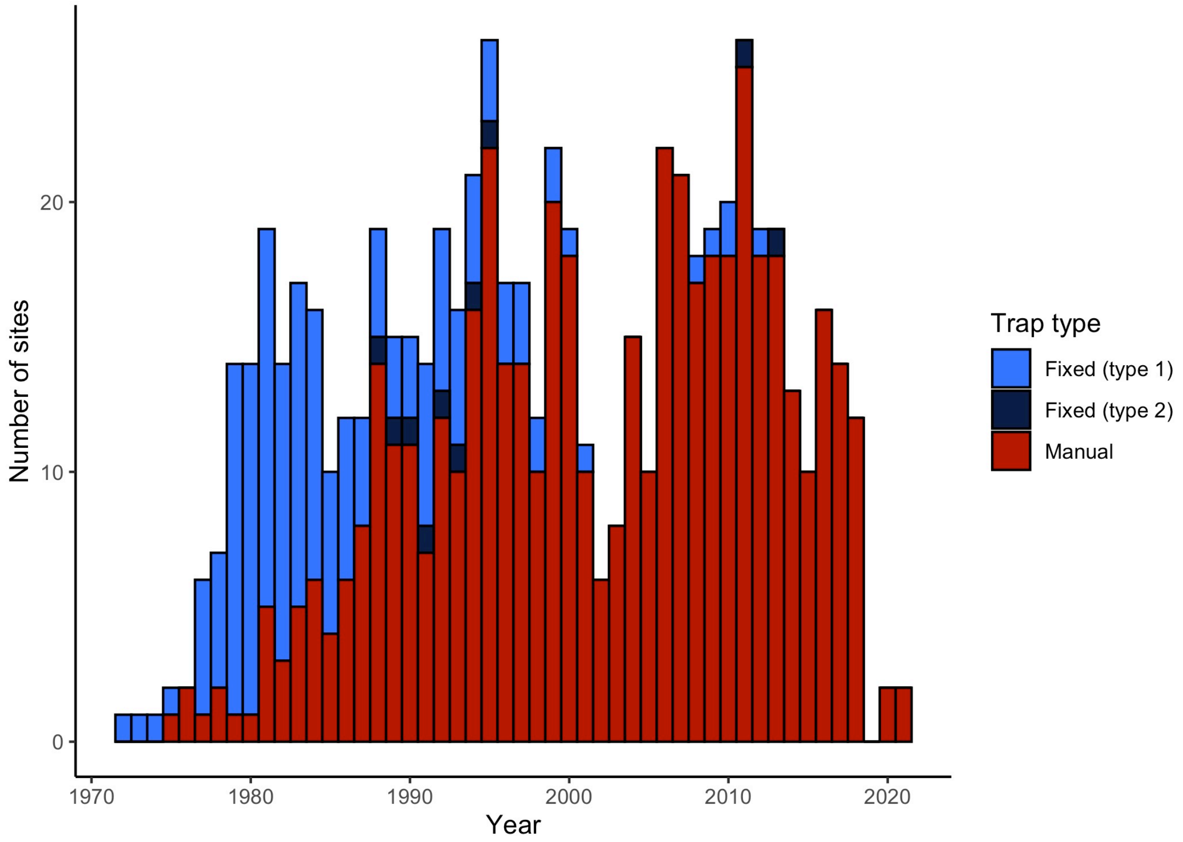

Fig. S2 Number of sites sampled per year. Colours distinguish sites with fixed traps (two different types, blue colours) and manual traps (red).

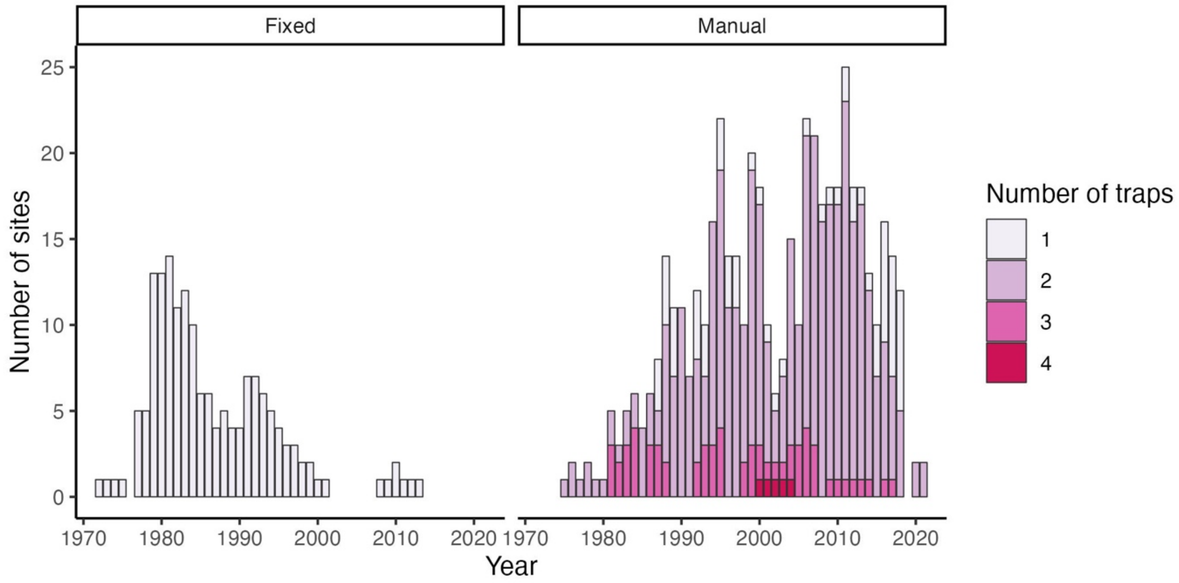

Fig. S3 Number of sites sampled per year, differentiated by number of traps that were in place (between 1 and 4). The left panel shows the locations with fixed traps, the right panel the locations with manual traps.

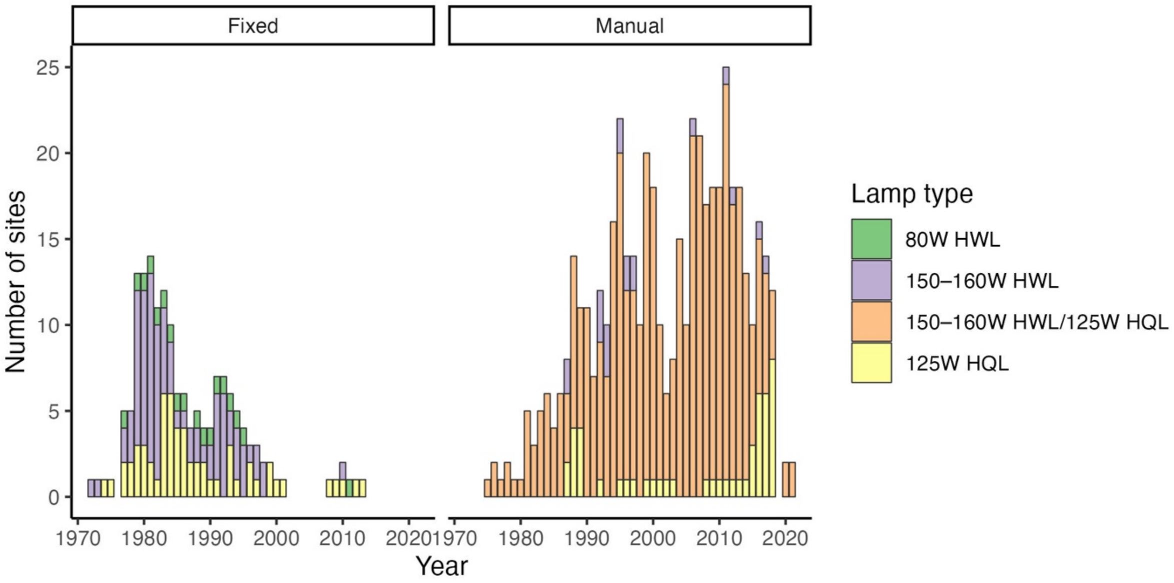

Fig. S4 Number of sites sampled per year, differentiated by lamp types that were in place. The left panel shows the locations with fixed traps, the right panel the locations with manual traps. 80W HWL: 80W mercury mixed-light lamps; 150–160W HWL: 150–160W mercury mixed-light lamps; 125W HQL: 125W mercury vapour lamps.

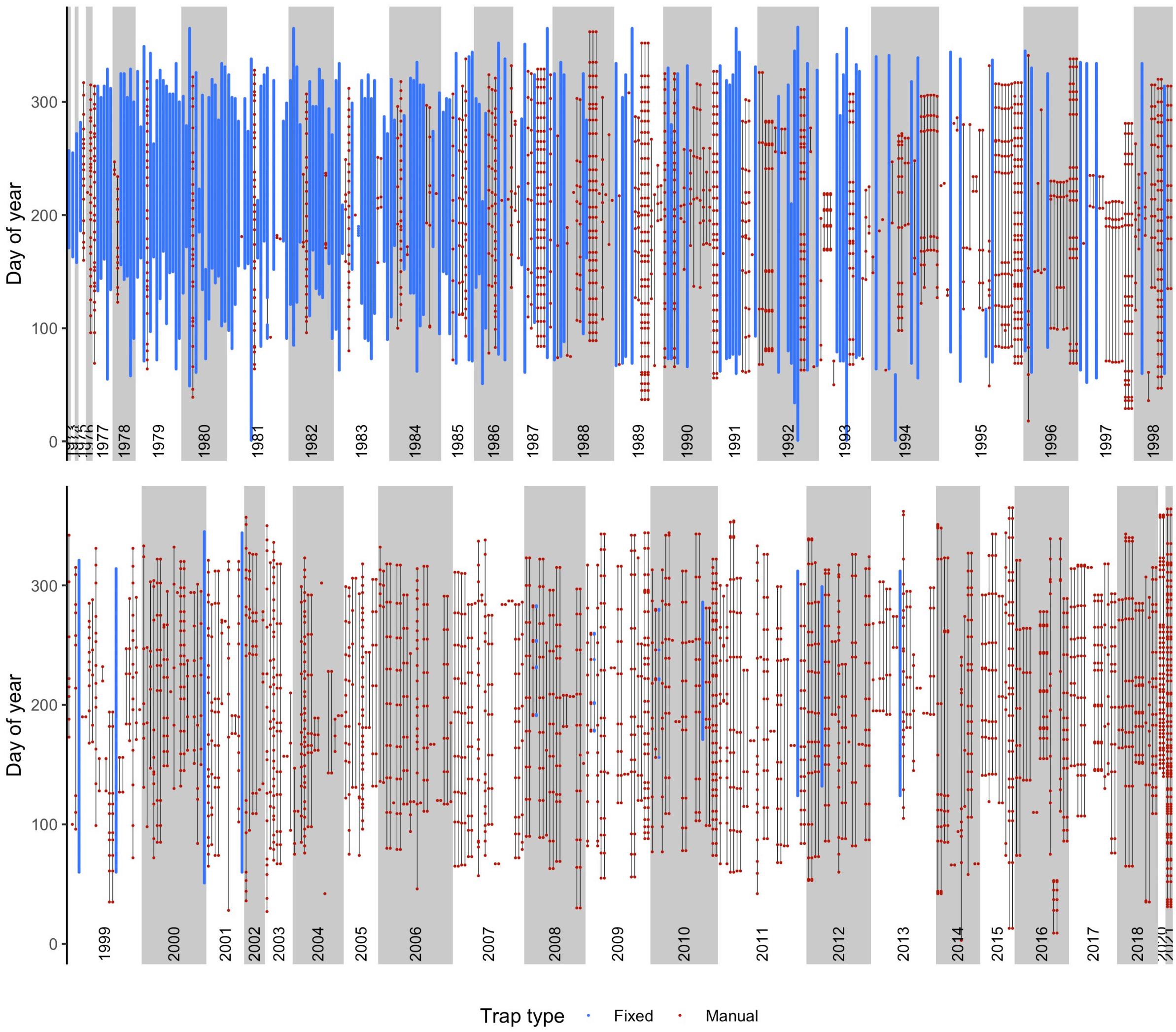

Fig. S5 Overview of all single sampling nights. They are arranged chronologically across the whole study period (1972–2021). Each vertical line connects samples from one site and one year. They are arranged in blocks of single years (different shadings). The *y* axis shows the day of the year (between 1 and 366) of the sampling. Single sampling nights are shown as points (which might overlay for very dense sampling periods), colors differentiate the locations with fixed traps (blue) and locations with manual traps (red).

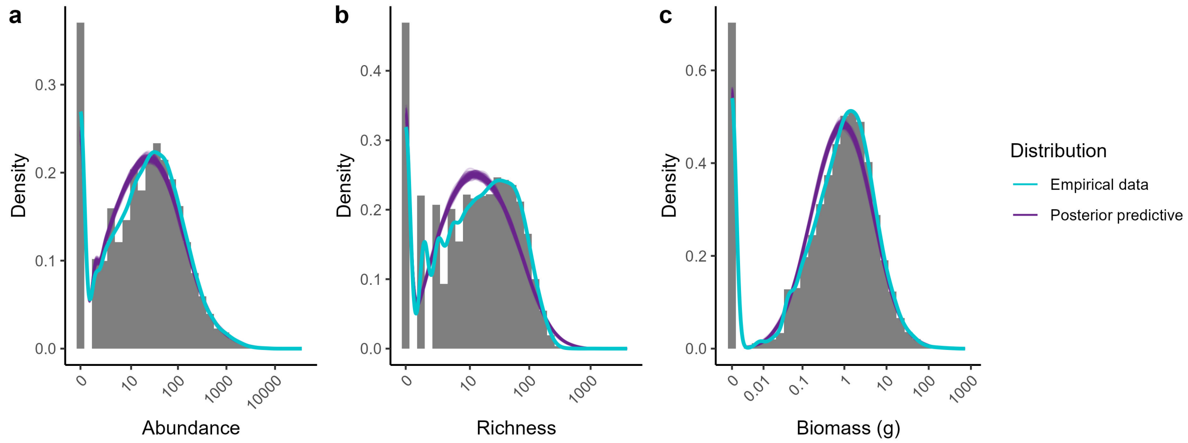

Fig. S6 Empirical data distribution (turquoise) in relation to 100 draws of the posterior predictive distribution (violet) for the models on (**a**) abundance, (**b**) richness and (**c**) biomass of moths. Lines show kernel density estimates, underlying bars are histograms of the empirical data distribution. The *x* axes are on log scale (after adding the minimal non-zero value to all values).

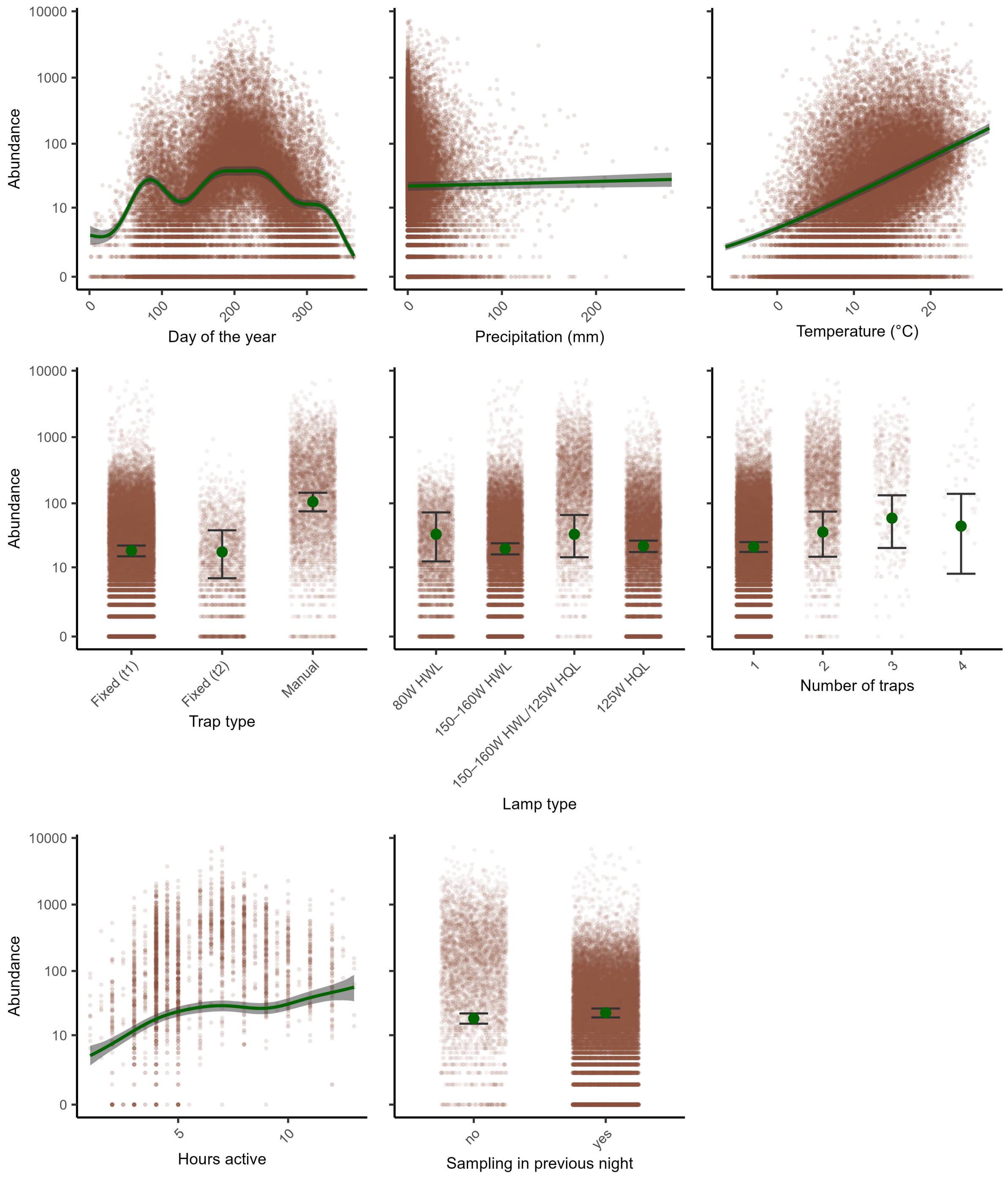

Fig. S7 Conditional effects of model covariates related to sampling timing (day of the year, precipitation and temperature of sampling night) and sampling design (trap type, lamp type, number of traps, sampling duration, sampling in previous night) on moth abundance. The *y* axis shows (estimated) abundance per sampling night. Point estimates are in green, shaded areas show 95% credible intervals. The underlying brown points show raw data per sampling night (n = 35,847). The *y* axes are on log scale (after adding the minimal non-zero value to all values). For detailed model results, see Table S2.

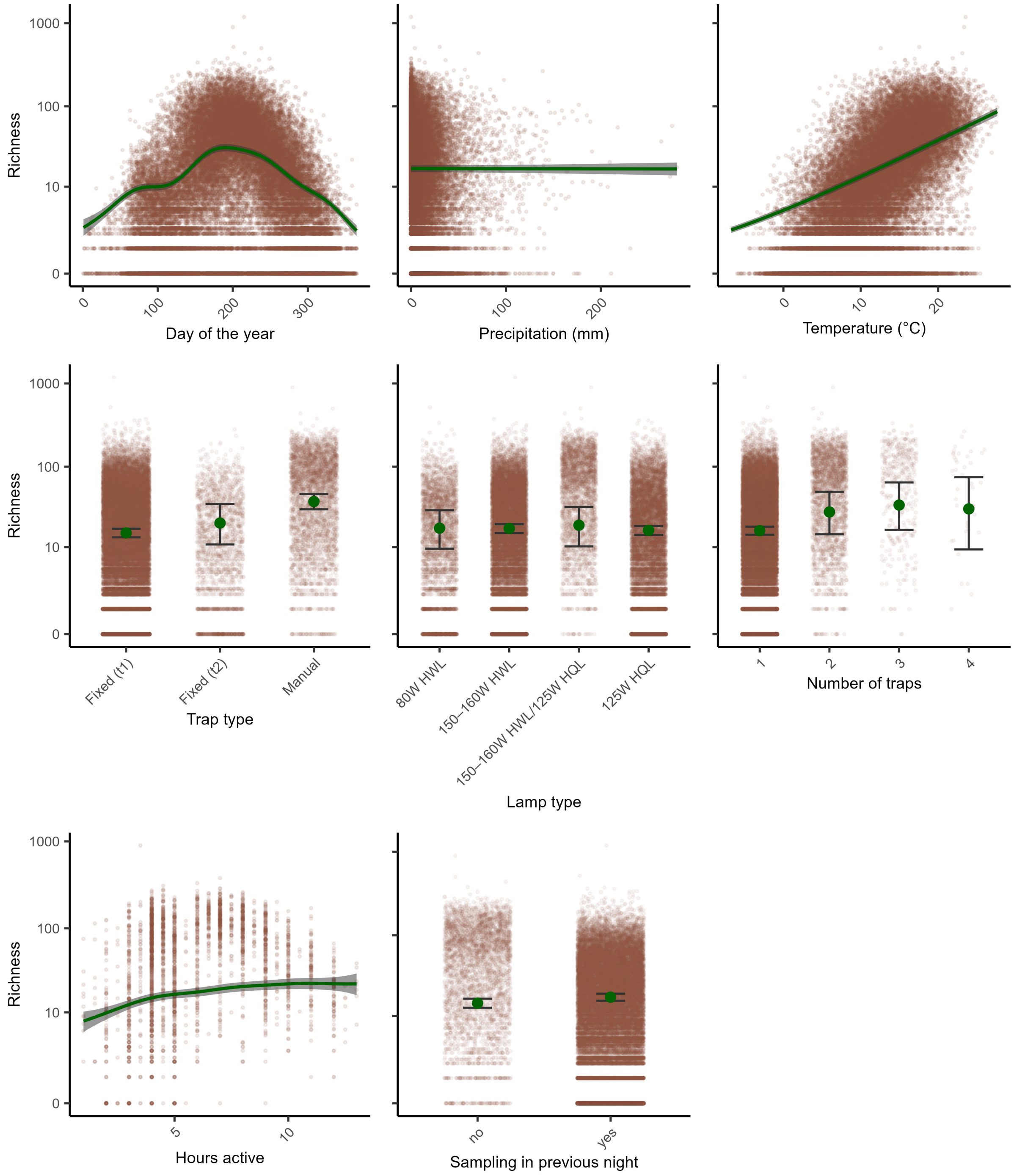

Fig. S8 Conditional effects of model covariates related to sampling timing (day of the year, precipitation and temperature of sampling night) and sampling design (trap type, lamp type, number of traps, sampling duration, sampling in previous night) on moth richness. The *y* axis shows (estimated) abundance per sampling night. Point estimates are in green, shaded areas show 95% credible intervals. The underlying brown points show raw data per sampling night (n = 35,847). The *y* axes are on log scale (after adding the minimal non-zero value to all values). For detailed model results, see Table S2.

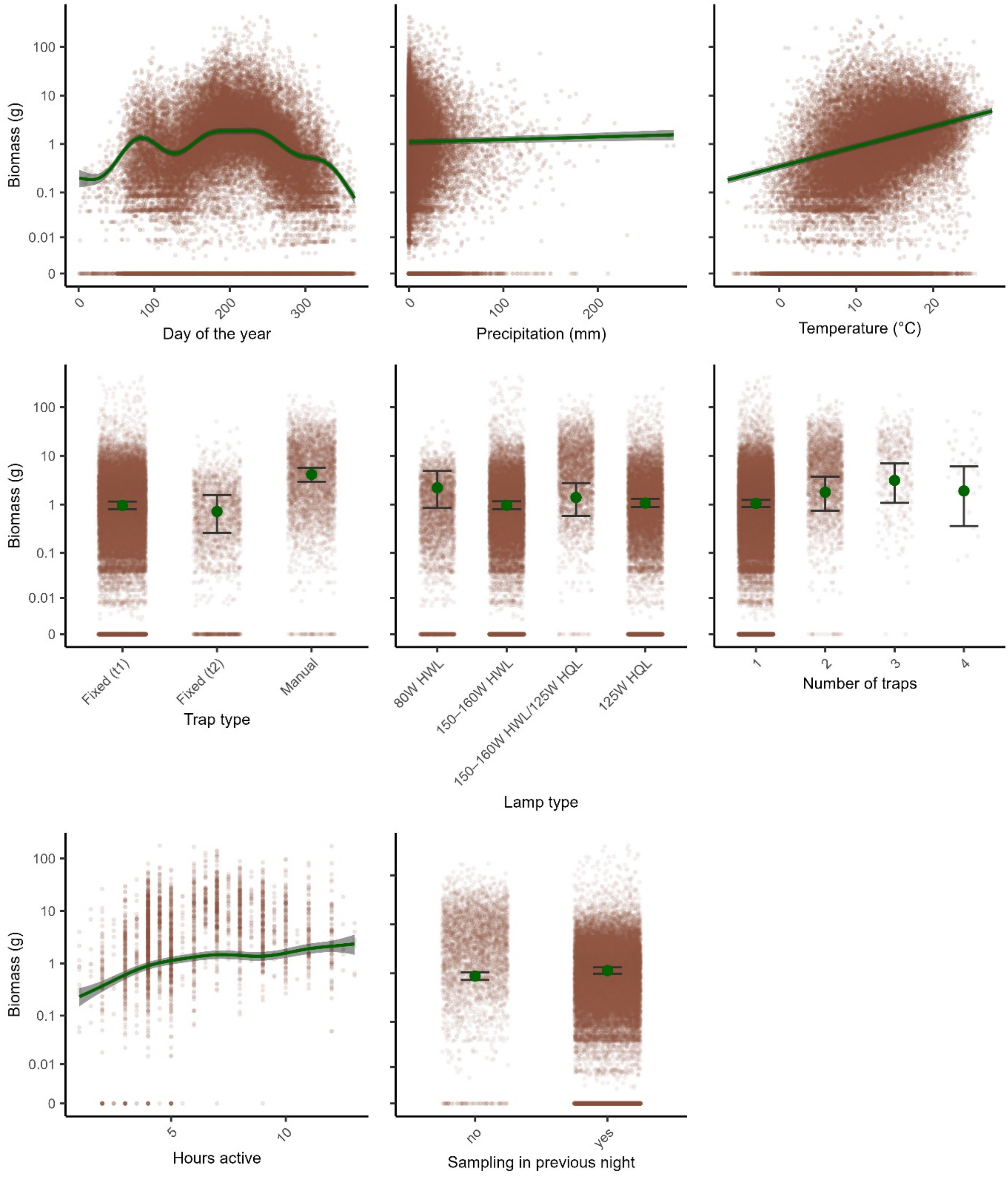

Fig. S9 Conditional effects of model covariates related to sampling timing (day of the year, precipitation and temperature of sampling night) and sampling design (trap type, lamp type, number of traps, sampling duration, sampling in previous night) on estimated moth biomass. The *y* axis shows (estimated) abundance per sampling night. Point estimates are in green, shaded areas show 95% credible intervals. The underlying brown points show raw data per sampling night (n = 35,847). The *y* axes are on log scale (after adding the minimal non-zero value to all values). For detailed model results, see Table S2.

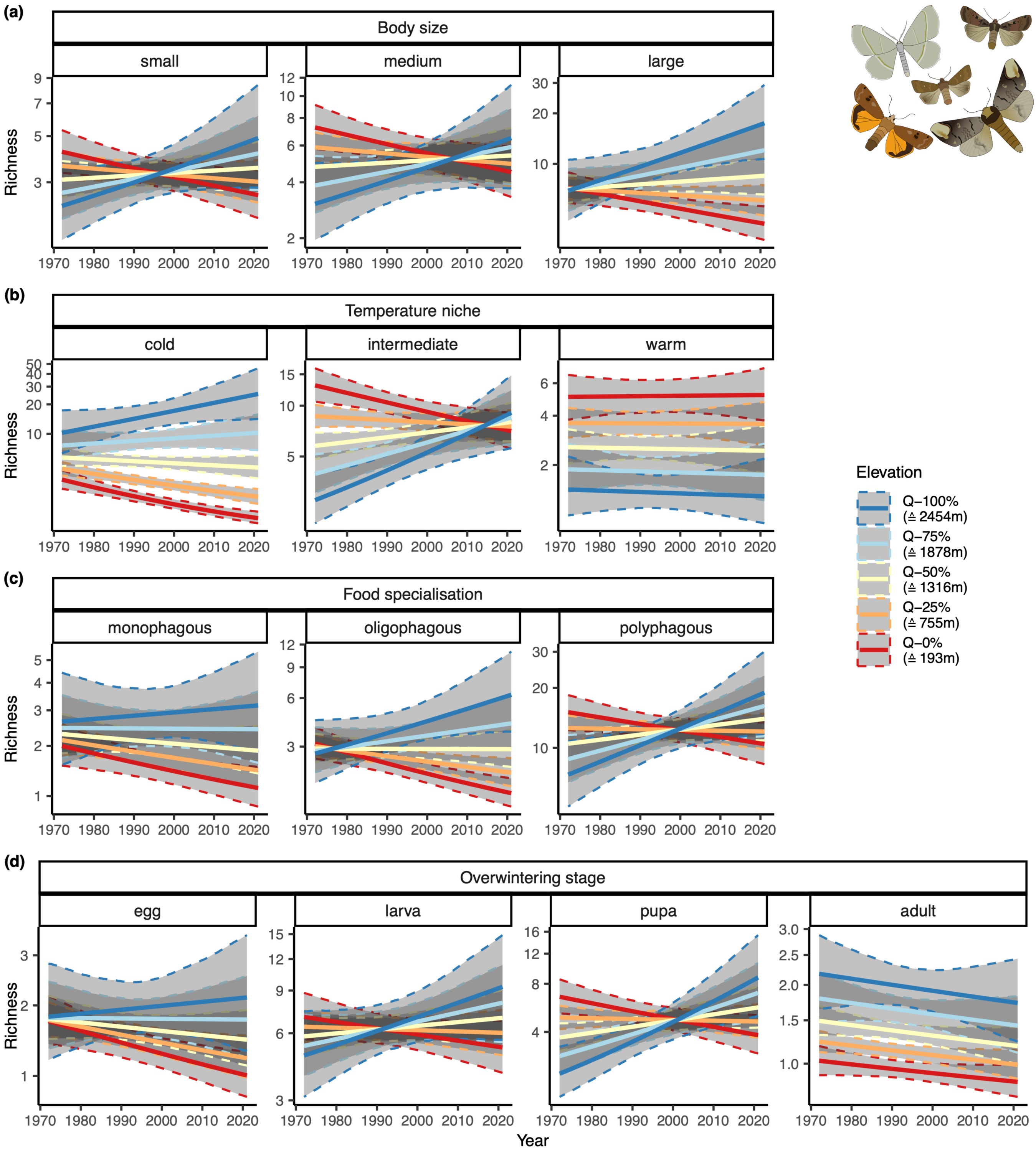

Fig. S10 Change in moth richness across the 50 study years (1972–2021) in relation to the elevation of the study site for different moth groups defined by species traits. Traits are (**a**) body size (mass), (**b**) temperature niche (mean temperature of species occurrence in Europe), (**c**) food specialization (monophagous, oligophagous, polyphagous), and (**d**) overwintering stage (egg, larva, pupa, adult). For continuous traits (body size, temperature niche), groups were built along the 33% and 66% quantiles (one third of the recorded species in each group). Lines are point estimates from model predictions (conditional effects). Separate trend predictions are shown for different quantiles (0%, 25%, 50%, 75%, 100%) of the elevational range. The corresponding elevations are indicated in the legend, e.g., 193 m asl for the 0%-quantile (minimum elevation covered in the data). Shaded areas show 95%-credible intervals for the predictions. Detailed model results in Tables S5, S8, S11, S14.

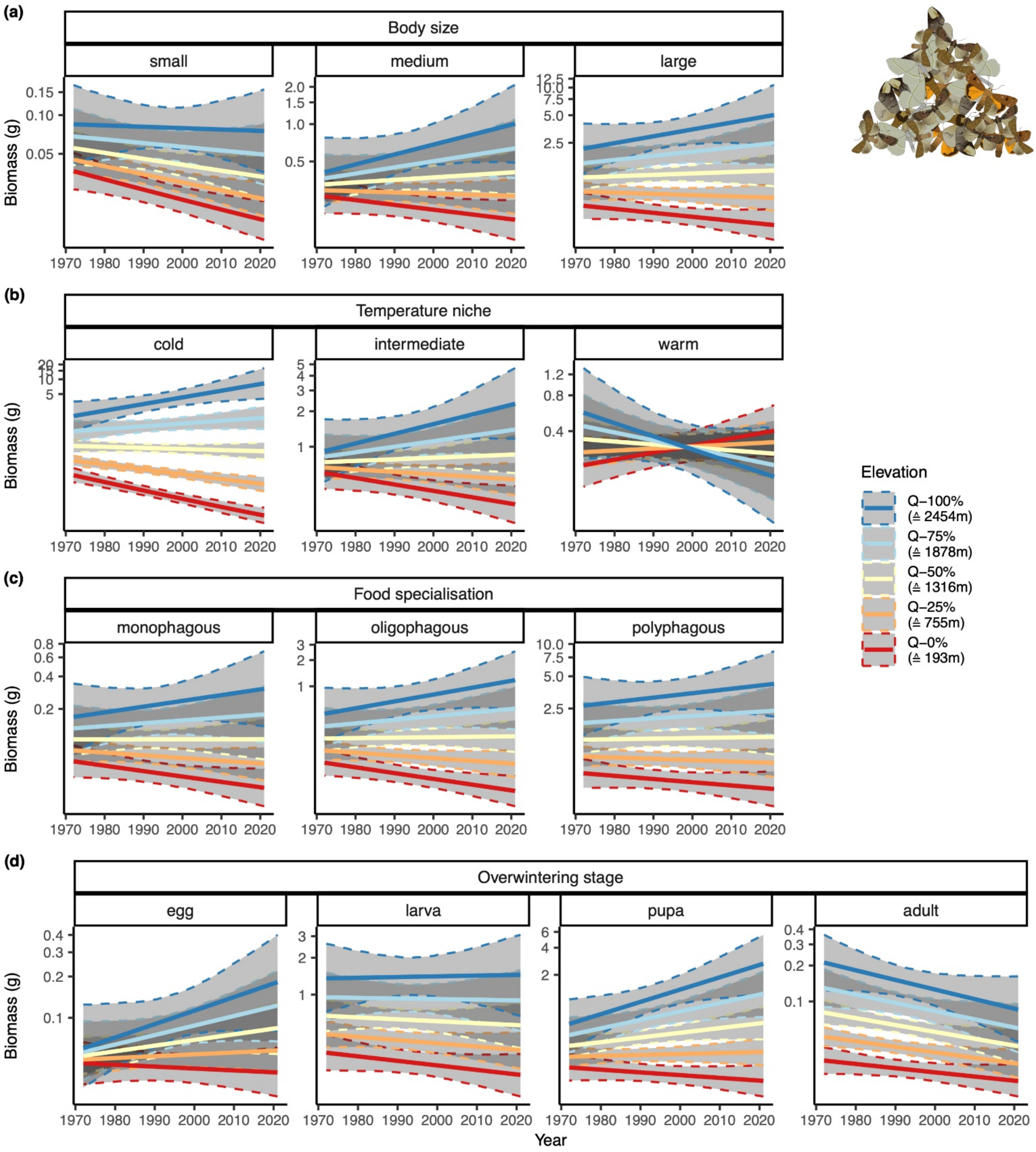

Fig. S11 Change in moth biomass across the 50 study years (1972–2021) in relation to the elevation of the study site for different moth groups defined by species traits. Traits are (**a**) body size (mass), (**b**) temperature niche (mean temperature of species occurrence in Europe), (**c**) food specialization (monophagous, oligophagous, polyphagous), and (**d**) overwintering stage (egg, larva, pupa, adult). For continuous traits (body size, temperature niche), groups were built along the 33% and 66% quantiles (one third of the recorded species in each group). Lines are point estimates from model predictions (conditional effects). Separate trend predictions are shown for different quantiles (0%, 25%, 50%, 75%, 100%) of the elevational range. The corresponding elevations are indicated in the legend, e.g., 193 m asl for the 0%-quantile (minimum elevation covered in the data). Shaded areas show 95%-credible intervals for the predictions. Detailed model results in Tables S6, S9, S12, S15.

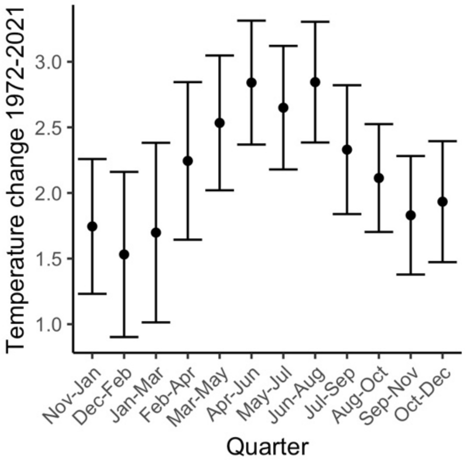

Fig. S12 Estimated mean temperature change between 1972 and 2021 in degree Celsius at the study sites for all possible 3-month-quarters. From linear model predictions based on temperature anomalies (relative to 1962–1980), determined from gridded data provided by MeteoSwiss (1.25 degree minute grid; approx. 2.3 km × 1.6 km). Strongest changes in mean temperature are evident for the quarters Apr–Jun, May–Jul and Jun–Aug.

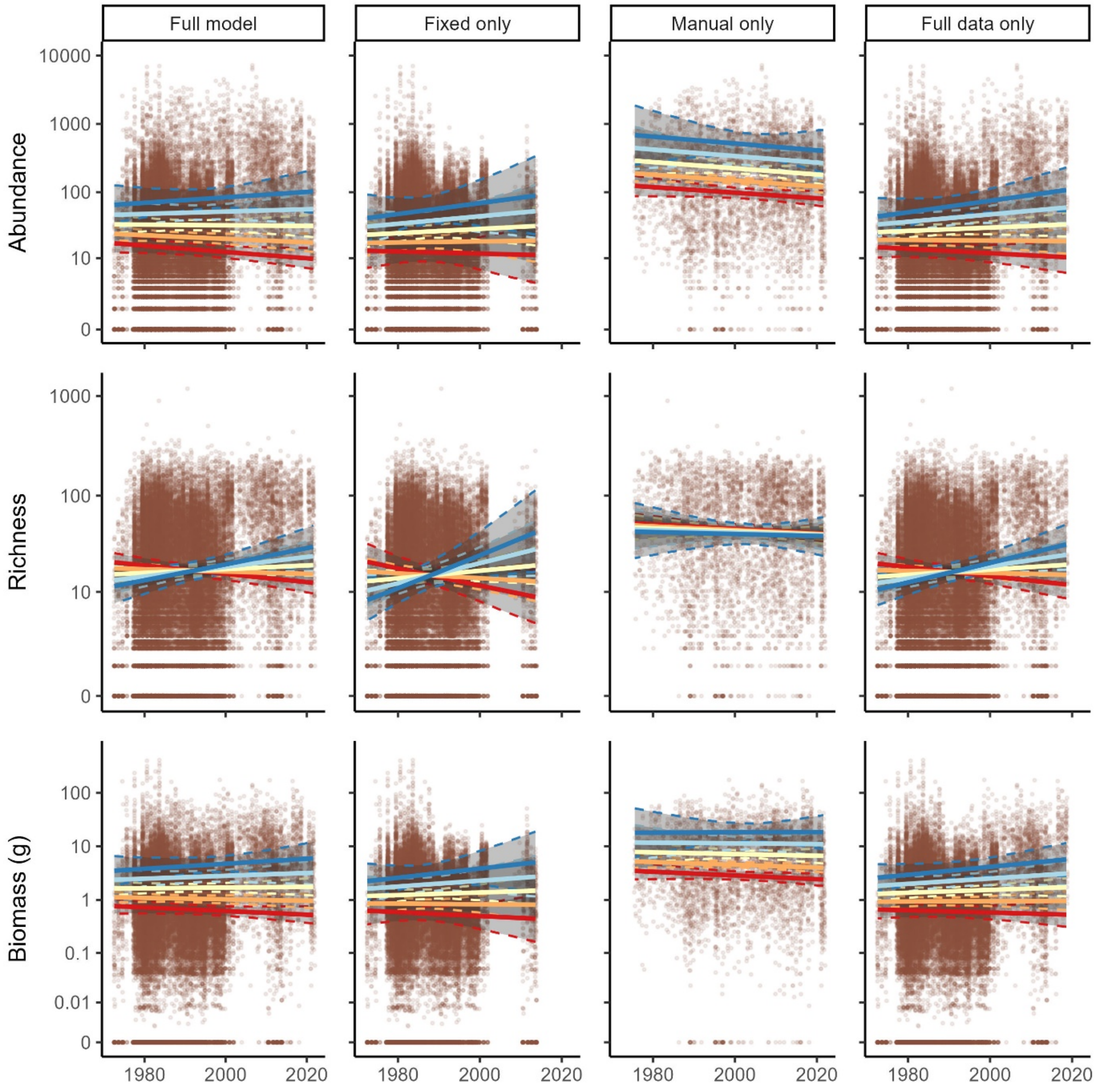

Fig. S13 Change in moth abundance, richness, and biomass across the 50 study years (1972–2021) dependent on elevation for the full dataset, for separate datasets for the two trap types (fixed, manual) and for a dataset not including observations of manual traps missing data on observation time. Lines show model predictions, shaded areas show 95%-credible intervals for the predictions. Underlying points show data per sampling night (n = 35,832 for full model, n = 31,808 for data from fixed traps, n = 4,024 for data from manual traps, n = 34,608 for full data only), with which models were fitted. Note that the y axes are on log scale (after adding the minimal non-zero value to all values).

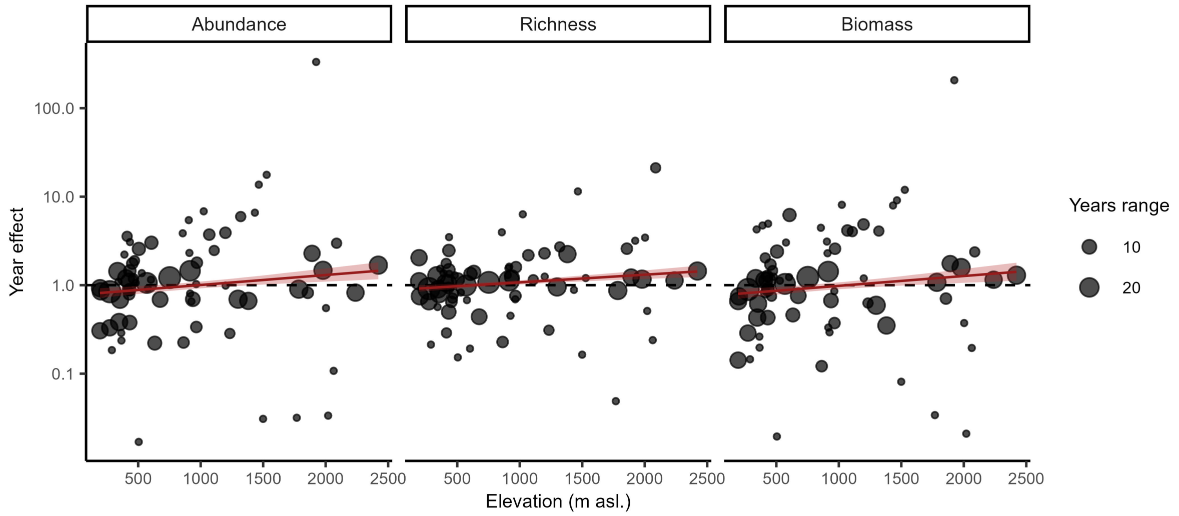

Fig. S14 Relation between the coefficient for year and the elevation in single-site models for abundance, species richness and biomass. Each point represents the parameter estimate for a site, the size represents the range of years covered by the sampling activities at the respective site. The *y* axis is transformed such that it shows the change factor for the respective variable across 10 years. For example, a value of 2 means that abundance that abundance has doubled across 10 study years. The regression lines are based on weighted regression models between the standardised year coefficient and the elevation (range of years as weight) and show the mean and 95%-credible intervals. For abundance, an increase of elevation by 1000m means that the change factor is on average multiplied by 1.30 (95%-CI: 1.14–1.48), for richness by 1.22 (95%-CI: 1.12–1.33) and for biomass by 1.29 (95%-CI: 1.12–1.49).
